# Supplementary material for: Gene interfered-ferroptosis therapy for cancers
Source: Nat Commun. 2021 Sep 7;12:5311. doi: 10.1038/s41467-021-25632-1 (PMC8423858; doi:10.1038/s41467-021-25632-1)
Supplement: Supplementary file 1 — Supplementary information [file 41467_2021_25632_MOESM1_ESM.pdf]

Supplementary Information

Gene interfered-ferroptosis therapy for cancers

Jinliang Gao[1], Tao Luo[1], Jinke Wang[1,*]

[1] *State Key Laboratory of Bioelectronics, Southeast University, Nanjing 210096, China.*

Supplementary Table 1. Target of Cas13a-gRNA and miRNA.

Name	gRNA target (5′-3′)	miRNA target (5′-3′)
Human FPN	GGGAGATCGGATGTGGCACTTTGCGGTG	ATCATGTAAGCTGCAGGTAGA
Human LCN2	AGTTCACGCTGGGCAACATTAAGAGTTA	AGTTCACGCTGGGCAACATTA
Mouse FPN	ATGGGAGCATCAGCAATAACTGGAATAA	GCAAATCAGTGAGTCTGTATA
Mouse LCN2	CATCATCTTCTCTGTCCCCACCGACCAA	AGGACTCAACTCAGAACTTGA
NT	ATGGATTACTTGGTAGAACAGCAATCTA	GTCTCCACGCGCAGTACATTT

Supplementary Table 2. Oligonucleotides sequences used to construct gRNA expression vector.

Name	Sequence (5′-3′)
Human FPN-F	AGGAAGACTAAAACCACCGCAAAGTGCCACATCCGATCTCCCTTTTGCGTCTTCGA
Human FPN-R	TCGAAGACGCAAAAGGGAGATCGGATGTGGCACTTTGCGGTGGTTTTAGTCTTCCT
Mouse FPN-F	AGGAAGACTAAAACTTATTCCAGTTATTGCTGATGCTCCCATTTTTGCGTCTTCGA
Mouse FPN-R	TCGAAGACGCAAAAATGGGAGCATCAGCAATAACTGGAATAAGTTTTAGTCTTCCT
Human LCN2-F	AGGAAGACTAAAACTAACTCTTAATGTTGCCCAGCGTGAACTTTTTGCGTCTTCGA
Human LCN2-R	TCGAAGACGCAAAAAGTTCACGCTGGGCAACATTAAGAGTTAGTTTTAGTCTTCCT
Mouse LCN2-F	AGGAAGACTAAAACTTGGTCGGTGGGGACAGAGAAGATGATGTTTTGCGTCTTCGA
Mouse LCN2-R	TCGAAGACGCAAAACATCATCTTCTCTGTCCCCACCGACCAAGTTTTAGTCTTCCT
NT-F	AGGAAGACTAAAACTAGATTGCTGTTCTACCAAGTAATCCATTTTTGCGTCTTCGA
NT-R	TCGAAGACGCAAAAATGGATTACTTGGTAGAACAGCAATCTAGTTTTAGTCTTCCT

Supplementary Table 3. Oligonucleotides sequences used to construct miRNA expression vector.

Name	Sequence (5′-3′)
Human miR-FPN-F	TGCTGTCTACCTGCAGCTTACATGATGTTTTGGCCACTGACTGACATCATGTACTGCAGGTAGA
Human miR-FPN-R	CCTGTCTACCTGCAGTACATGATGTCAGTCAGTGGCCAAAACATCATGTAAGCTGCAGGTAGAC
Mouse miR-FPN-F	TGCTGTATACAGACTCACTGATTTGCGTTTTGGCCACTGACTGACGCAAATCAGAGTCTGTATA
Mouse miR-FPN-R	CCTGTATACAGACTCTGATTTGCGTCAGTCAGTGGCCAAAACGCAAATCAGTGAGTCTGTATAC
Human miR-LCN2-F	TGCTGTAATGTTGCCCAGCGTGAACTGTTTTGGCCACTGACTGACAGTTCACGGGGCAACATTA
Human miR-LCN2-R	CCTGTAATGTTGCCCCGTGAACTGTCAGTCAGTGGCCAAAACAGTTCACGCTGGGCAACATTAC
Mouse miR-LCN2-F	TGCTGTCAAGTTCTGAGTTGAGTCCTGTTTTGGCCACTGACTGACAGGACTCATCAGAACTTGA
Mouse miR-LCN2-R	CCTGTCAAGTTCTGATGAGTCCTGTCAGTCAGTGGCCAAAACAGGACTCAACTCAGAACTTGAC
miR-NT-F	TGCTGAAATGTACTGCGCGTGGAGACGTTTTGGCCACTGACTGACGTCTCCACGCAGTACATTT
miR-NT-R	CCTGAAATGTACTGCGTGGAGACGTCAGTCAGTGGCCAAAACGTCTCCACGCGCAGTACATTTC

Supplementary Table 4. Primer sequences used for qPCR.

Name	Primer sequence (5′-3′)	Name	Primer sequence (5′-3′)
AAV-F	TGCATGACCAGGCTCAGCTA	AAV-R	GACAGGGAAGGGAGCAGTG
Cas13a-F	GGAAAAGTACCAGTCCGCCA	Cas13a-R	GAAGTCCAGGAACTTGCCGA
GAPDH-F	ATTTGGTCGTATTGGGCG	GAPDH-R	CTCGCTCCTGGAAGATGG
Human RELA-F	CCTGGAGCAGGCTATCAGTC	Human RELA-R	ATGGGATGAGAAAGGACAGG
Mouse RELA-F	TGCGATTCCGCTATAAATGCG	Mouse RELA-R	ACAAGTTCATGTGGATGAGGC
Human GAPDH-F	ATTTGGTCGTATTGGGCG	Human GAPDH-R	CTCGCTCCTGGAAGATGG
Mouse GAPDH-F	TCACCACCATGGAGAAGGC	Mouse GAPDH-R	GCTAAGCAGTTGGTGGTGCA
Human FPN-F	CACAACCGCCAGAGAGGATG	Human FPN-R	CACATCCGATCTCCCCAAGT
Human LCN2-F	CCCGCAAAAGATGTATGCCA	Human LCN2-R	CTCACCACTCGGACGAGGTA
Mouse FPN-F	TGGAACTCTATGGAAACAGCCT	Mouse FPN-R	TGGCATTCTTATCCACCCAGT
Mouse LCN2-F	TGGCCCTGAGTGTCATGTG	Mouse LCN2-R	CTCTTGTAGCTCATAGATGGTGC
Mouse CD31-F	ACCGGGTGCTGTTCTATAAGG	Mouse CD31-R	TCACCTCGTACTCAATCGTGG
Mouse MKi67-F	CAAGGCGAGCCTCAAGAGATA	Mouse MKi67-R	TGTGCTGTTCTACATGCCCTG
Mouse Tyrp1-F	ACTTGATGGGATCCAGAAGC	Mouse Tyrp1-R	CTGATTGGTCCACCCTCAGT

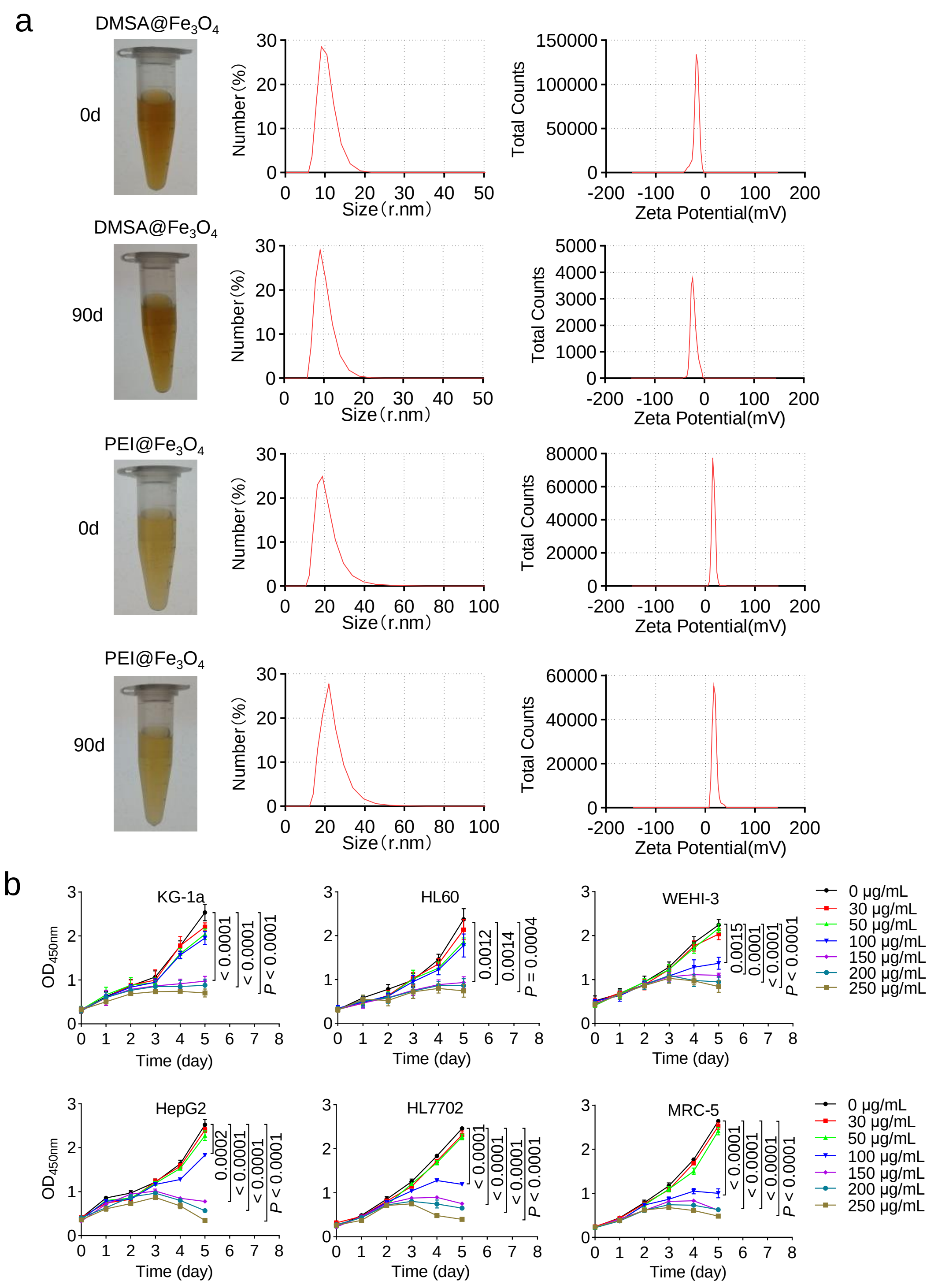

Supplementary Figure 1. Stability of the DMSA-coated Fe_3O_4 nanoparticles (DMSA@Fe_3O_4) and PEI-coated Fe_3O_4 nanoparticles (PEI@Fe_3O_4) used in this study. a, Hydrated particle size and zeta potential of DMSA@Fe_3O_4 (FeNP) and PEI@Fe_3O_4 (FeNC) at day 0 and day 90, respectively. **b,** Viability of cells treated by DMSA@Fe_3O_4 (FeNP) at various concentrations. Data are presented as mean ± standard deviation (SD) (*n* = 3 biologically independent samples) and analyzed by two-way ANOVA with Tukey's test.

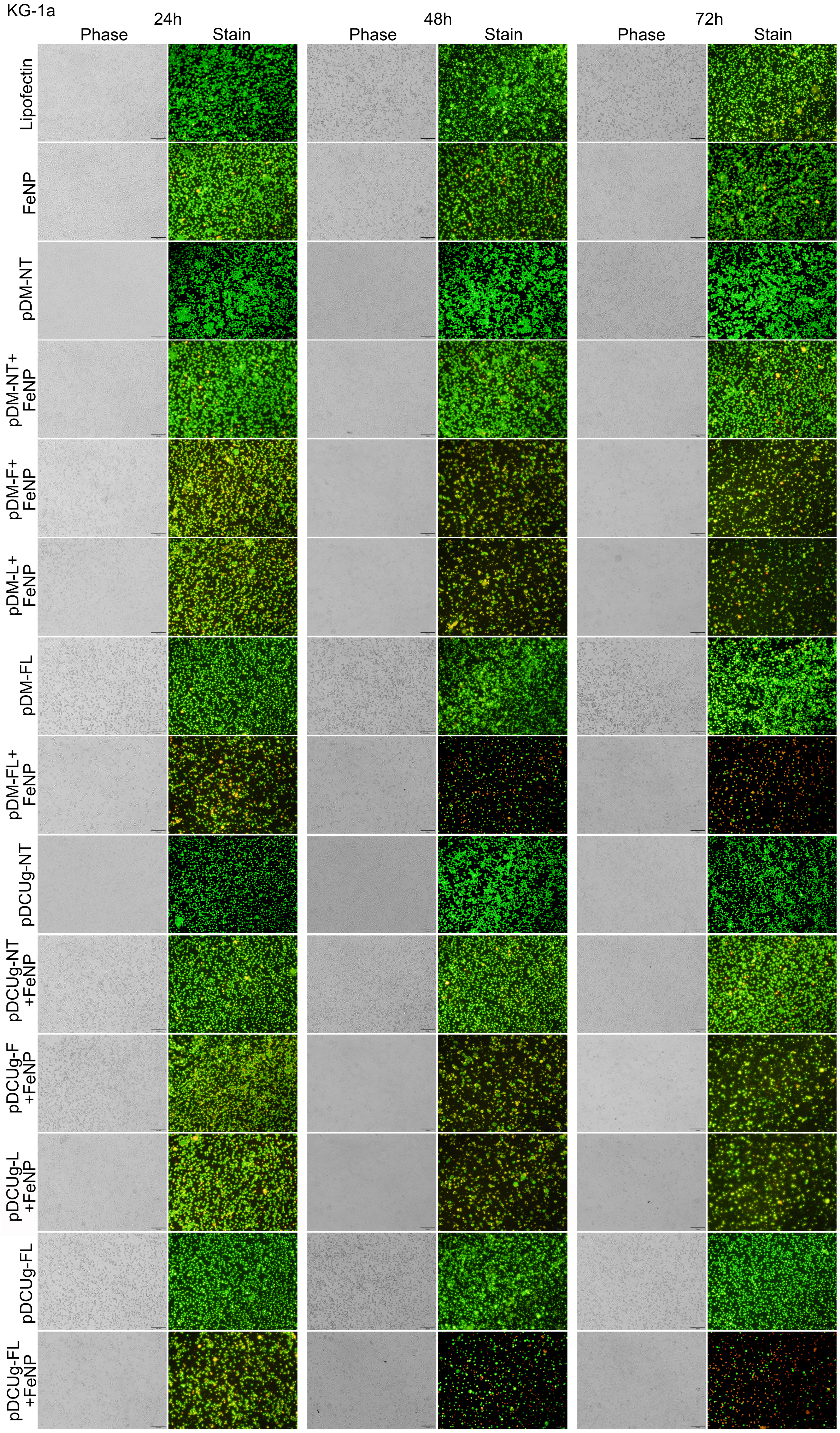

KG-1a
24h
48h
72h
Phase
Stain
Phase
Stain
Phase
Stain
Lipofectin
FeNP
pDM-NT
pDM-NT+ FeNP
pDM-F+ FeNP
pDM-L+ FeNP
pDM-FL
pDM-FL+ FeNP
pDCUg-NT
pDCUg-NT +FeNP
pDCUg-F +FeNP
pDCUg-L +FeNP
pDCUg-FL
pDCUg-FL +FeNP

Supplementary Figure 2. Treatment of KG-1a cells with gene interfering vectors and FeNP. Cells were transfected by various plasmids, cultured for 24 h and then incubated with or without 50 μg/mL of FeNP, and cells were cultured for another 72 h. At 24 h, 48 h and 72 h post FeNP administration, cells were stained with acridine orange and ethidium bromide and imaged under a fluorescence microscope. Scale bars, 100 μm. Representative data of two independent experiments. pDM-NT/pDM-F/pDM-L/pDM-FL, plasmids expressing miRNAs under the control of DMP targeting no, *FPN*, *LCN2*, or *FPN&LCN2* transcripts; pDCUg-NT/pDCUg-F/pDCUg-L/pDCUg-FL, plasmids expressing Cas13a under the control of DMP and gRNAs under the control of U6 promoter targeting no, *FPN*, *LCN2*, or *FPN&LCN2* transcripts.

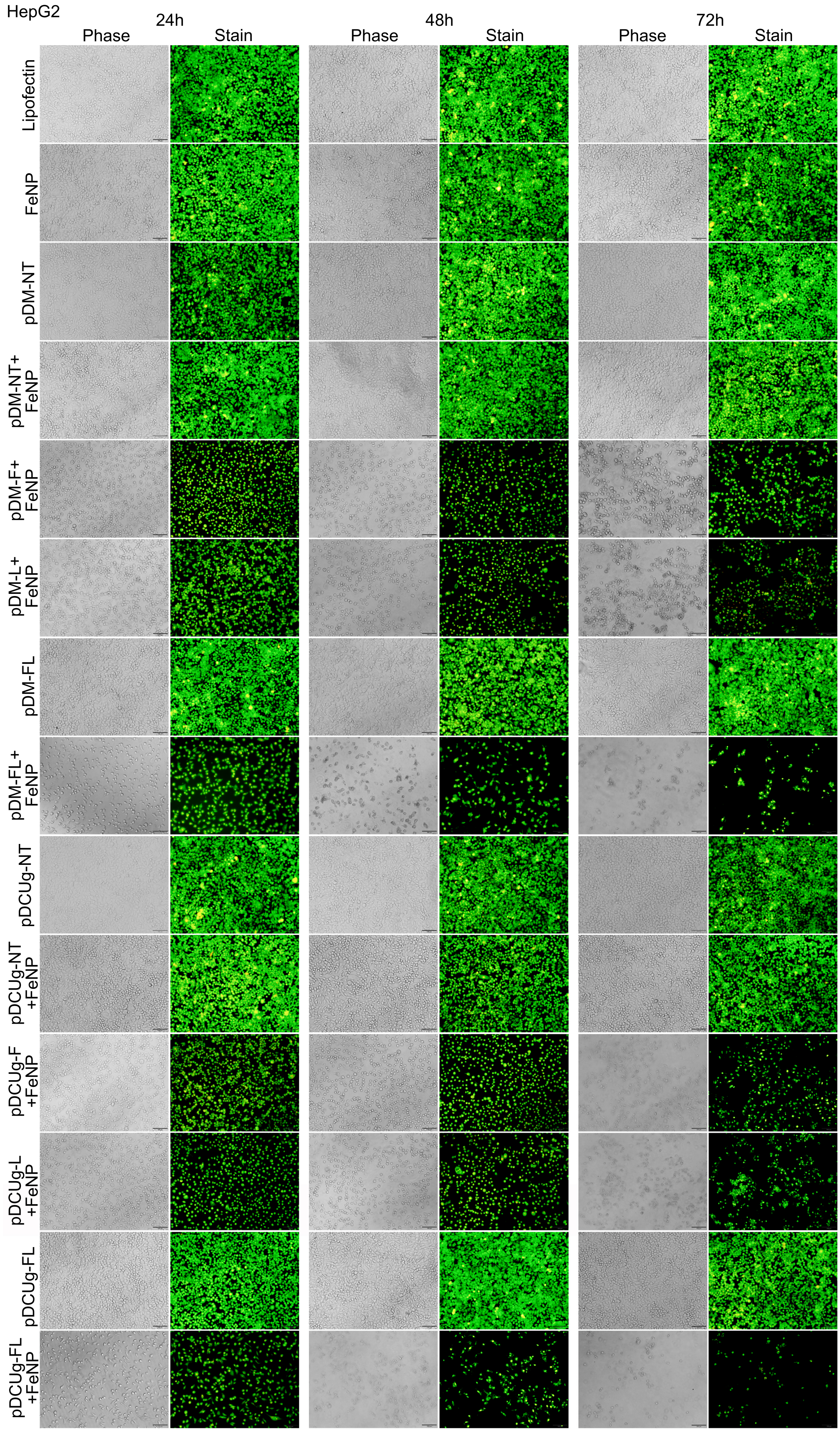
HepG2
24h
48h
72h
Phase
Stain
Phase
Stain
Phase
Stain
Lipofectin
FeNP
pDM-NT
pDM-NT+ FeNP
pDM-F+ FeNP
pDM-L+ FeNP
pDM-FL
pDM-FL+ FeNP
pDCUg-NT
pDCUg-NT +FeNP
pDCUg-F +FeNP
pDCUg-L +FeNP
pDCUg-FL
pDCUg-FL +FeNP

Supplementary Figure 3. Treatment of HepG2 cells with gene interfering vectors and FeNP. Cells were transfected by various plasmids, cultured for 24 h and then incubated with or without 50 μg/mL of FeNP, and cells were cultured for another 72 h. At 24 h, 48 h and 72 h post FeNP administration, cells were stained with acridine orange and ethid-ium bromide and imaged under a fluorescence microscope.Scale bars, 100 μm. Repre-sentative data of two independent experiments. pDM-NT/pDM-F/pDM-L/pDM-FL, plasmids expressing miRNAs under the control of DMP targeting no, *FPN*, *LCN2*, or *FPN&LCN2* transcripts; pDCUg-NT/pDCUg-F/pDCUg-L/pDCUg-FL, plasmids expressing Cas13a under the control of DMP and gRNAs under the control of U6 promoter targeting no, *FPN*, *LCN2*, or *FPN&LCN2* transcripts.

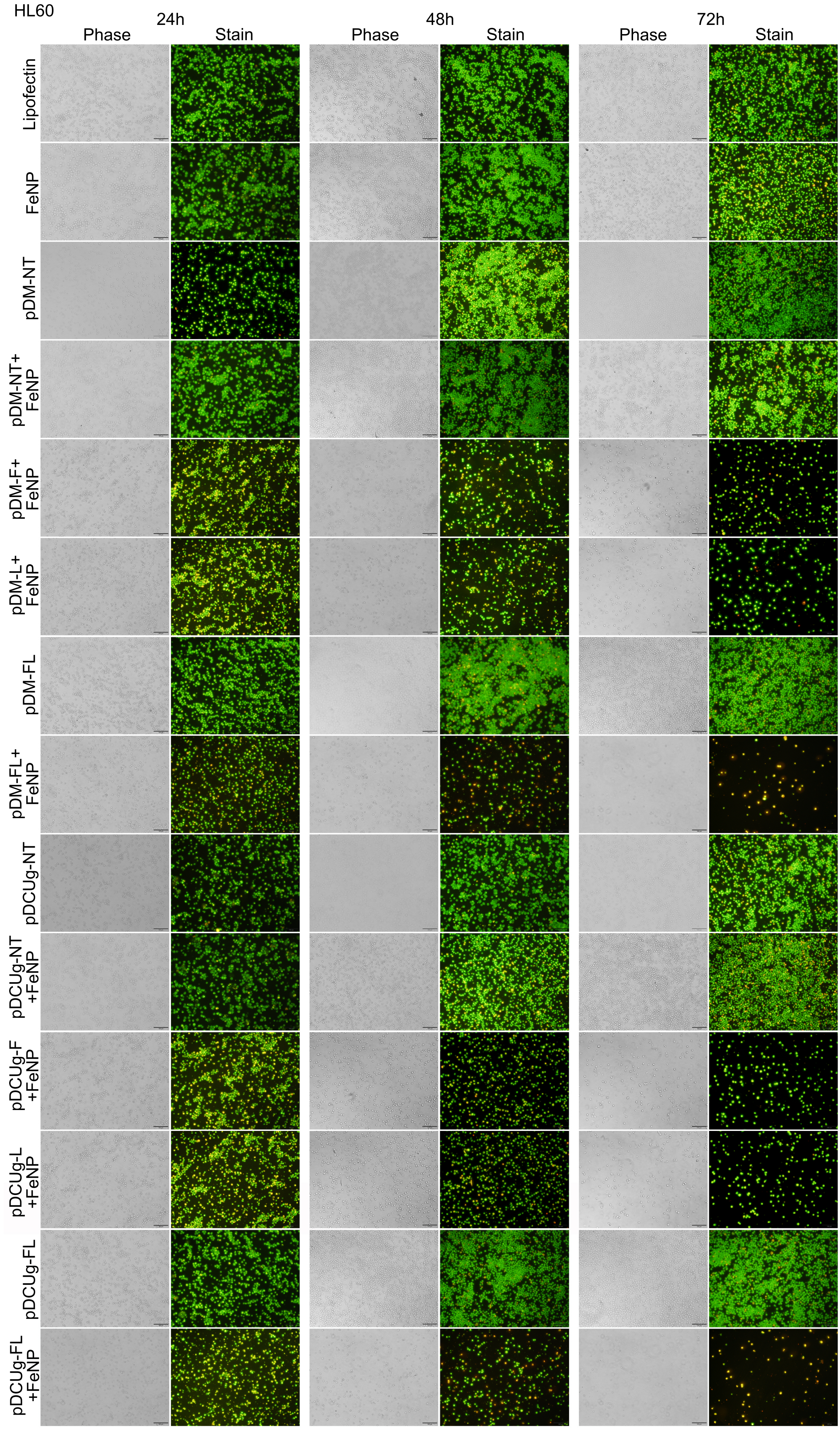

HL60
24h
48h
72h
Phase
Stain
Phase
Stain
Phase
Stain
Lipofectin
FeNP
pDM-NT
pDM-NT+ FeNP
pDM-F+ FeNP
pDM-L+ FeNP
pDM-FL
pDM-FL+ FeNP
pDCUg-NT
pDCUg-NT +FeNP
pDCUg-F +FeNP
pDCUg-L +FeNP
pDCUg-FL
pDCUg-FL +FeNP

Supplementary Figure 4. Treatment of HL60 cells with gene interfering vectors and FeNP. Cells were transfected by various plasmids, cultured for 24 h and then incubated with or without 50 μg/mL of FeNP, and cells were cultured for another 72 h. At 24 h, 48 h and 72 h post FeNP administration, cells were stained with acridine orange and ethid-ium bromide and imaged under a fluorescence microscope. Scale bars, 100 μm. Repre-sentative data of two independent experiments. pDM-NT/ pDM-F/pDM-L/pDM-FL, plasmids expressing miRNAs under the control of DMP targeting no, *FPN*, *LCN2*, or *FPN&LCN2* transcripts; pDCUg-NT/pDCUg-F/ pDCUg-L/pDCUg-FL, plasmids expressing Cas13a under the control of DMP and gRNAs under the control of U6 promoter targeting no, *FPN*, *LCN2*, or *FPN&LCN2* transcripts.

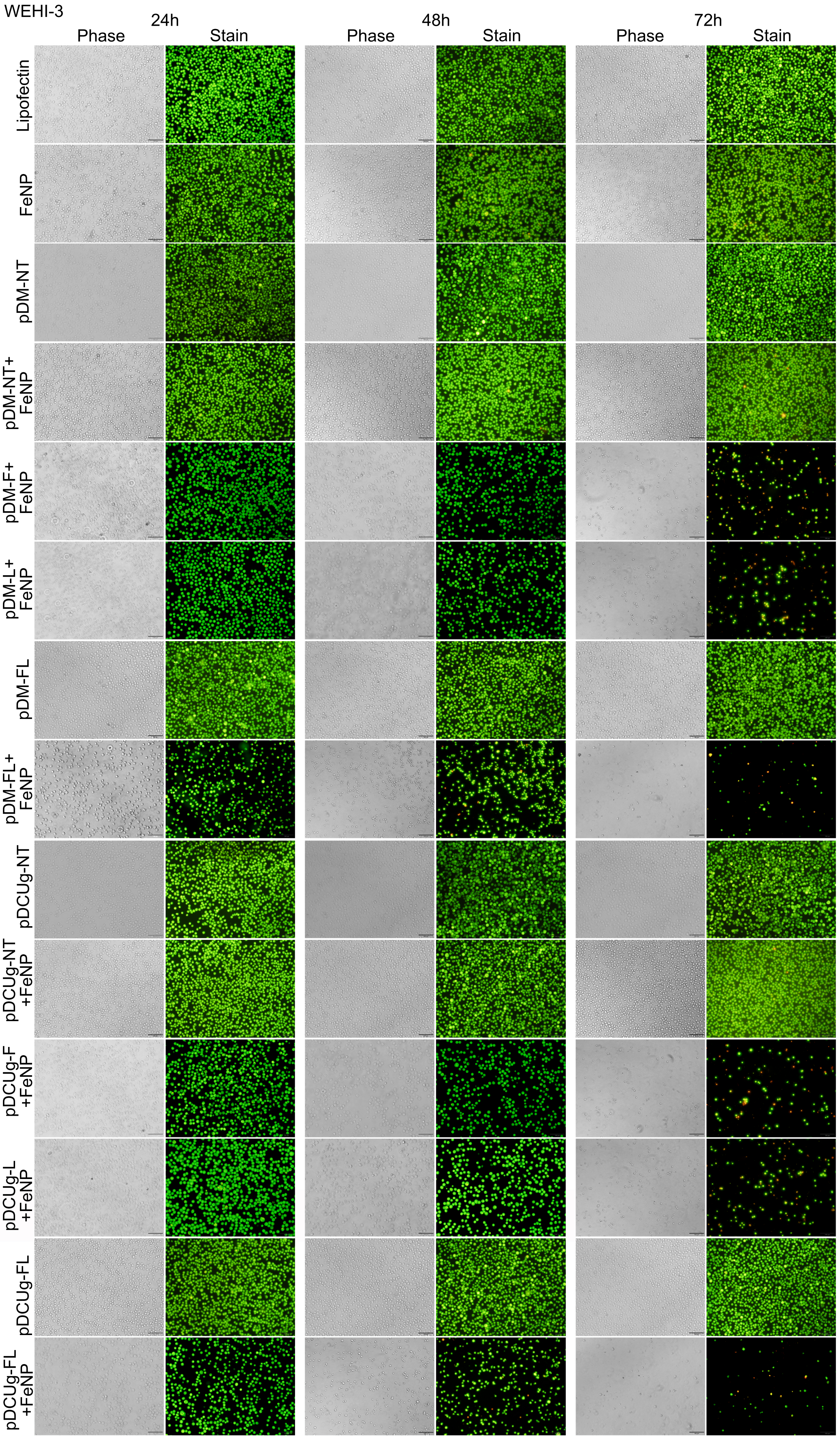

WEHI-3
24h
48h
72h
Phase
Stain
Phase
Stain
Phase
Stain
Lipofectin
FeNP
pDM-NT
pDM-NT+ FeNP
pDM-F+ FeNP
pDM-L+ FeNP
pDM-FL
pDM-FL+ FeNP
pDCUg-NT
pDCUg-NT +FeNP
pDCUg-F +FeNP
pDCUg-L +FeNP
pDCUg-FL
pDCUg-FL +FeNP

Supplementary Figure 5. Treatment of WEHI-3 cells with gene interfering vectors and FeNP. Cells were transfected by various plasmids, cultured for 24 h and then incubated with or without 50 μg/mL of FeNP, and cells were cultured for another 72 h. At 24 h, 48 h and 72 h post FeNP administration, cells were stained with acridine orange and ethidium bromide and imaged under a fluorescence microscope. Scale bars, 100 μm. Representative data of two independent experiments. pDM-NT/pDM-F/pDM-L/pDM-FL, plasmids expressing miRNAs under the control of DMP targeting no, *FPN*, *LCN2*, or *FPN&LCN2* transcripts; pDCUg-NT/pDCUg-F/pDCUg-L/pDCUg-FL, plasmids expressing Cas13a under the control of DMP and gRNAs under the control of U6 promoter targeting no, *FPN*, *LCN2*, or *FPN&LCN2* transcripts.

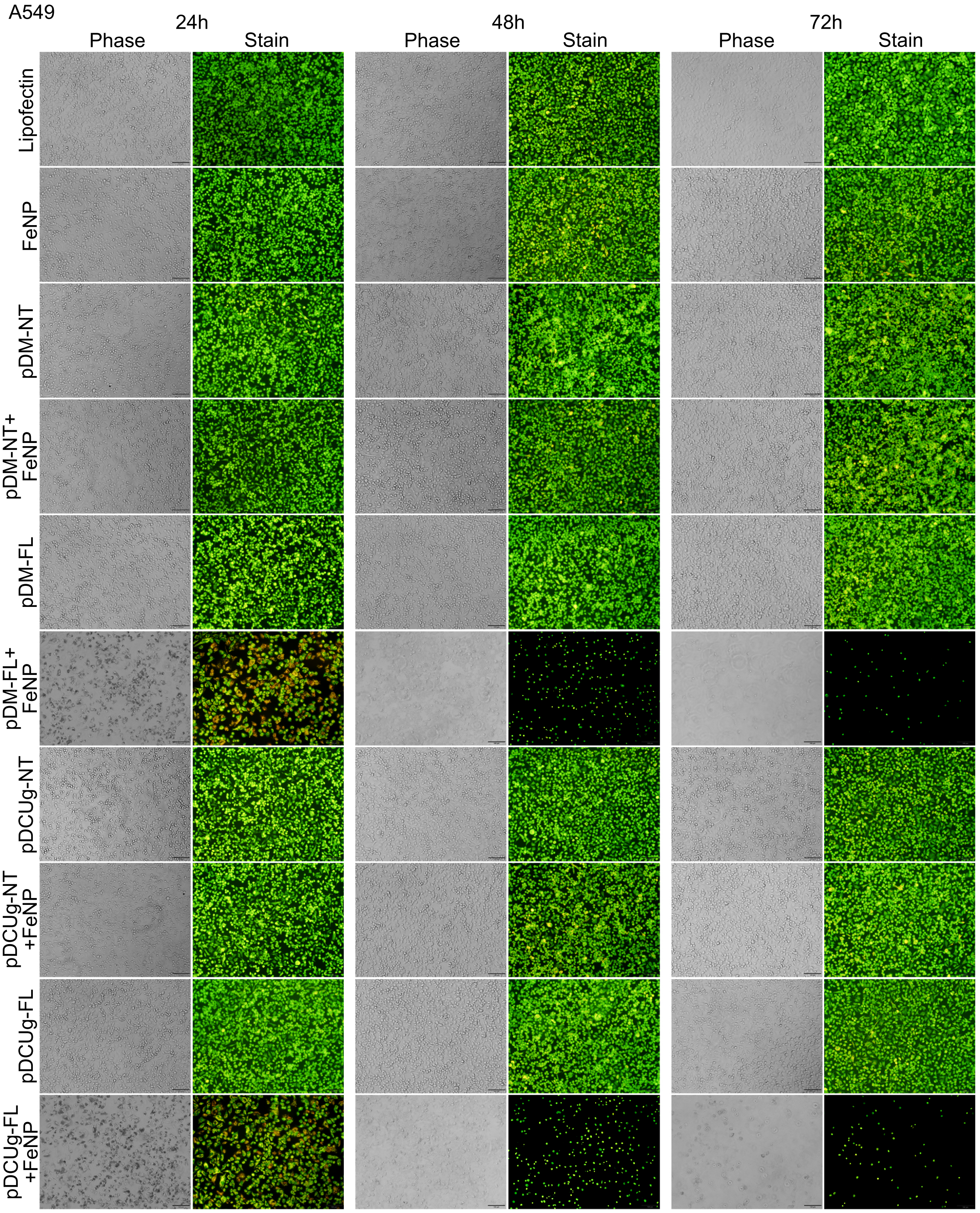

Supplementary Figure 6. Treatment of A549 cells with Gene interfering vectors and FeNP. Cells were transfected by various plasmids, cultured for 24 h and then incubated with or without 50 μg/mL of FeNP, and cells were cultured for another 72 h. At 24 h, 48 h and 72 h post FeNP administration, cells were stained with acridine orange and ethidium bromide and imaged under a fluorescence microscope. Scale bars, 100μm. Representative data of two independent experiments. pDM-NT/pDM-FL, plasmids expressing miRNAs under the control of DMP targeting no or *FPN&LCN2* transcripts; pDCUg-NT/pDCUg-FL, plasmids expressing Cas13a under the control of DMP and gRNAs under the control of U6 promoter targeting no or *FPN&LCN2* transcripts.

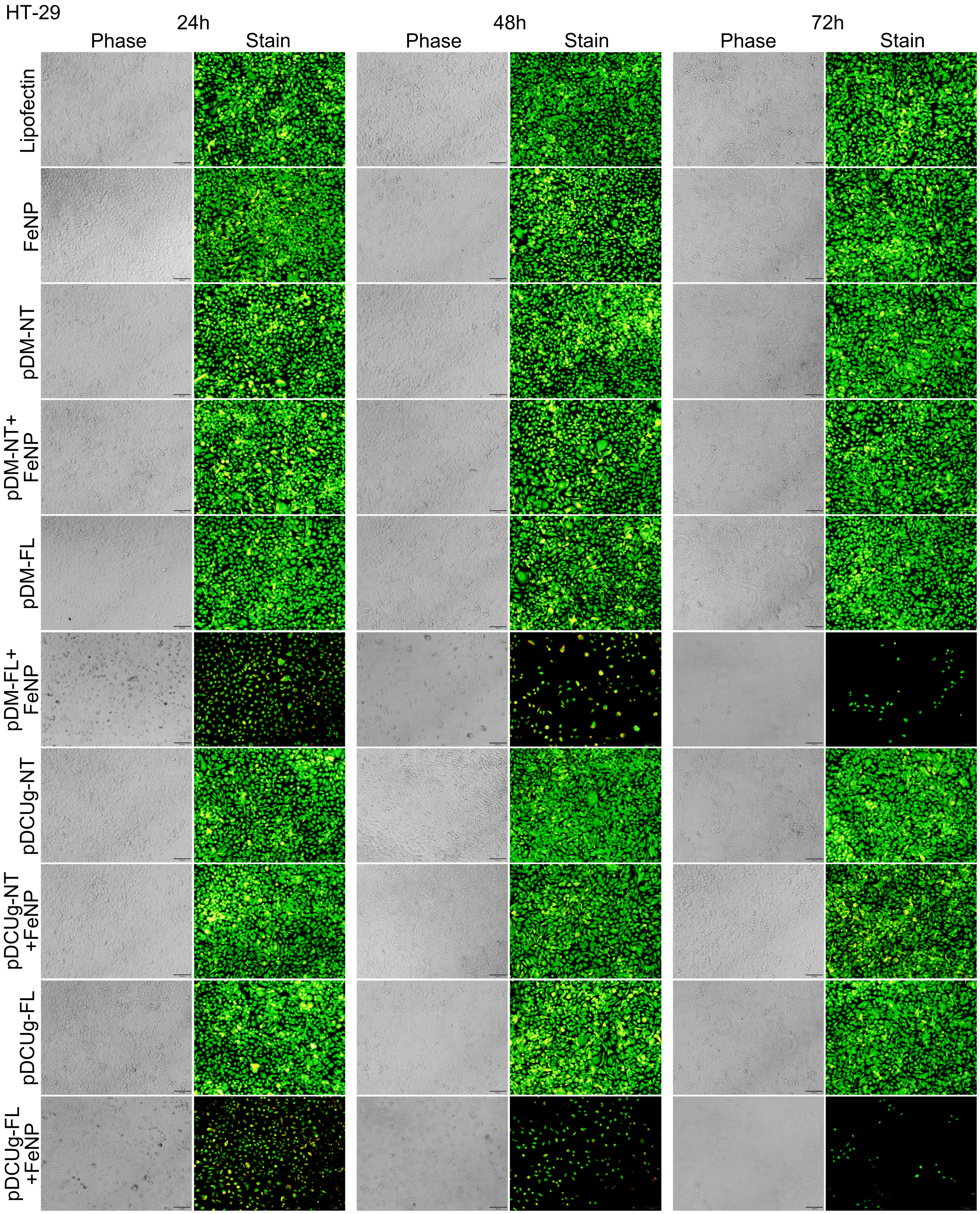

Supplementary Figure 7. Treatment of HT-29 cells with Gene interfering vectors and FeNP. Cells were transfected by various plasmids, cultured for 24 h and then incubated with or with-out 50 µg/mL of FeNP, and cells were cultured for another 72 h. At 24 h, 48 h and 72 h post FeNP administration, cells were stained with acridine orange and ethidium bromide and imaged under a fluorescence microscope. Scale bars, 100 µm. Representative data of two inde-pendent experiments.pDM-NT/pDM-FL, plasmids expressing miRNAs under the control of DMP targeting no or *FPN&LCN2* transcripts; pDCUg-NT/pDCUg-FL, plasmids expressing Cas13a under the control of DMP and gRNAs under the control of U6 promoter targeting no or *FPN&LCN2* transcripts.

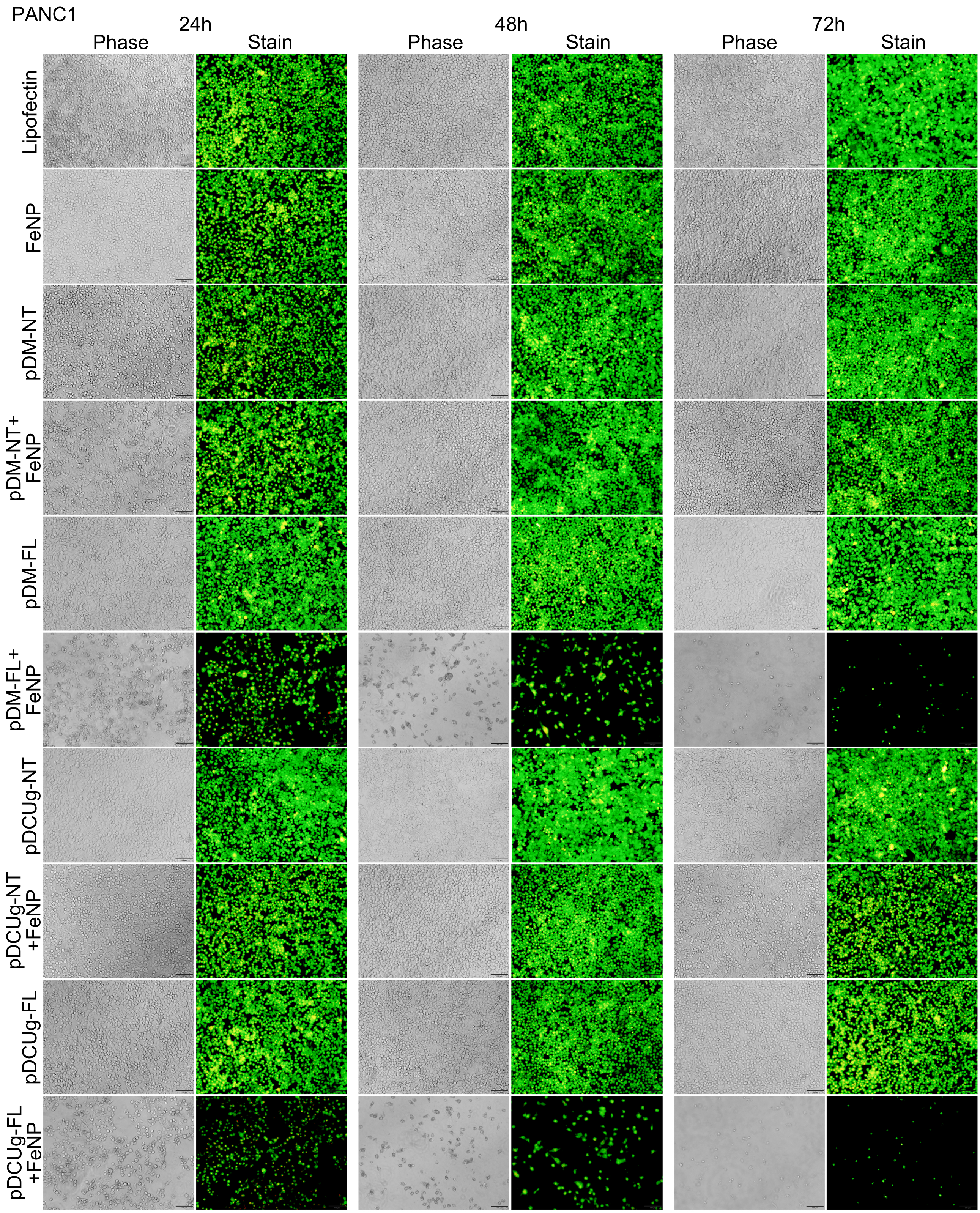

Supplementary Figure 8. Treatment of PANC1 cells with Gene interfering vectors and FeNP. Cells were transfected by various plasmids, cultured for 24 h and then incubated with or without 50 µg/mL of FeNP, and cells were cultured for another 72 h. At 24 h, 48 h and 72 h post FeNP administration, cells were stained with acridine orange and ethidium bromide and imaged under a fluorescence microscope. Scale bars, 100 µm. Representative data of two independent experi-ments. pDM-NT/pDM-FL, plasmids expressing miRNAs under the control of DMP targeting no or *FPN&LCN2* transcripts; pDCUg-NT/pDCUg-FL, plasmids expressing Cas13a under the control of DMP and gRNAs under the control of U6 promoter targeting no or *FPN&LCN2* tran-scripts.

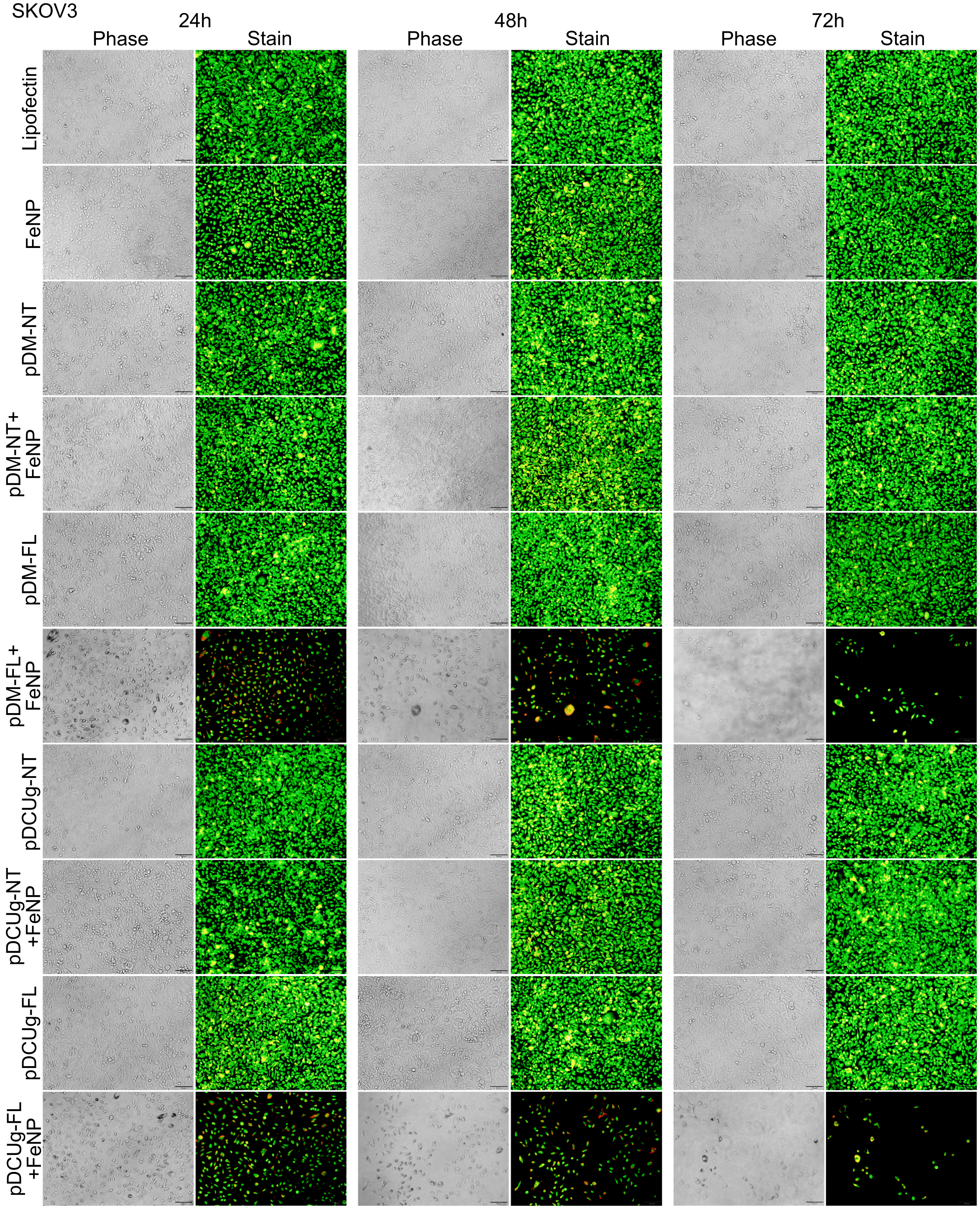

Supplementary Figure 9. Treatment of SKOV3 cells with Gene interfering vectors and FeNP. Cells were transfected by various plasmids, cultured for 24 h and then incubated with or without 50 µg/mL of FeNP, and cells were cultured for another 72 h. At 24 h, 48 h and 72 h post FeNP administration, cells were stained with acridine orange and ethidium bromide and imaged under a fluorescence microscope. Scale bars, 100 µm. Representative data of two independent experiments. pDM-NT/pDM-FL, plasmids expressing miRNAs under the control of DMP targeting no or *FPN&LCN2* transcripts; pDCUg-NT/pDCUg-FL, plasmids expressing Cas13a under the control of DMP and gRNAs under the control of U6 promoter targeting no or *FPN&LCN2* transcripts.

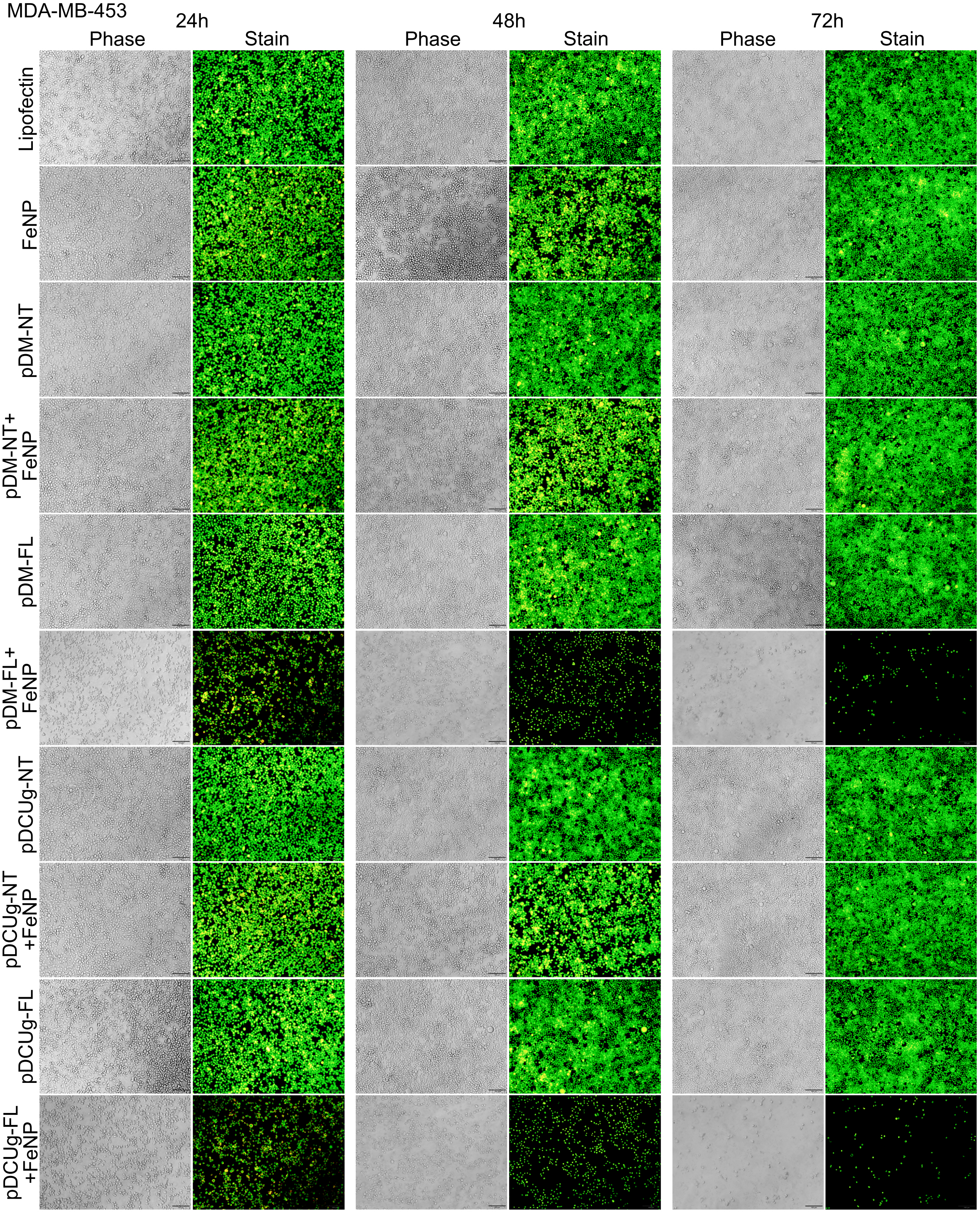

Supplementary Figure 10. Treatment of MDA-MB-453 cells with gene interfering vectors and FeNP. Cells were transfected by various plasmids, cultured for 24 h and then incubated with or without 50 μg/mL of FeNP, and cells were cultured for another 72 h. At 24 h, 48 h and 72 h post FeNP administration, cells were stained with acridine orange and ethidium bromide and imaged under a fluorescence microscope. Scale bars, 100 μm. Representative data of two independent experiments. pDM-NT/pDM-FL, plasmids expressing miRNAs under the control of DMP targeting no or *FPN&LCN2* transcripts; pDCUg-NT/pDCUg-FL, plasmids expressing Cas13a under the control of DMP and gRNAs under the control of U6 promoter targeting no or *FPN&LCN2* transcripts.

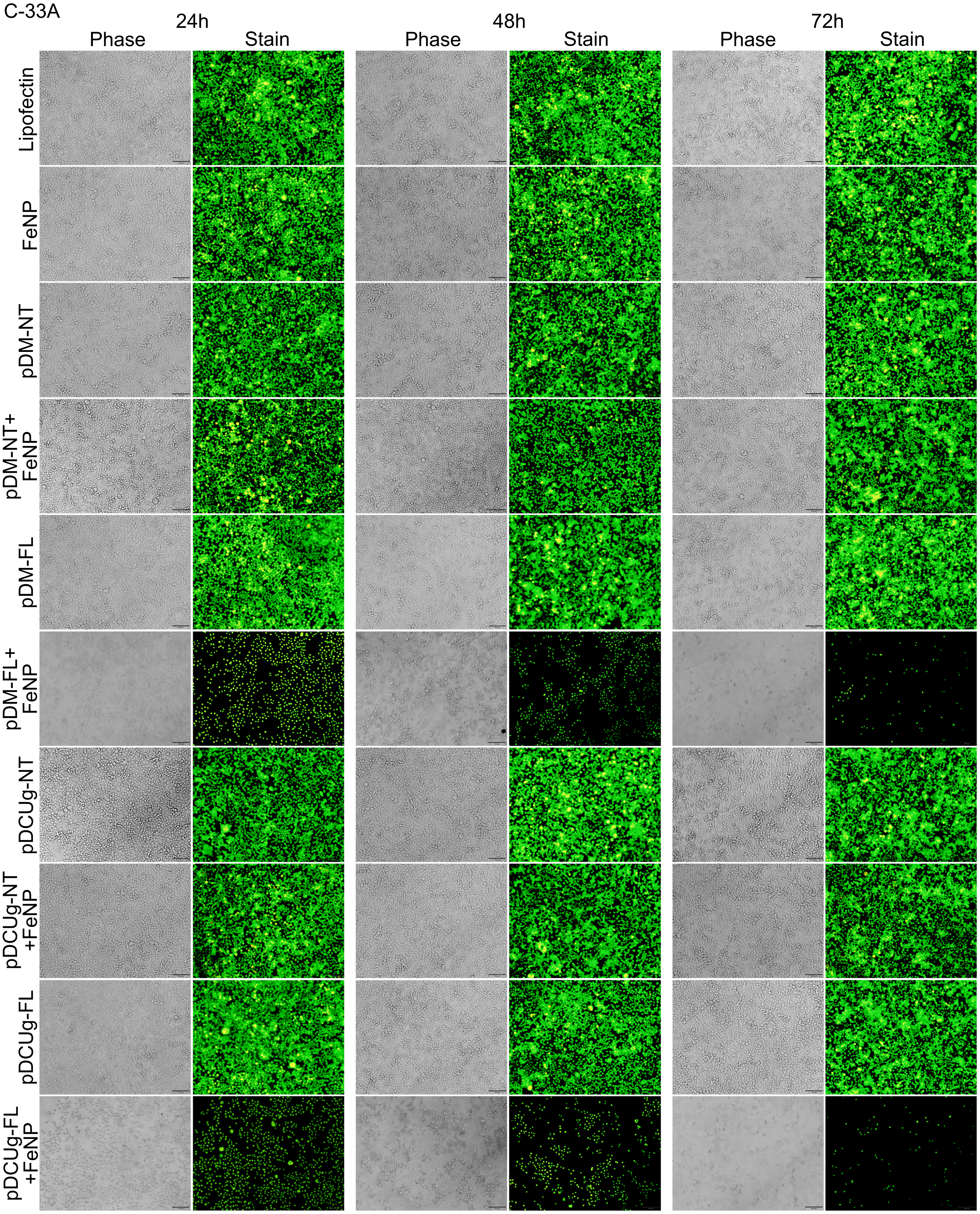

Supplementary Figure 11. Treatment of C-33A cells with Gene interfering vectors and FeNP. Cells were transfected by various plasmids, cultured for 24 h and then incubated with or with-out 50 µg/mL of FeNP, and cells were cultured for another 72 h. At 24 h, 48 h and 72 h post FeNP administration, cells were stained with acridine orange and ethidium bromide and imaged under a fluorescence microscope. Scale bars, 100 µm. Representative data of two independent experiments. pDM-NT/pDM-FL, plasmids expressing miRNAs under the control of DMP targeting no or *FPN&LCN2* transcripts; pDCUg-NT/pDCUg-FL, plasmids expressing Cas13a under the control of DMP and gRNAs under the control of U6 promoter targeting no or *FPN&LCN2* transcripts.

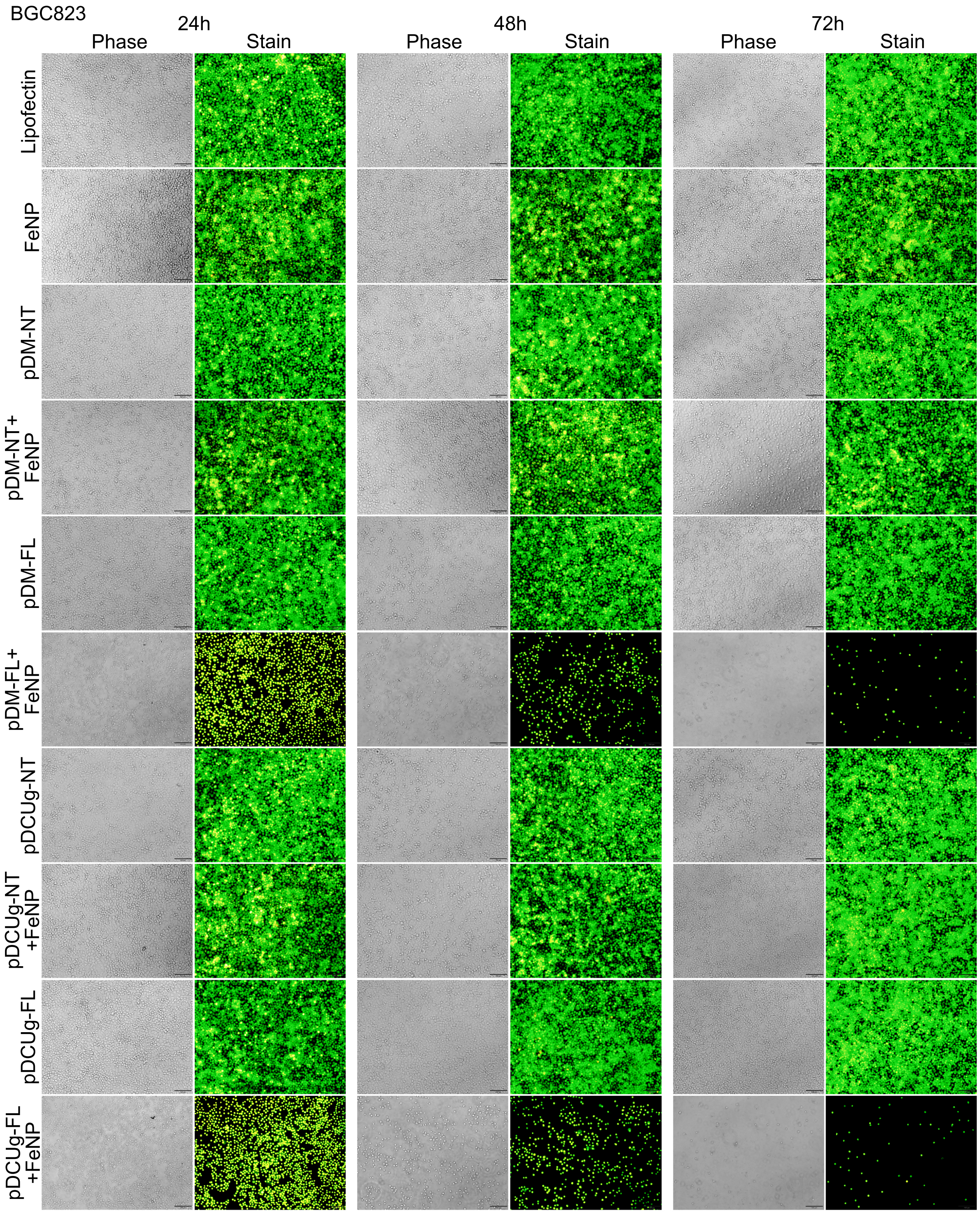

Supplementary Figure 12. Treatment of BGC823 cells with Gene interfering vectors and FeNP. Cells were transfected by various plasmids, cultured for 24 h and then incubated with or without 50 μg/mL of FeNP, and cells were cultured for another 72 h. At 24 h, 48 h and 72 h post FeNP administration, cells were stained with acridine orange and ethidium bromide and imaged under a fluorescence microscope. Scale bars, 100 μm. Representative data of two independent experiments. pDM-NT/pDM-FL, plasmids expressing miRNAs under the control of DMP targeting no or *FPN&LCN2* transcripts; pDCUg-NT/pDCUg-FL, plasmids expressing Cas13a under the control of DMP and gRNAs under the control of U6 promoter targeting no or *FPN&LCN2* transcripts.

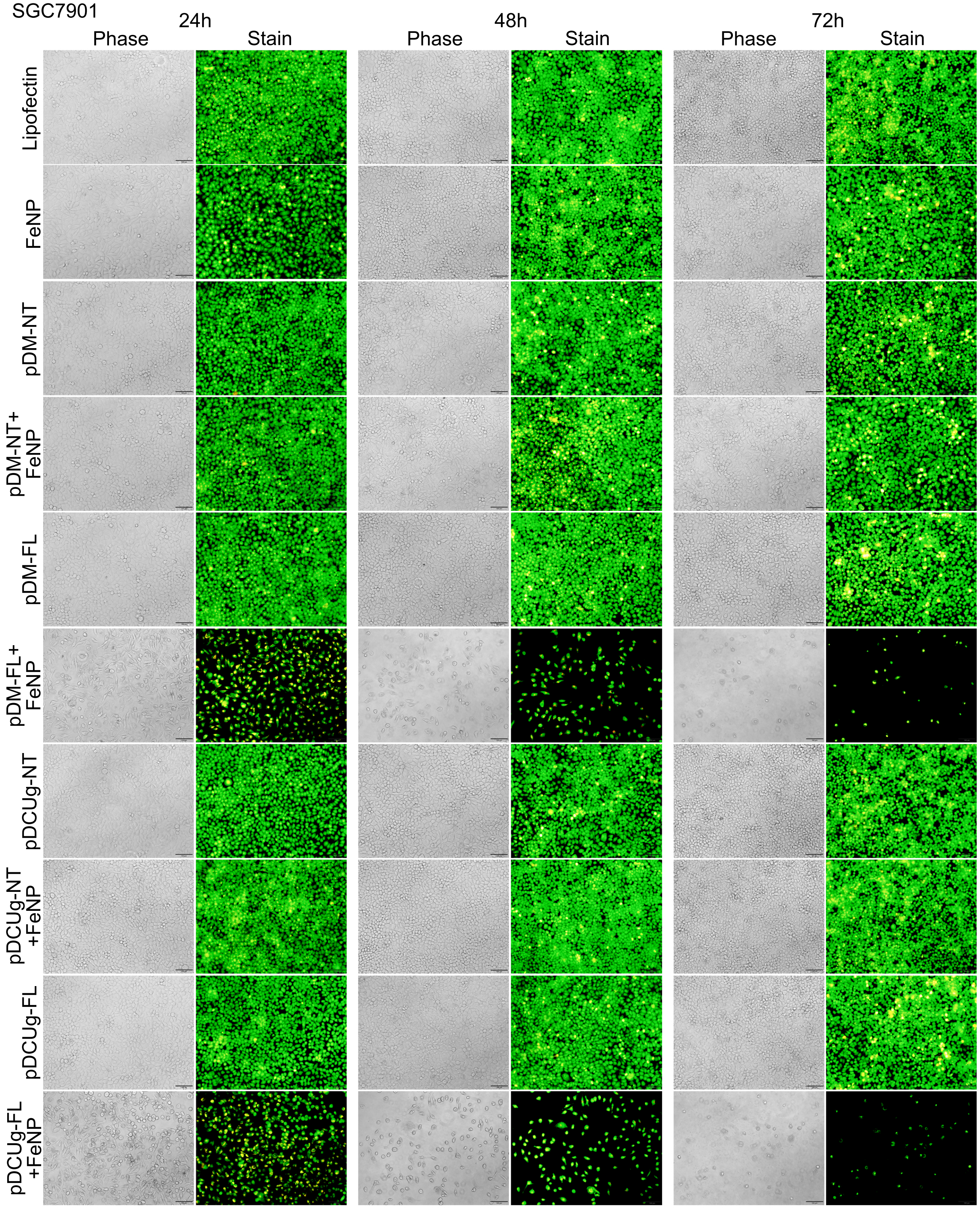

Supplementary Figure 13. Treatment of SGC7901 cells with Gene interfering vectors and FeNP. Cells were transfected by various plasmids, cultured for 24 h and then incubated with or without 50 μg/mL of FeNP, and cells were cultured for another 72 h. At 24 h, 48 h and 72 h post FeNP administration, cells were stained with acridine orange and ethidium bromide and imaged under a fluorescence microscope. Scale bars, 100 μm. Representative data of two independent experiments. pDM-NT/pDM-FL, plasmids expressing miRNAs under the control of DMP targeting no or *FPN&LCN2* transcripts; pDCUg-NT/pDCUg-FL, plasmids expressing Cas13a under the control of DMP and gRNAs under the control of U6 promoter targeting no or *FPN&LCN2* transcripts.

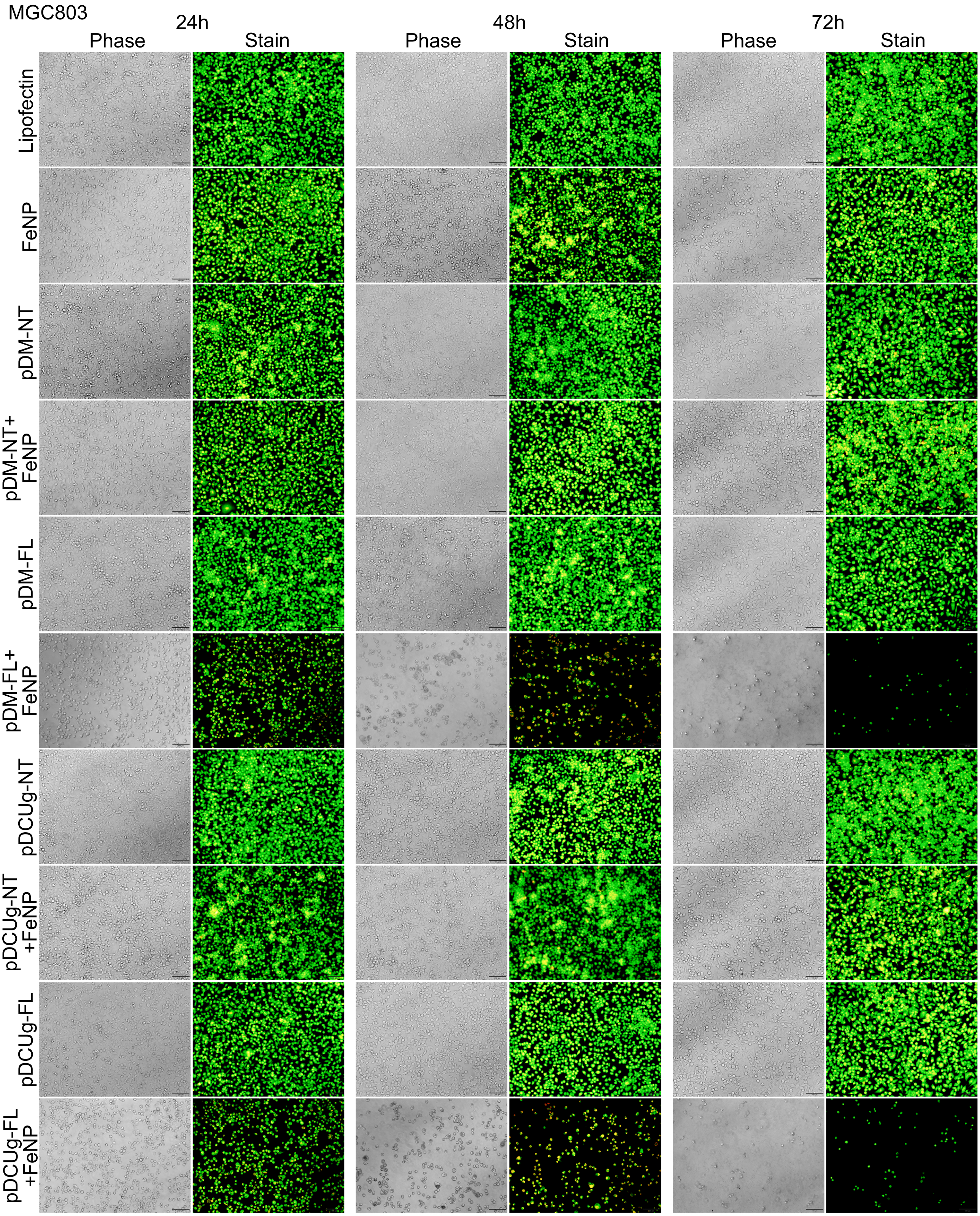

Supplementary Figure 14. Treatment of MGC803 cells with Gene interfering vectors and FeNP. Cells were transfected by various plasmids, cultured for 24 h and then incubated with or without 50 μg/mL of FeNP, and cells were cultured for another 72 h. At 24 h, 48 h and 72 h post FeNP administration, cells were stained with acridine orange and ethidium bromide and imaged under a fluorescence microscope. Scale bars, 100 μm. Representative data of two independent experi-ments. pDM-NT/pDM-FL, plasmids expressing miRNAs under the control of DMP targeting no or *FPN&LCN2* transcripts; pDCUg-NT/pDCUg-FL, plasmids expressing Cas13a under the control of DMP and gRNAs under the control of U6 promoter targeting no or *FPN&LCN2* tran-scripts.

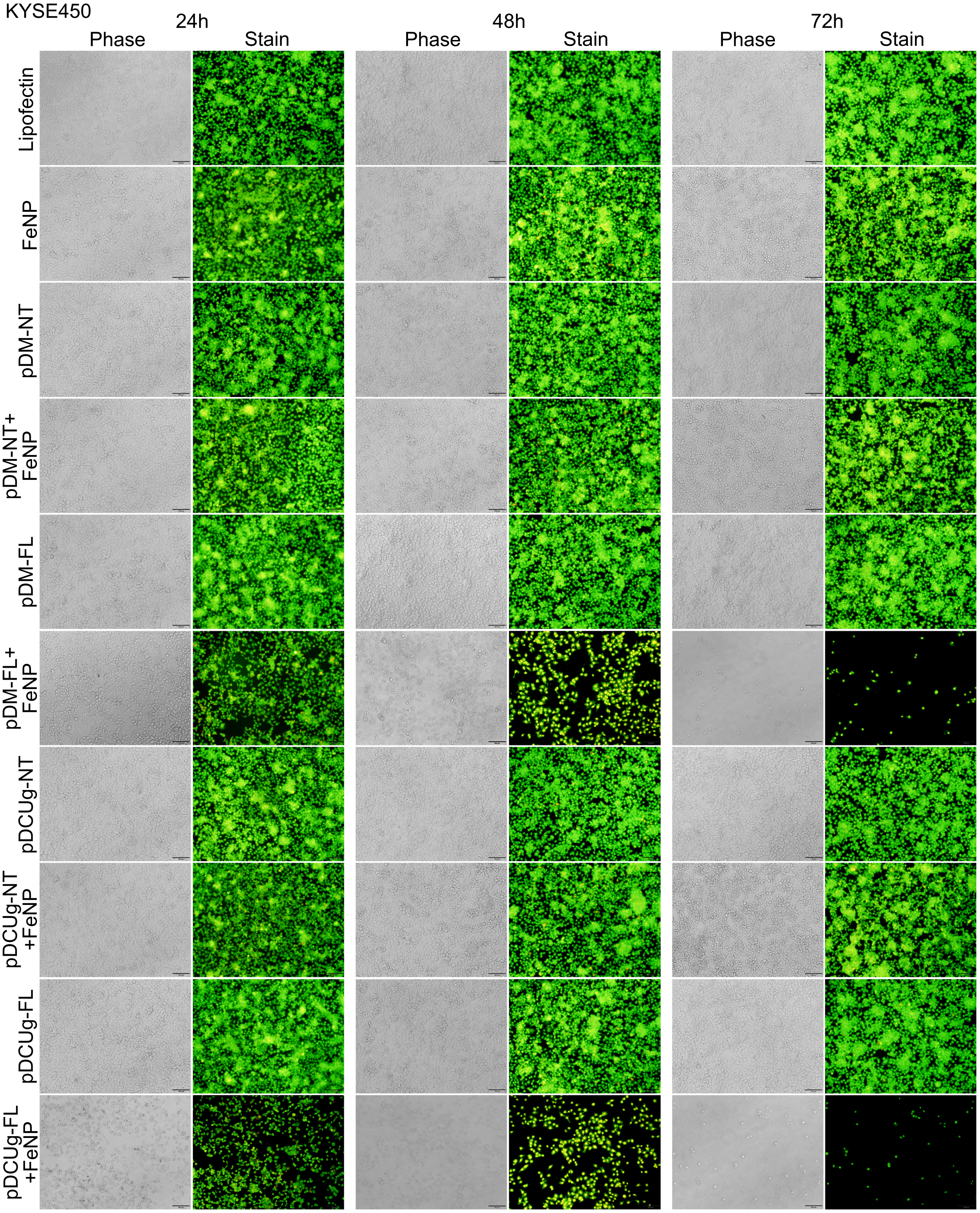

Supplementary Figure 15. Treatment of KYSE450 cells with Gene interfering vectors and FeNP. Cells were transfected by various plasmids, cultured for 24 h and then incubated with or without 50 μg/mL of FeNP, and cells were cultured for another 72 h. At 24 h, 48 h and 72 h post FeNP administration, cells were stained with acridine orange and ethidium bromide and imaged under a fluorescence microscope. Scale bars, 100 μm. Representative data of two independent experi-ments. pDM-NT/pDM-FL, plasmids expressing miRNAs under the control of DMP targeting no or *FPN&LCN2* transcripts; pDCUg-NT/pDCUg-FL, plasmids expressing Cas13a under the control of DMP and gRNAs under the control of U6 promoter targeting no or *FPN&LCN2* tran-scripts.

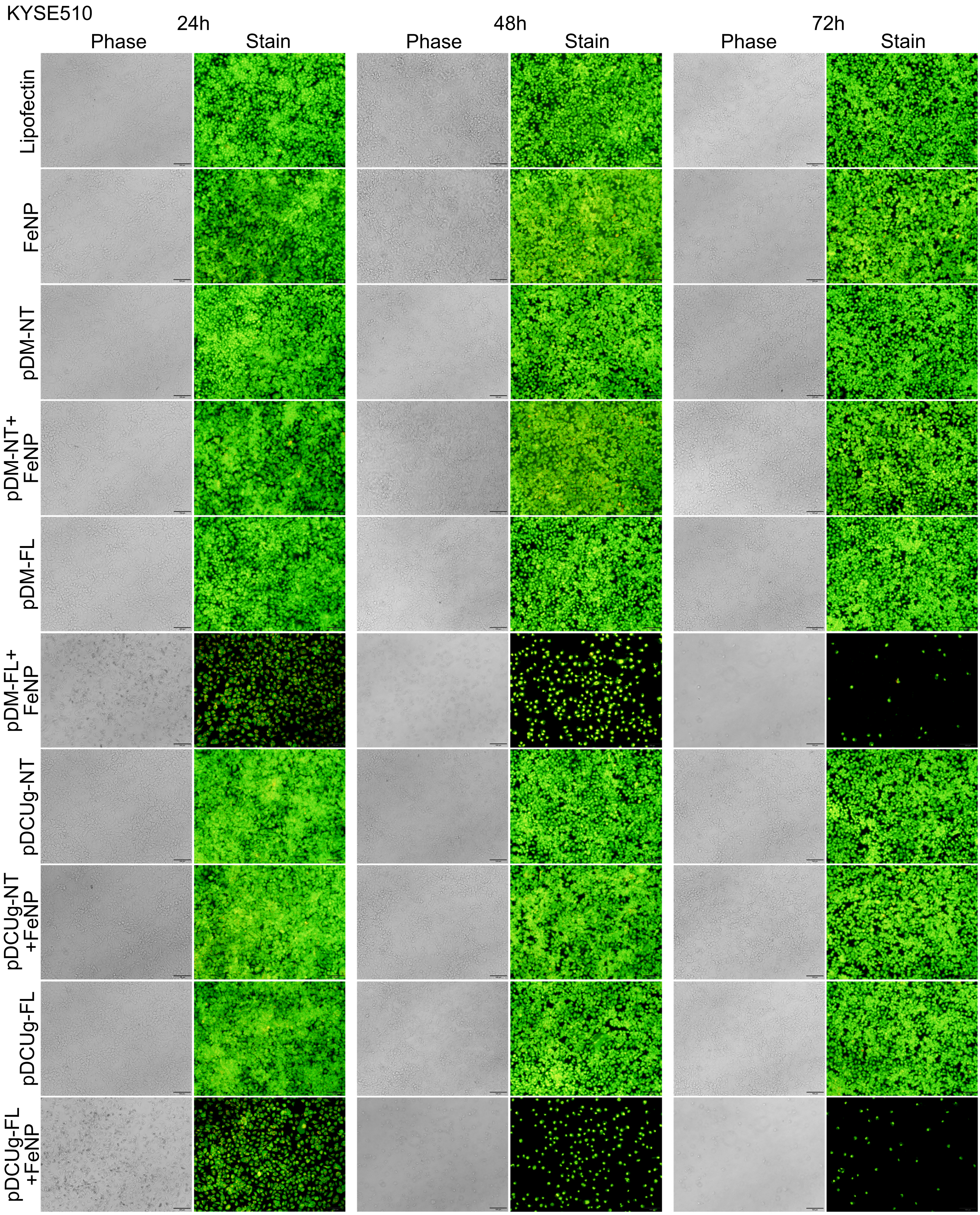

Supplementary Figure 16. Treatment of KYSE510 cells with Gene interfering vectors and FeNP. Cells were transfected by various plasmids, cultured for 24 h and then incubated with or without 50 μg/mL of FeNP, and cells were cultured for another 72 h. At 24 h, 48 h and 72 h post FeNP administration, cells were stained with acridine orange and ethidium bromide and imaged under a fluorescence microscope.Scale bars, 100 μm. Representative data of two independent experiments. pDM-NT/pDM-FL, plasmids expressing miRNAs under the control of DMP targeting no or *FPN&LCN2* transcripts; pDCUg-NT/pDCUg-FL, plasmids expressing Cas13a under the control of DMP and gRNAs under the control of U6 promoter targeting no or *FPN&LCN2* transcripts.

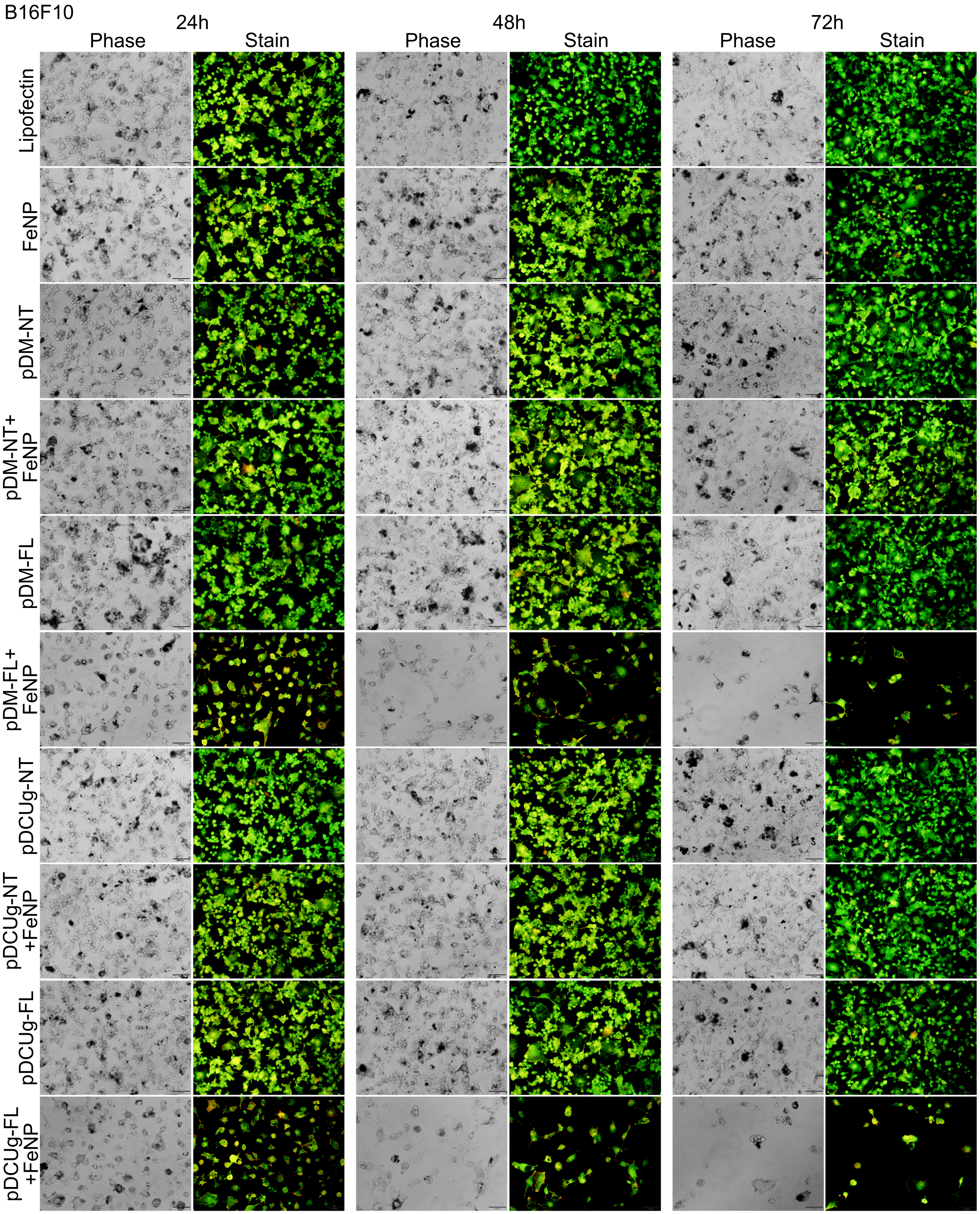

Supplementary Figure 17. Treatment of B16F10 cells with Gene interfering vectors and FeNP. Cells were transfected by various plasmids, cultured for 24 h and then incubated with or without 50 μg/mL of FeNP, and cells were cultured for another 72 h. At 24 h, 48 h and 72 h post FeNP administration, cells were stained with acridine orange and ethidium bromide and imaged under a fluorescence microscope. Scale bars, 100 μm. Representative data of two independent experi-ments. pDM-NT/pDM-FL, plasmids expressing miRNAs under the control of DMP targeting no or *FPN&LCN2* transcripts; pDCUg-NT/pDCUg-FL, plasmids expressing Cas13a under the control of DMP and gRNAs under the control of U6 promoter targeting no or mouse *FPN&LCN2* transcripts.

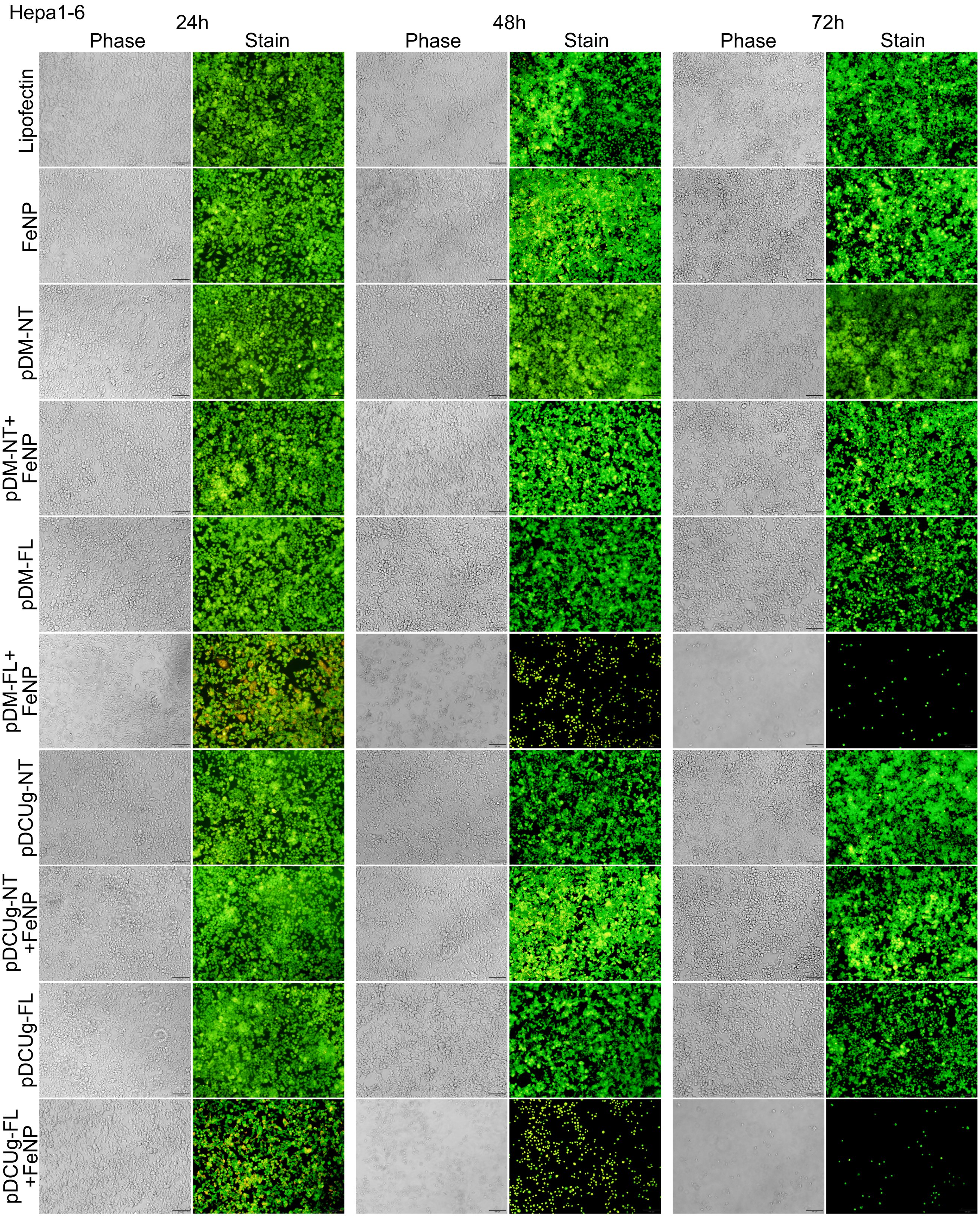

Supplementary Figure 18. Treatment of Hepa1-6 cells with Gene interfering vectors and FeNP. Cells were transfected by various plasmids, cultured for 24 h and then incubated with or without 50 μg/mL of FeNP, and cells were cultured for another 72 h. At 24 h, 48 h and 72 h post FeNP adminis-tration, cells were stained with acridine orange and ethidium bromide and imaged under a fluores-cence microscope. Scale bars, 100 μm. Representative data of two independent experiments. pDM-NT/pDM-FL, plasmids expressing miRNAs under the control of DMP targeting no or *FPN&LCN2* transcripts; pDCUg-NT/pDCUg-FL, plasmids expressing Cas13a under the control of DMP and gRNAs under the control of U6 promoter targeting no or mouse *FPN&LCN2* transcripts.

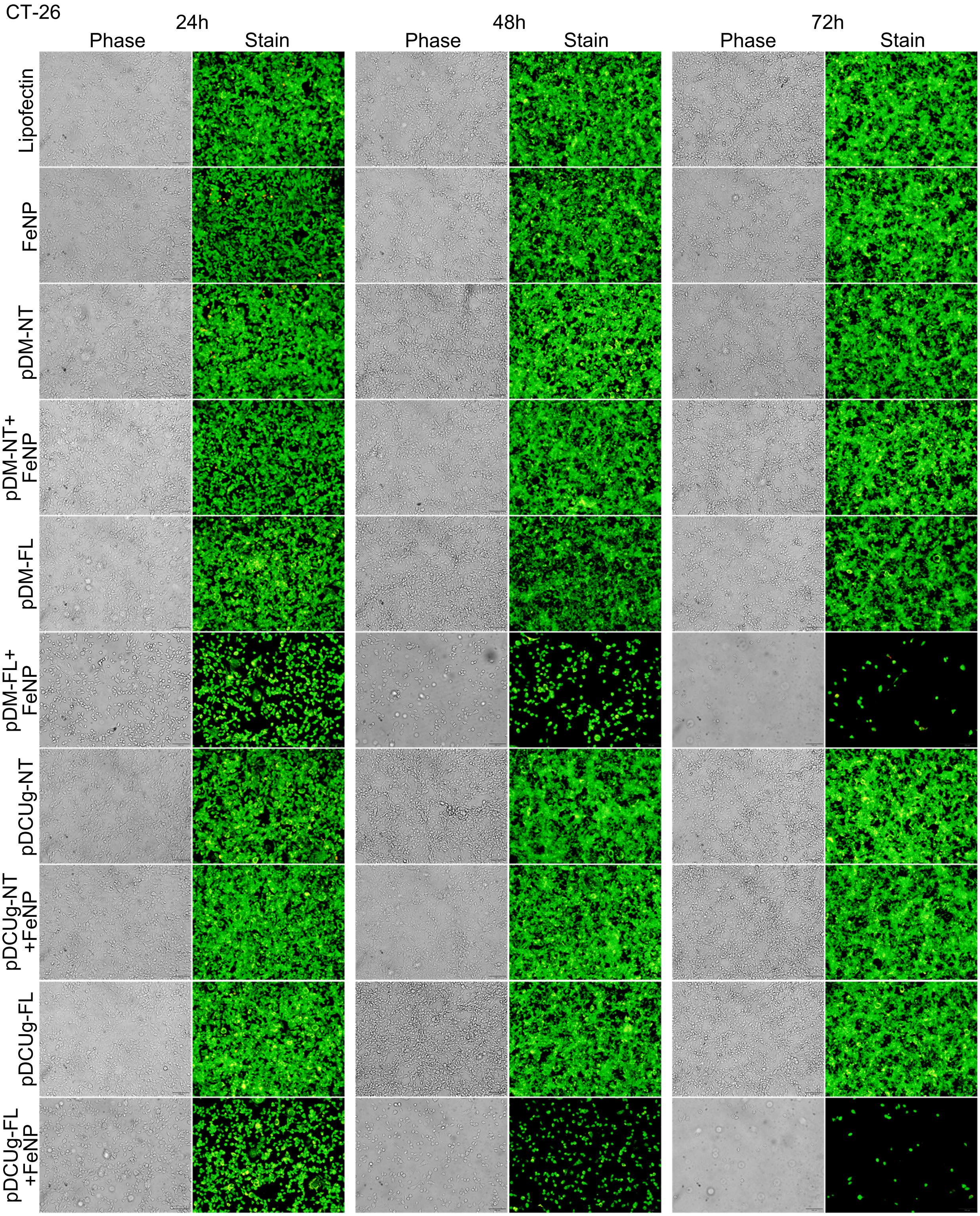

Supplementary Figure 19. Treatment of CT-26 cells with Gene interfering vectors and FeNP. Cells were transfected by various plasmids, cultured for 24 h and then incubated with or without 50 μg/mL of FeNP, and cells were cultured for another 72 h. At 24 h, 48 h and 72 h post FeNP administration, cells were stained with acridine orange and ethidium bromide and imaged under a fluorescence microscope. Scale bars, 100 μm. Representative data of two independent experiments. pDM-NT/pDM-FL, plasmids expressing miRNAs under the control of DMP targeting no or *FPN&LCN2* transcripts; pDCUg-NT/pDCUg-FL, plasmids expressing Cas13a under the control of DMP and gRNAs under the control of U6 promoter targeting no or mouse *FPN&LCN2* transcripts.

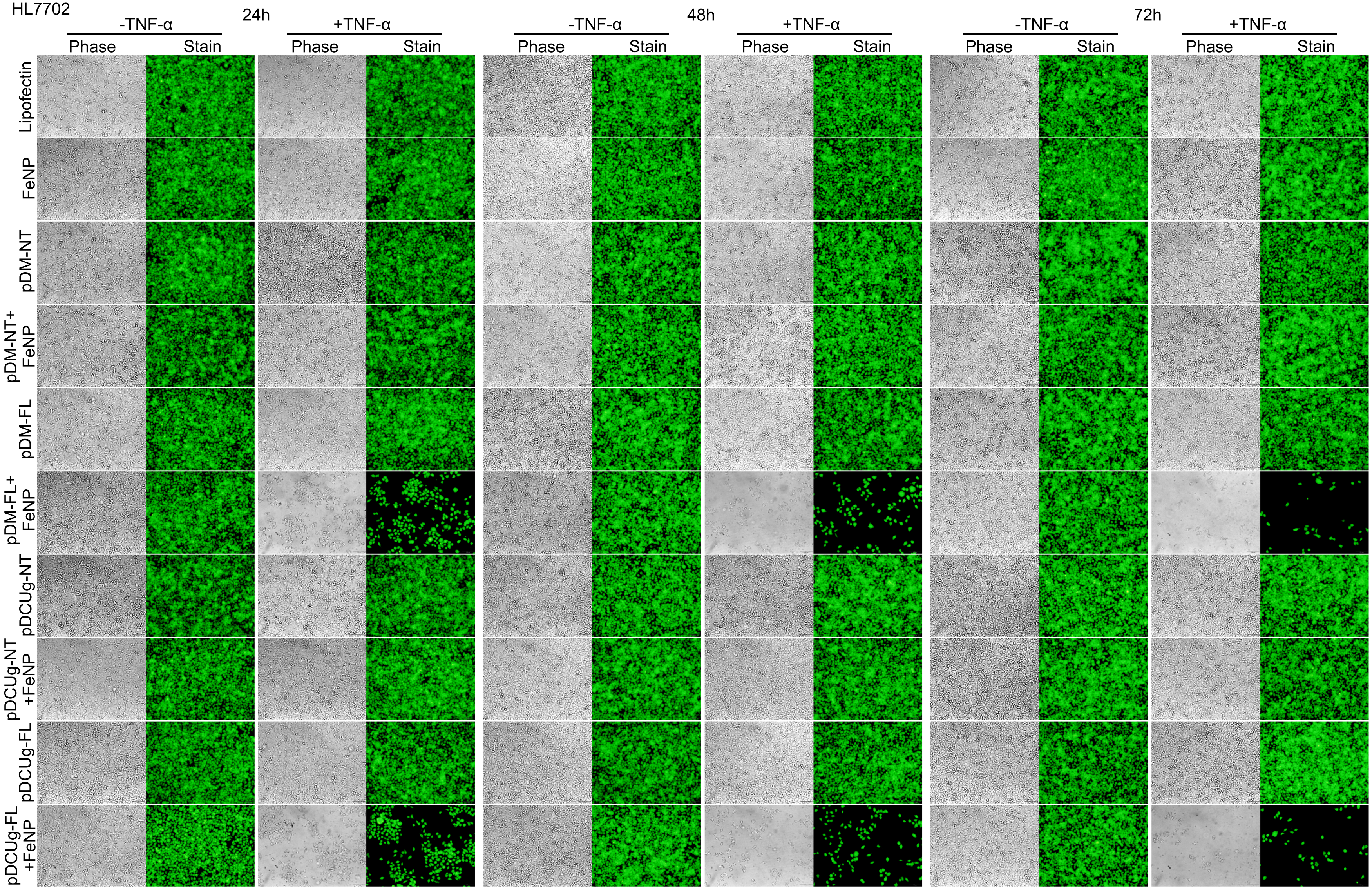

Supplementary Figure 20. Treatment of HL7702 cells with Gene interfering vectors and FeNP. Cells were transfected by various plasmids, cultured for 24 h and then induced with or without TNFα (10 ng/mL) for 1 h before incubating with or without 50 µg/mL of FeNP, and cells were cultured for another 72 h. At 24 h, 48 h and 72 h post FeNP administration, cells were stained with acridine orange and ethidium bromide and imaged under a fluorescence microscope. Scale bars, 100 µm. Representative data of two independent experiments. pDM-NT/pDM-FL, plasmids expressing miRNAs under the control of DMP targeting no or *FPN&LCN2* transcripts; pDCUg-NT/pDCUg-FL, plasmids expressing Cas13a under the control of DMP and gRNAs under the control of U6 promoter targeting no or *FPN&LCN2* transcripts.

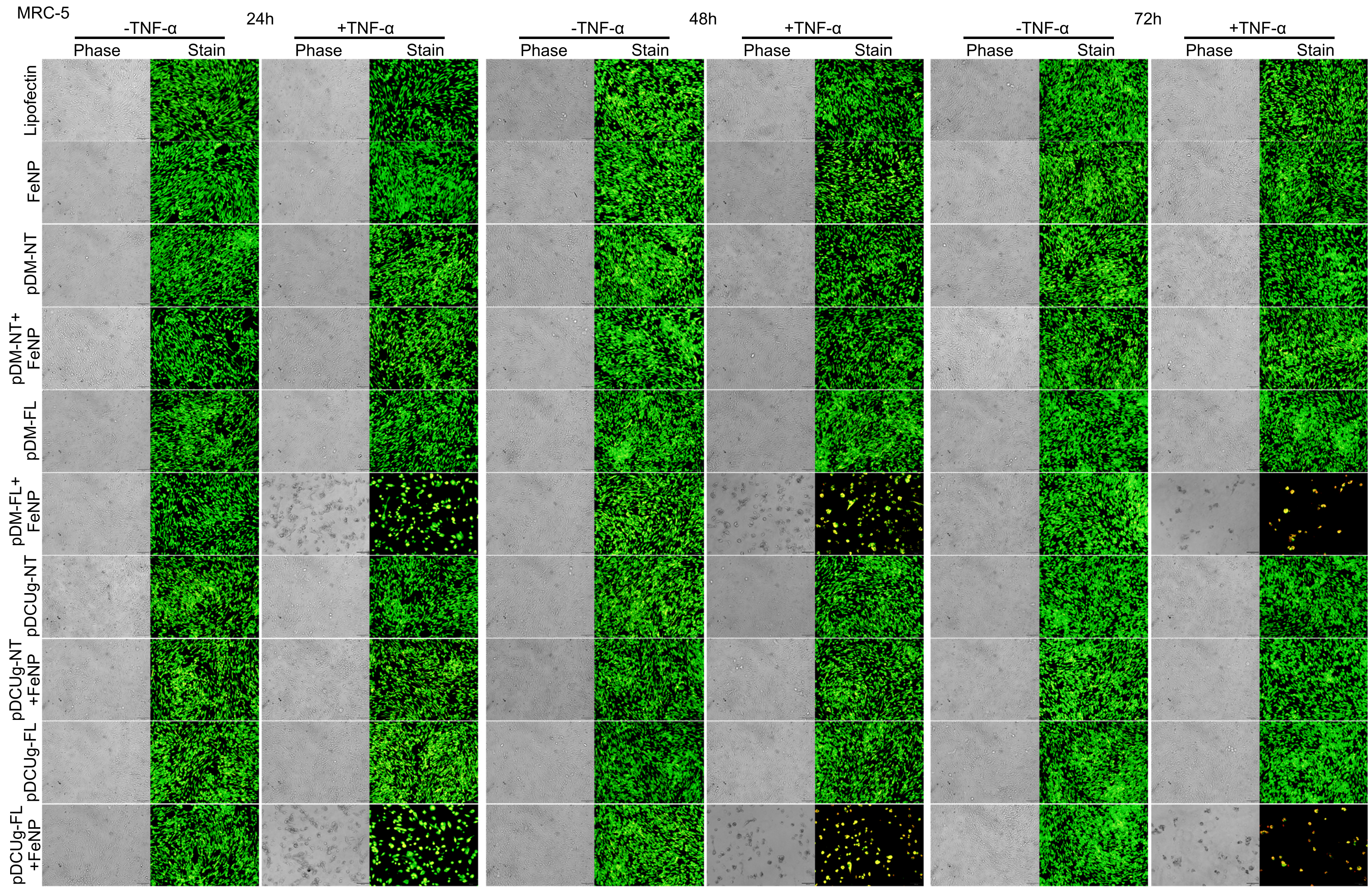

Supplementary Figure 21. Treatment of MRC-5 cells with Gene interfering vectors and FeNP. Cells were transfected by various plasmids, cultured for 24 h and then induced with or without TNFα (10 ng/mL) for 1 h before incubating with or without 50 µg/mL of FeNP, and cells were cultured for another 72 h. At 24 h, 48 h and 72 h post FeNP administration, cells were stained with acridine orange and ethidium bromide and imaged under a fluorescence microscope. Scale bars, 100 µm. Representative data of two independent experiments. pDM-NT/pDM-FL, plasmids expressing miRNAs under the control of DMP targeting no or *FPN&LCN2* transcripts; pDCUg-NT/pDCUg-FL, plasmids express-ing Cas13a under the control of DMP and gRNAs under the control of U6 promoter targeting no or *FPN&LCN2* transcripts.

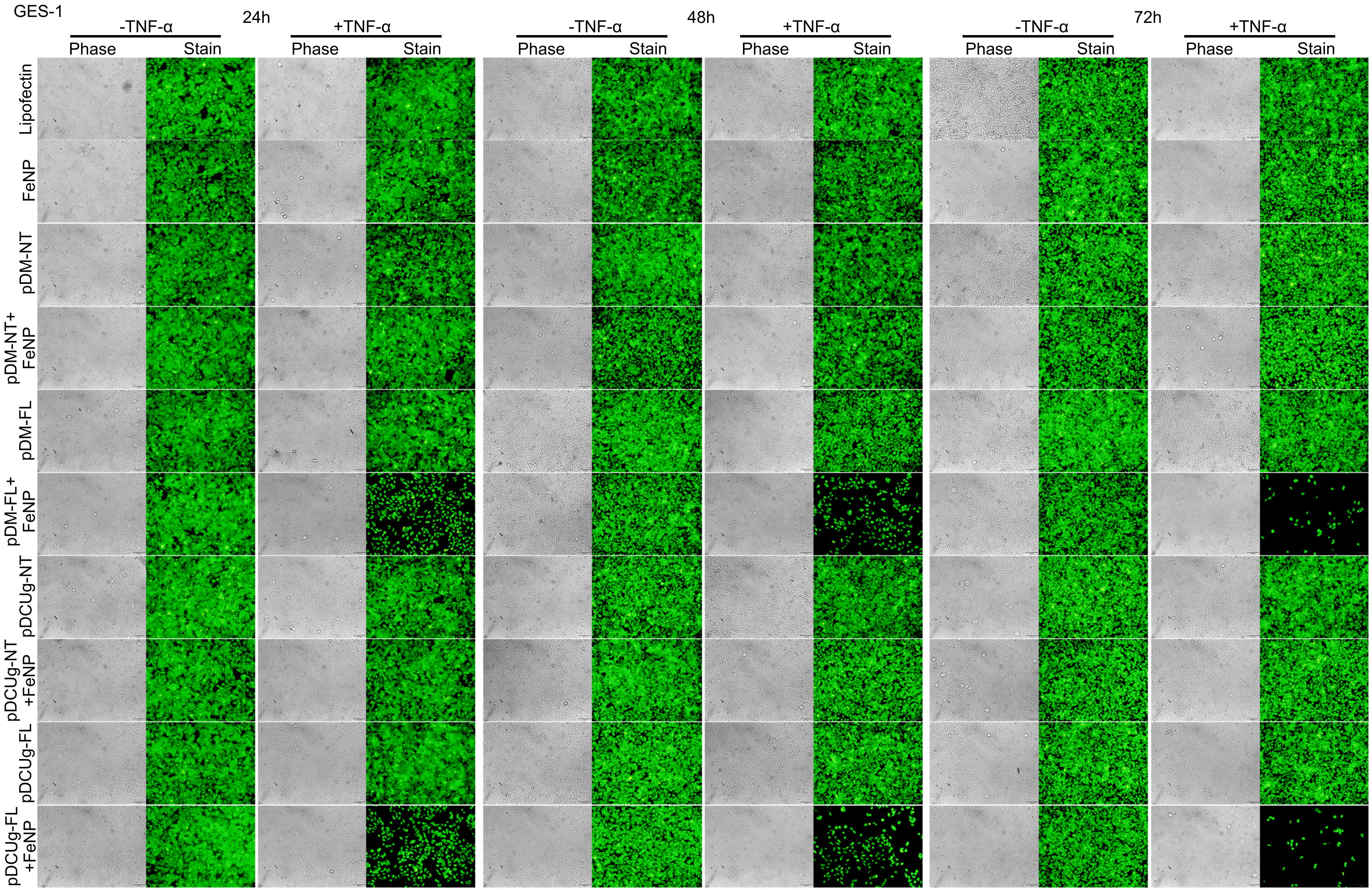

Supplementary Figure 22. Treatment of GES-1 cells with Gene interfering vectors and FeNP. Cells were transfected by various plasmids, cultured for 24 h and then induced with or without TNFα (10 ng/mL) for 1 h before incubating with or without 50 µg/mL of FeNP, and cells were cultured for another 72 h. At 24 h, 48 h and 72 h post FeNP administration, cells were stained with acridine orange and ethidium bromide and imaged under a fluorescence microscope. Scale bars, 100 µm. Representative data of two independent experiments. pDM-NT/pDM-FL, plasmids expressing miRNAs under the control of DMP targeting no or *FPN&LCN2* transcripts; pDCUg-NT/pDCUg-FL, plasmids expressing Cas13a under the control of DMP and gRNAs under the control of U6 promoter targeting no or *FPN&LCN2* transcripts.

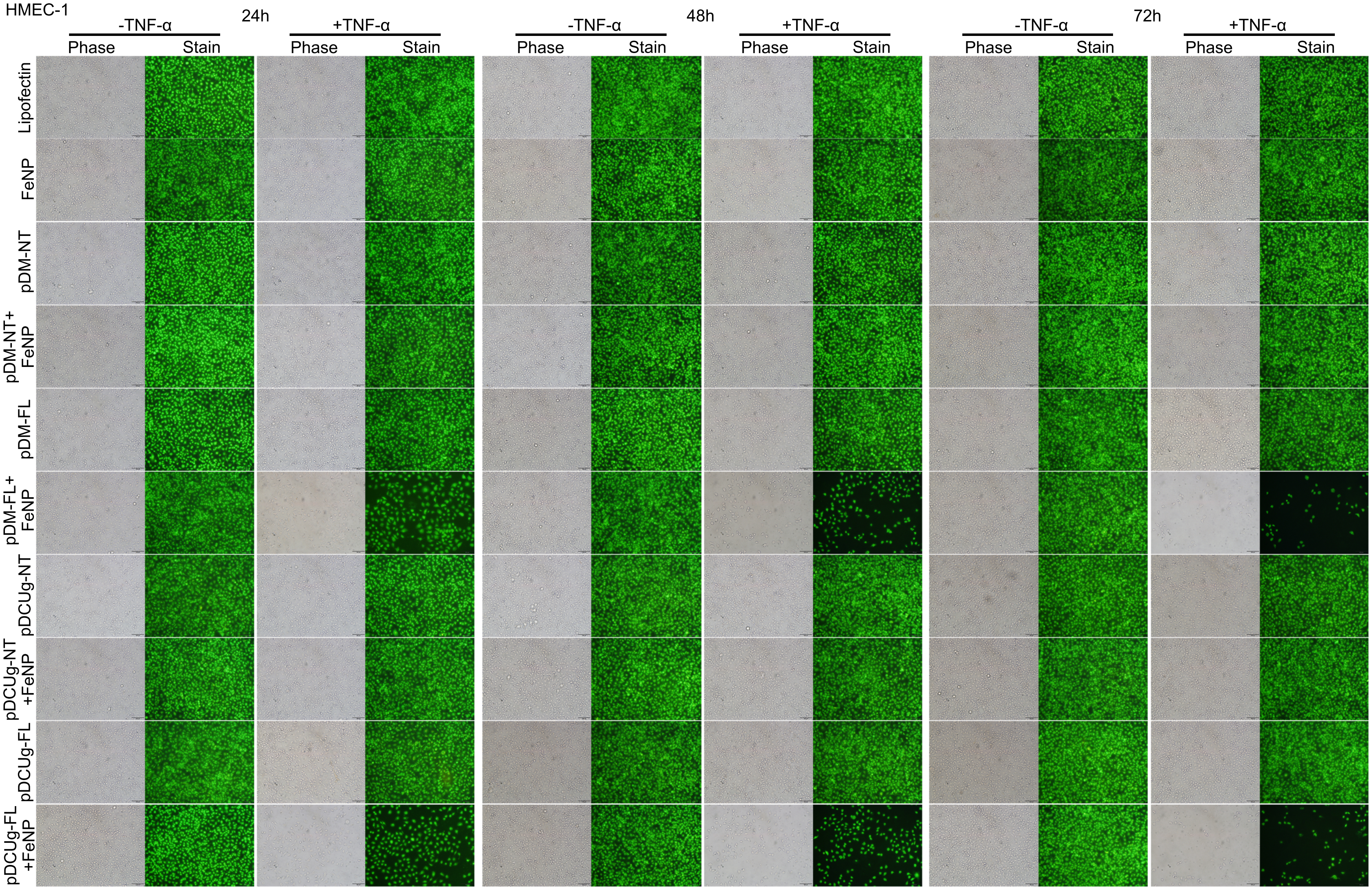

Supplementary Figure 23. Treatment of HMEC-1 cells with Gene interfering vectors and FeNP. Cells were transfected by various plasmids, cultured for 24 h and then induced with or without TNFα (10 ng/mL) for 1 h before incubating with or without 50 μg/mL of FeNP, and cells were cultured for another 72 h. At 24 h, 48 h and 72 h post FeNP administration, cells were stained with acridine orange and ethidium bromide and imaged under a fluorescence microscope. Scale bars, 100 μm. Representative data of two independent experiments. pDM-NT/ pDM-FL, plasmids expressing miRNAs under the control of DMP targeting no or *FPN&LCN2* transcripts; pDCUg-NT/pDCUg-FL, plasmids expressing Cas13a under the control of DMP and gRNAs under the control of U6 promoter targeting no or *FPN&LCN2* transcripts.

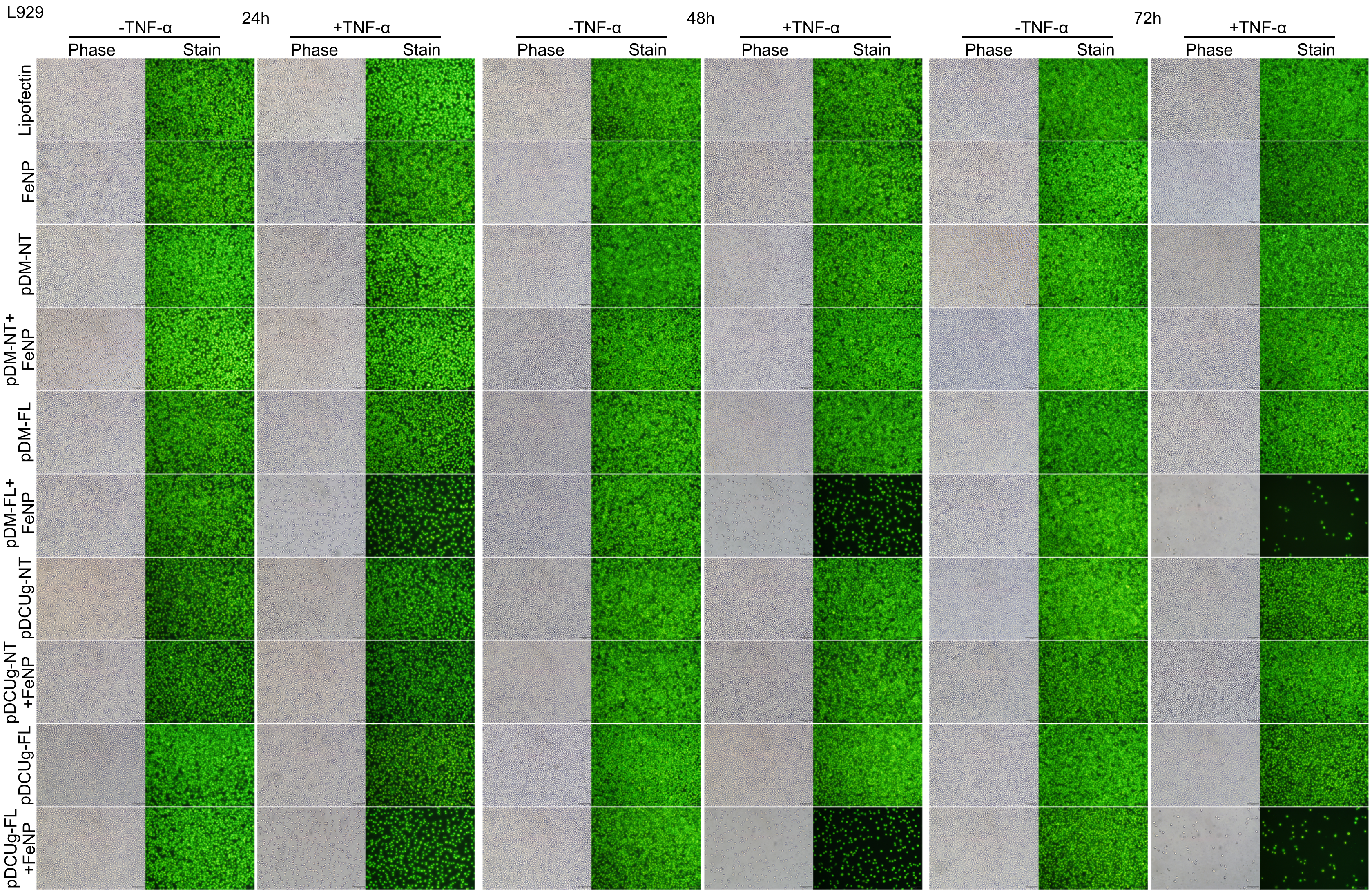

Supplementary Figure 24. Treatment of L929 cells with Gene interfering vectors and FeNP. Cells were transfected by various plasmids, cultured for 24 h and then induced with or without TNFα (10 ng/mL) for 1 h before incubating with or without 50 µg/mL of FeNP, and cells were cultured for another 72 h. At 24 h, 48 h and 72 h post FeNP administration, cells were stained with acridine orange and ethidium bromide and imaged under a fluorescence microscope. Scale bars, 100 µm. Representative data of two independent experiments.pDM-NT/pDM-FL, plasmids expressing miRNAs under the control of DMP targeting no or *FPN&LCN2* transcripts; pDCUg-NT/pDCUg-FL, plasmids expressing Cas13a under the control of DMP and gRNAs under the control of U6 promoter targeting no or *FPN&LCN2* transcripts.

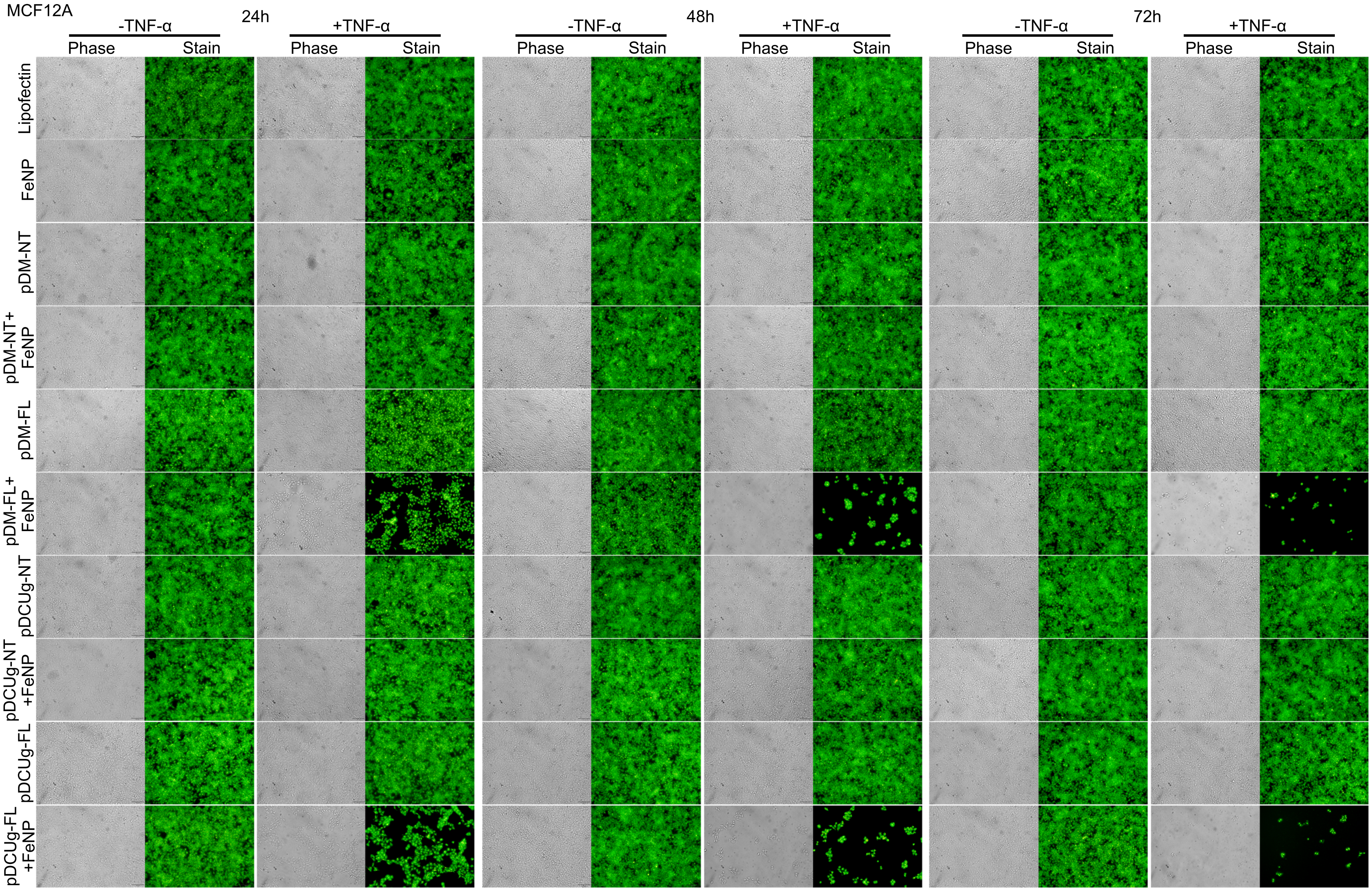

Supplementary Figure 25. Treatment of MCF12A cells with Gene interfering vectors and FeNP. Cells were transfected by various plasmids, cultured for 24 h and then induced with or without TNFα (10 ng/mL) for 1 h before incubating with or without 50 µg/mL of FeNP, and cells were cultured for another 72 h. At 24 h, 48 h and 72 h post FeNP administration, cells were stained with acridine orange and ethidium bromide and imaged under a fluorescence microscope. Scale bars, 100 µm. Representative data of two independent experiments. pDM-NT/pDM-FL, plas-mids expressing miRNAs under the control of DMP targeting no or *FPN&LCN2* transcripts; pDCUg-NT/pDCUg-FL, plasmids expressing Cas13a under the control of DMP and gRNAs under the control of U6 promoter targeting no or *FPN&LCN2* transcripts.

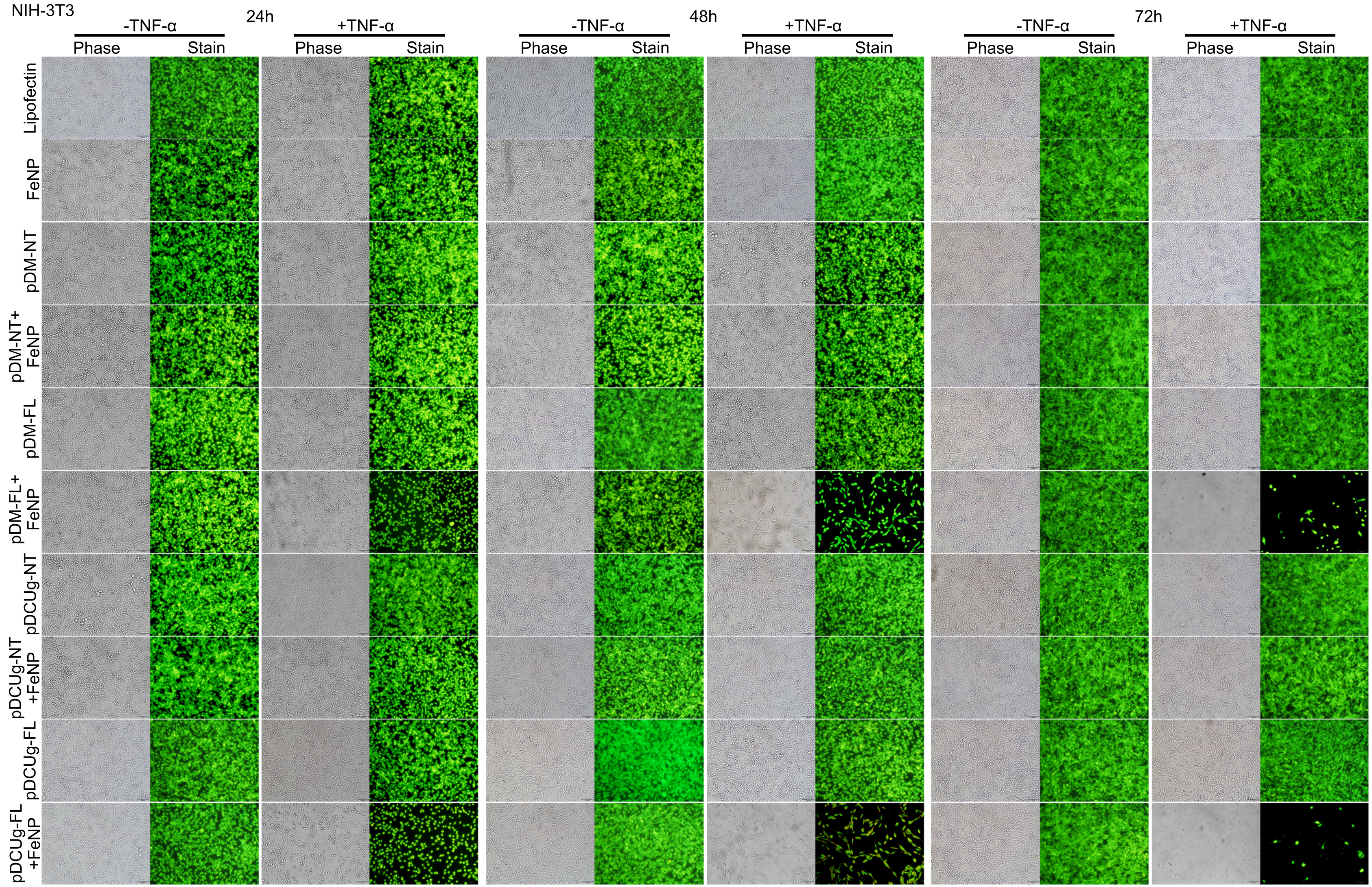

Supplementary Figure 26. Treatment of NIH-3T3 cells with Gene interfering vectors and FeNP. Cells were transfected by various plasmids, cultured for 24 h and then induced with or without TNFα (10 ng/mL) for 1 h before incubating with or without 50 μg/mL of FeNP, and cells were cultured for another 72 h. At 24 h, 48 h and 72 h post FeNP administration, cells were stained with acridine orange and ethidium bromide and imaged under a fluorescence microscope. Scale bars, 100 μm. Representative data of two independent experiments. pDM-NT/pDM-FL, plasmids expressing miRNAs under the control of DMP targeting no or *FPN&LCN2* transcripts; pDCUg-NT/pDCUg-FL, plasmids expressing Cas13a under the control of DMP and gRNAs under the control of U6 promoter targeting no or *FPN&LCN2* transcripts.

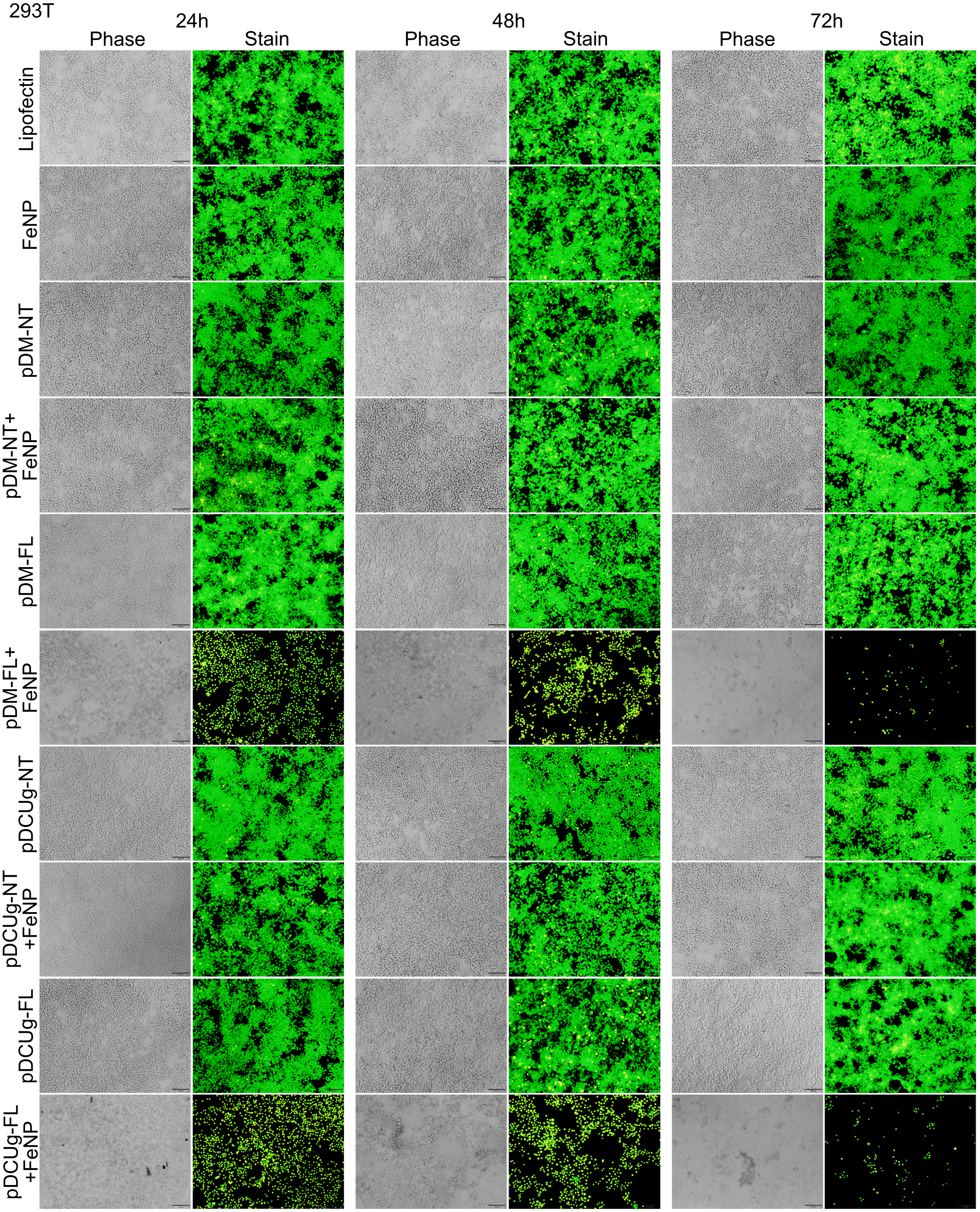

Supplementary Figure 27. Treatment of HEK-293T cells with Gene interfering vectors and FeNP. Cells were transfected by various plasmids, cultured for 24 h and then incubated with or without 50 μg/mL of FeNP, and cells were cultured for another 72 h. At 24 h, 48 h and 72 h post FeNP administration, cells were stained with acridine orange and ethidium bromide and imaged under a fluorescence microscope. Scale bars, 100 μm. Representative data of two independent experiments. pDM-NT/pDM-FL, plasmids expressing miRNAs under the control of DMP targeting no or *FPN&LCN2* transcripts; pDCUg-NT/pDCUg-FL, plasmids expressing Cas13a under the control of DMP and gRNAs under the control of U6 promoter targeting no or *FPN&LCN2* transcripts.

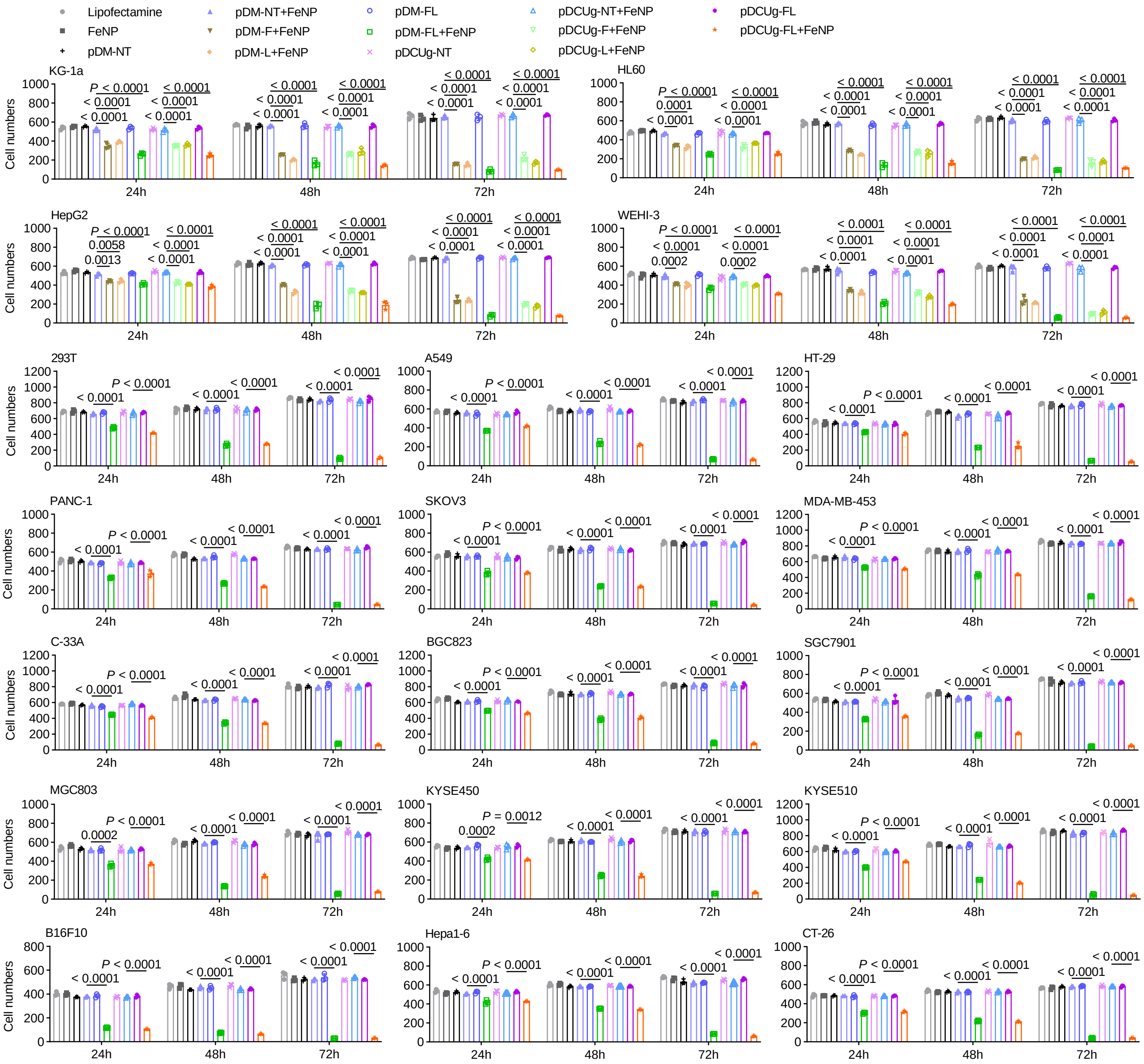

Supplementary Figure 28. Treatment of various cancer cells with GIFT. Cells including 19 cancer cells were treated with GIFT. All cells were detected at 24, 48, and 72 h post treatment of GIFT, respectively. Living cell numbers were counted from the fluorescent microscopic images of acridine orange and ethidium bromide (AO&EB)-stained cells by Image-Pro Plus software. The representative fluorescent microscopic images of AO&EB-stained cells were shown in Supplementary Figs. 2–19 and Supplementary Fig. 27. Data are presented as mean ± SD ($n = 3$ independent micrographs) and analyzed by two-way ANOVA with Tukey's test.

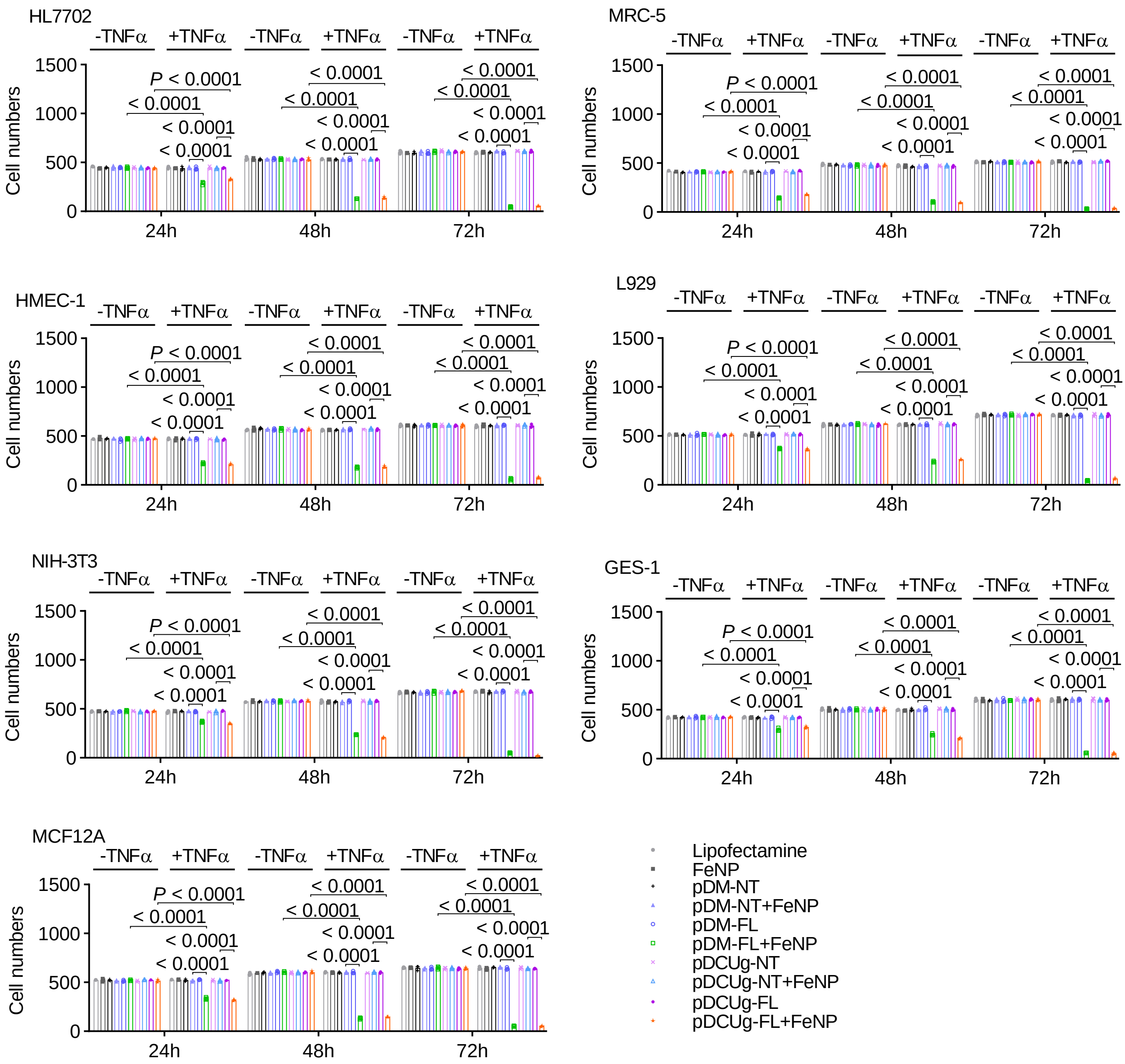

Supplementary Figure 29. Treatment of various normal cells with GIFT. Cells including 7 normal cells were treated with GIFT. All cells were detected at 24, 48, and 72 h post treatment of GIFT, respectively. Living cell numbers were counted from the fluorescent microscopic images of acridine orange and ethidium bromide (AO&EB)-stained cells by Image-Pro Plus software. The representative fluorescent microscopic images of AO&EB-stained cells were shown in Supplementary Figs. 20–26. Data are presented as mean ± SD (*n* = 3 independent micrographs) and analyzed by two-way ANOVA with Tukey's test.

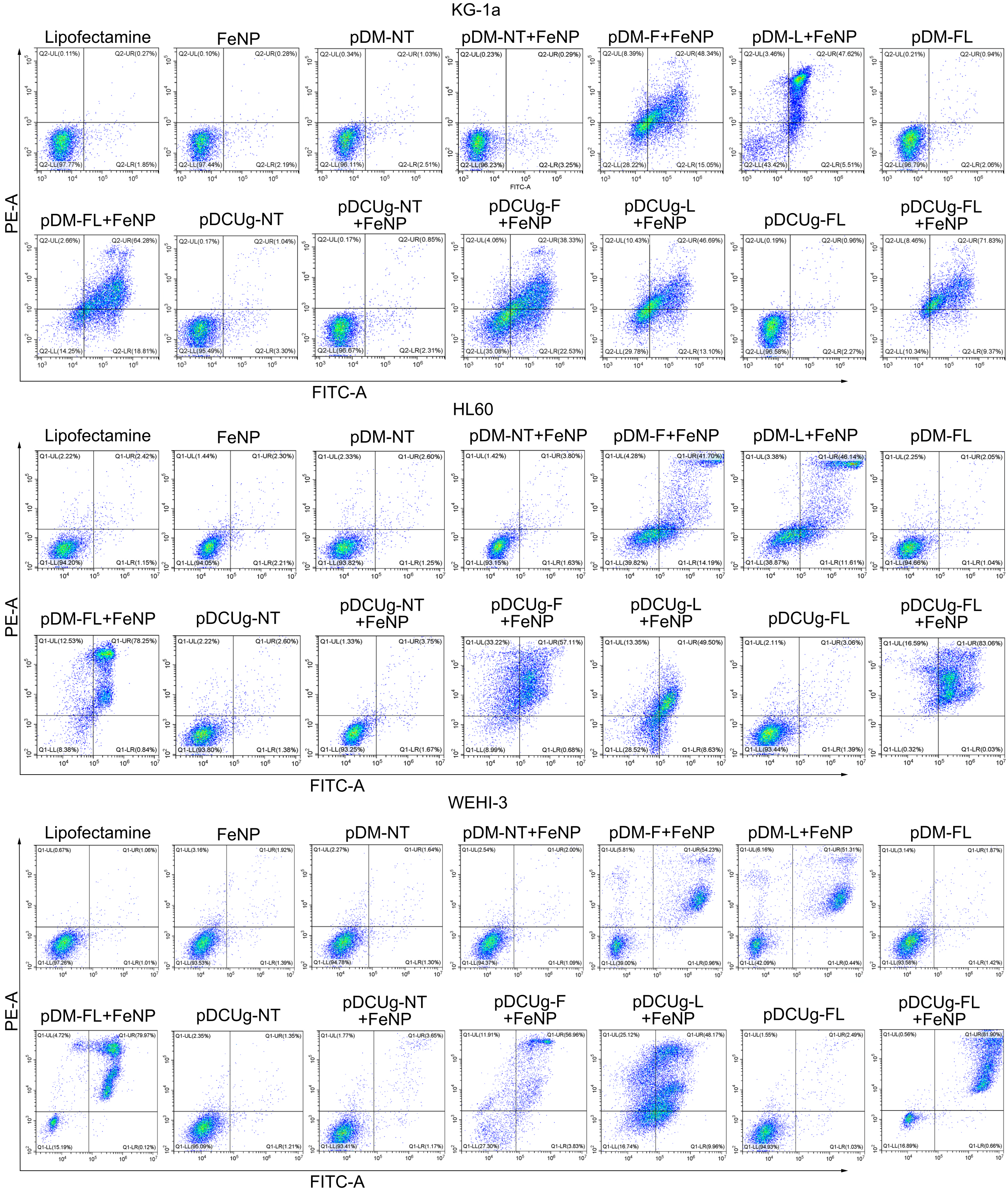

Supplementary Figure 30. Flow cytometry analysis of death of cells treated with gene interfering vectors and FeNP. KG-1a, HL60 and WEHI-3 cells were transfected by various plasmids, cultured for 24 h and then incubated with or without 50 μg/mL of FeNP. Cells were cultured for another 72 h. Cells were collected at 72 h post FeNP administration and detected with the Annexin V-FITC Apoptosis Detection Kit. The fluorescence intensity of cells was quantified by Flow Cytometer. pDM-NT/pDM-F/pDM-L/pDM-FL, plasmids expressing miRNAs under the control of DMP targeting no, FPN, LCN2, and *FPN&LCN2* transcripts; pDCUg-NT/pDCUg-F/pDCUg-L/pDCUg-FL, plasmids expressing Cas13a under the control of DMP and gRNAs under the control of U6 promoter targeting no, *FPN*, *LCN2*, and *FPN&LCN2* transcripts.

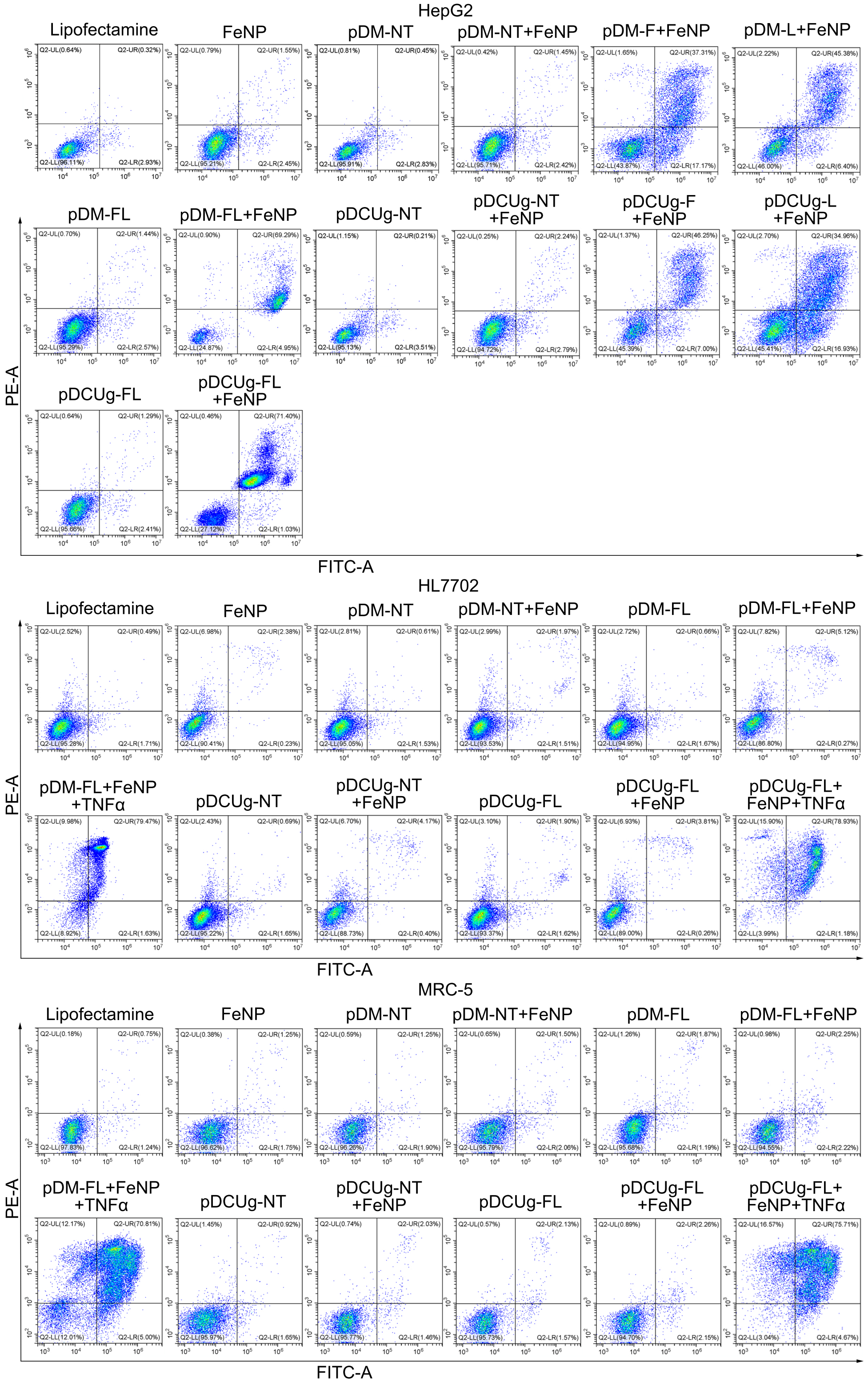

Supplementary Figure 31. Flow cytometry analysis of death of cells treated with gene interfering vectors and FeNP. HepG2, HL7702 and MRC-5 cells were transfected by various plasmids, cultured for 24 h and then incubated with or without 50 μg/mL of FeNP, and cells were cultured for another 72 h. For HL7702 and MRC-5, cells were first induced with or without TNFα (10 ng/mL) for 1h before FeNP treatment. Cells were collected at 72 h post FeNP administration and detected with the Annexin V-FITC Apoptosis Detection Kit. The fluorescence intensity of cells was quantified by Flow Cytometer. pDM-NT/pDM-F/pDM-L/pDM-FL, plasmids expressing miRNAs under the control of DMP targeting no, *FPN*, *LCN2*, or *FPN&LCN2* transcripts; pDCUg-NT/pDCUg-F/pDCUg-L/ pDCUg-FL, plasmids expressing Cas13a under the control of DMP and gRNAs under the control of U6 promoter targeting no, *FPN*, *LCN2*, or *FPN&LCN2* transcripts.

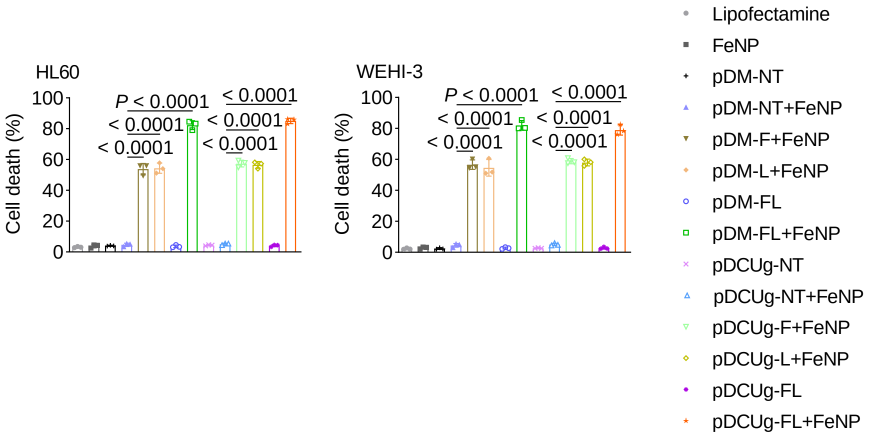

Supplementary Figure 32. Detection of cell death of HL60 and WEHI-3 cells after the GIFT treatment. The flow cytometer images are shown in Supplementary Figure 30. Data are presented as mean ± SD (n = 3 biologically independent samples) and analyzed by one-way ANOVA with Tukey's test. Plasmid used are as follows: pDCUg is pDMP-Cas13a-U6-gRNA; pDM is pDMP-miRNA; pDCUg-F and pDM-F target *FPN*; pDCUg-L and pDM-L target *LCN2*; pDCUg-FL and pDM-FL target both *FPN* and *LCN2*; pDCUg-NT and pDM-NT target no transcripts**.**

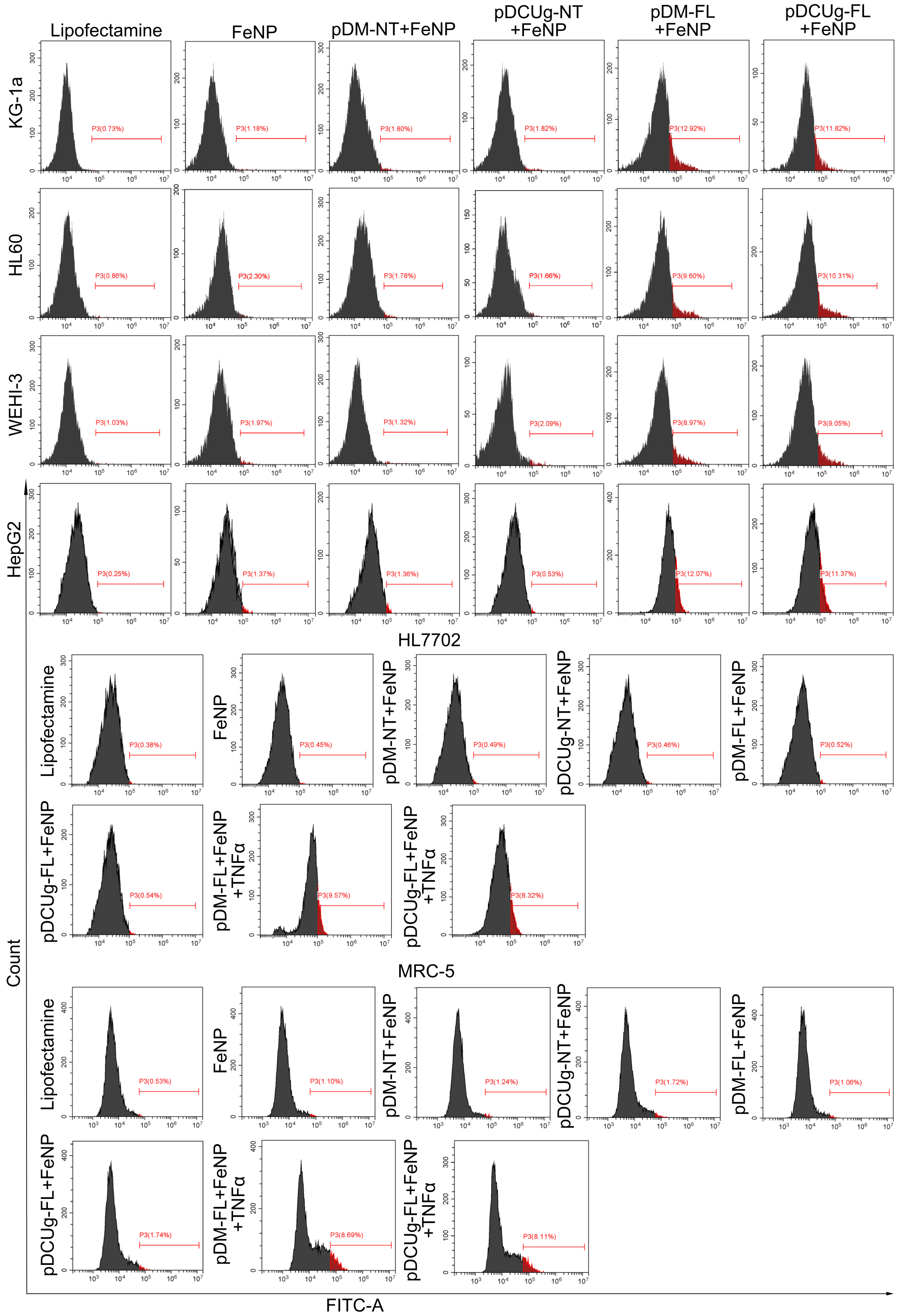

Supplementary Figure 33. Flow cytometry analysis of ROS levels of cells treated with gene interfer-ing vectors and FeNP. Cells were transfected by various plasmids, cultured for 24 h and then incubated with or without 50 μg/mL of FeNP, and cells were cultured for another 48 h. For HL7702 and MRC-5, cells were first induced with or without TNFα (10 ng/mL) for 1 h before FeNP treatment. Cells were collected at 48 h post FeNP administration and stained with DCFH-DA using Reactive Oxygen Species Assay Kit. ROS changes indicated by fluorescence shift were analyzed by Flow Cytometer. pDM-NT/pDM-FL, plasmids expressing miRNAs under the control of DMP targeting no or *FPN&LCN2* transcripts; pDCUg-NT/pDCUg-FL, plasmids expressing Cas13a under the control of DMP and gRNAs under the control of U6 promoter targeting no or *FPN&LCN2* transcripts.

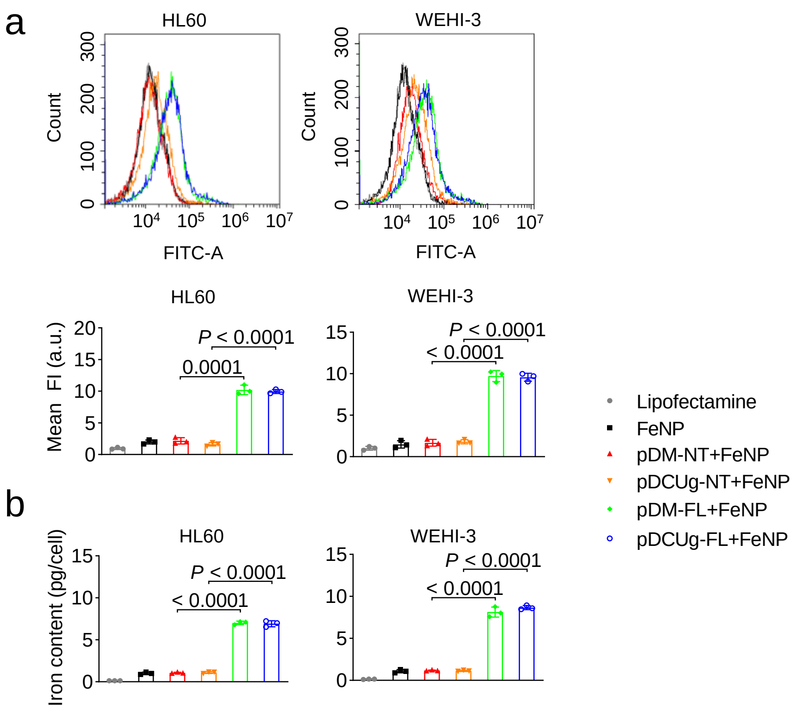

Supplementary Figure 34. ROS production and iron content in GIFT-treated cells. Cells were assayed at 48 h post FeNP treatment. **a,** Measurement of ROS levels. The fluorescence shift and quantified fluorescence intensity are shown. a.u., arbitrary units. **b,** Measurement of iron content. Data are presented as mean ± SD (n = 3 biologically independent samples) and analyzed by one-way ANOVA with Tukey's test. The figure shows the data of four cell lines (HL60 and WEHI-1). The representative raw data of flow cytometry detection of ROS are shown in Supplementary Fig.33. Figure a and b used a same set of symbols.

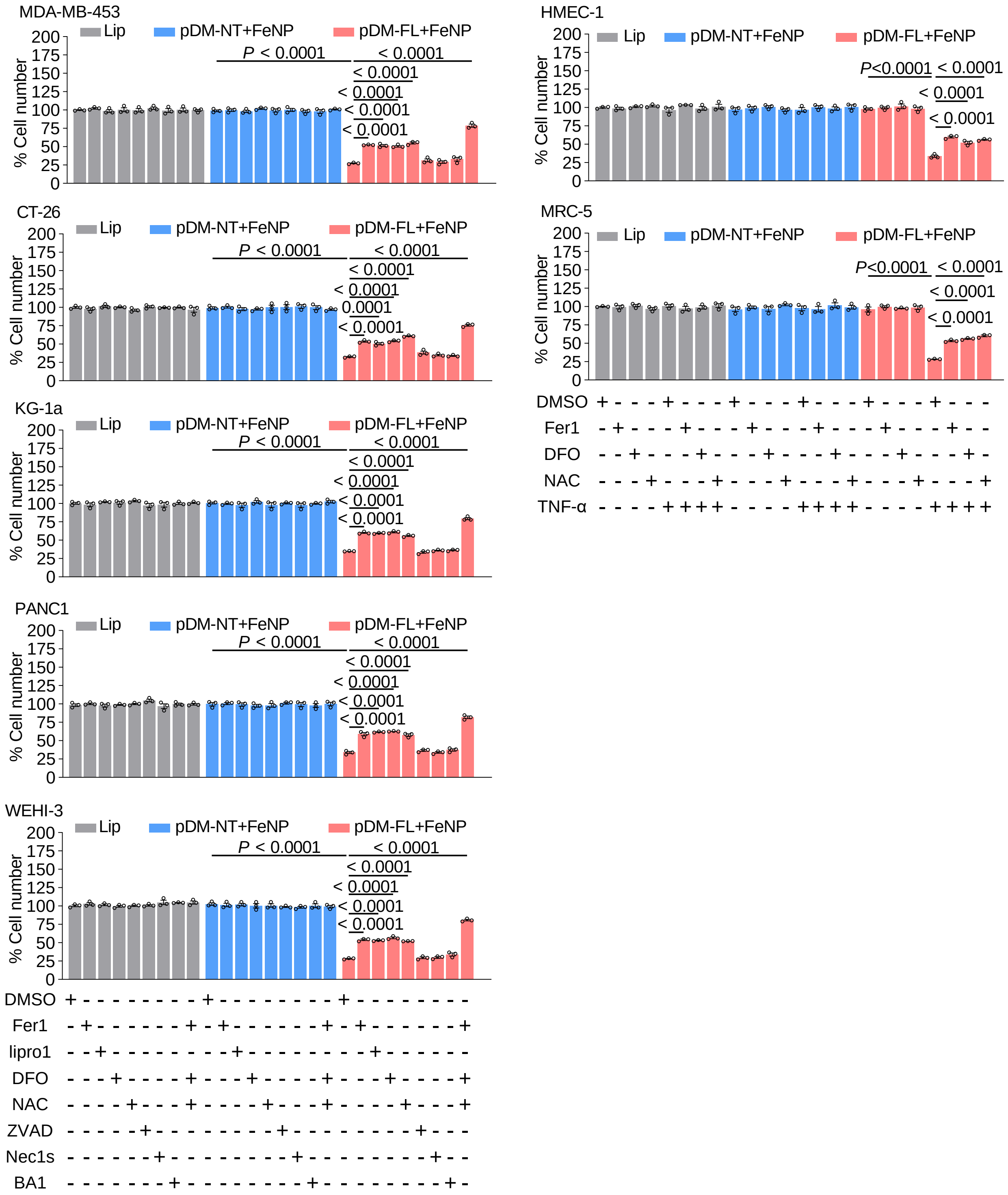

Supplementary Figure 35. Cell viability detected by Cell-Titer-Glo 2.0 reagent. Cells were transfected with various plasmids and cultured for 24 h. The treated cells were then co-incubated with FeNP and indicated inhibitors for 48 h. For HMEC-1 and MRC-5 cells, cells were induced with or without TNFα (10 ng/mL) for 1 h before incubating FeNP and indicated inhibitors. Data are presented as mean±SD (*n* = 3 biologically independent samples) and analyzed by one-way ANOVA with Tukey's test. Lip, Lipofectamine; Fer1, Ferrostatin-1 (1 μM); lipro1, liproxstatin-1 (1 μM); DFO, deferoxamine (100 μM); NAC, N-acetylcysteine (1 mM); ZVAD, ZVAD-FMK (50 μM); Nec1s, Necrostatin-1s (10 μM); BA1, Bafilomycin A1 (1 nM).

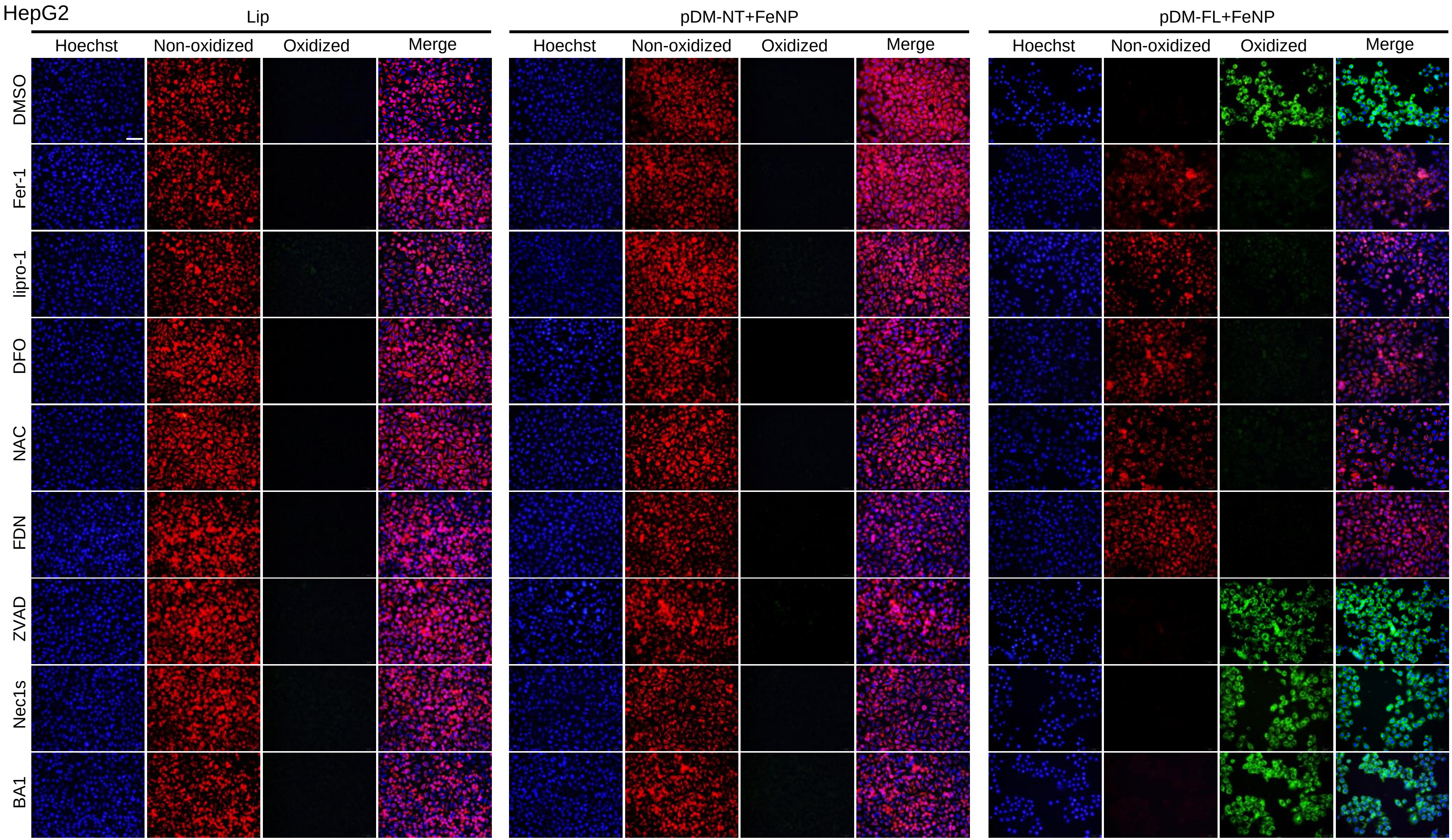

Supplementary Figure 36. Detection of lipid ROS. Representative images of HepG2 cells stained with C11-BODIPY. Cells were transfected with various plasmids and co-incubated with FeNP and indicated inhibitors for 48 h. Cells were then stained with C11-BODIPY and imaged by fluorescence microscope. Scale bars, 50 µm. Representative data of two independent experiments. Blue, nucleus; red, reduced dye; green, oxidized dye. Lip, Lipofectamine; Fer1, Ferrostatin-1 (1 µM); lipro1, liproxstatin-1 (1 µM); DFO, deferoxamine (100 µM); NAC, N-acetylcysteine (1 mM); ZVAD, ZVAD-FMK (50 µM); Nec1s, Necrostatin-1s (10 µM); BA1, Bafilomycin A1 (1 nM).

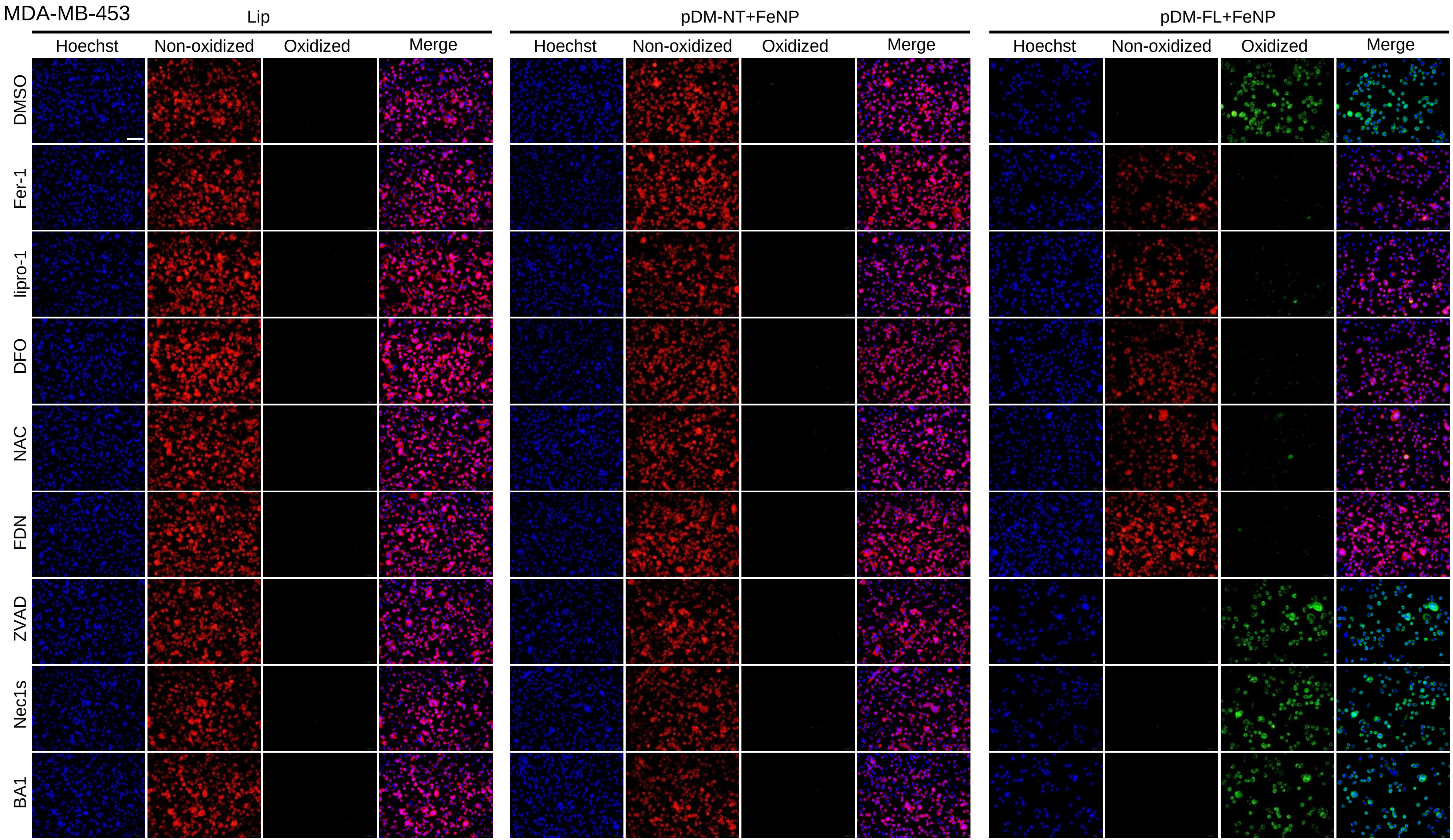

Supplementary Figure 37. Detection of lipid ROS. Representative images of MDA-MB-453 cells stained with C11-BODIPY. Cells were transfected with various plasmids and co-incubated with FeNP and indicated inhibitors for 48 h. Cells were then stained with C11-BODIPY and imaged by fluorescence microscope. Scale bars, 50 µm. Representative data of two independent experiments. Blue, nucleus; red, reduced dye; green, oxidized dye. Lip, Lipofectamine; Fer1, Ferrostatin-1 (1 µM); lipro1, liproxstatin-1 (1 µM); DFO, deferoxamine (100 µM); NAC, N-acetylcysteine (1 mM); ZVAD, ZVAD-FMK (50 µM); Nec1s, Necrostatin-1s (10 µM); BA1, Bafilomycin A1 (1 nM).

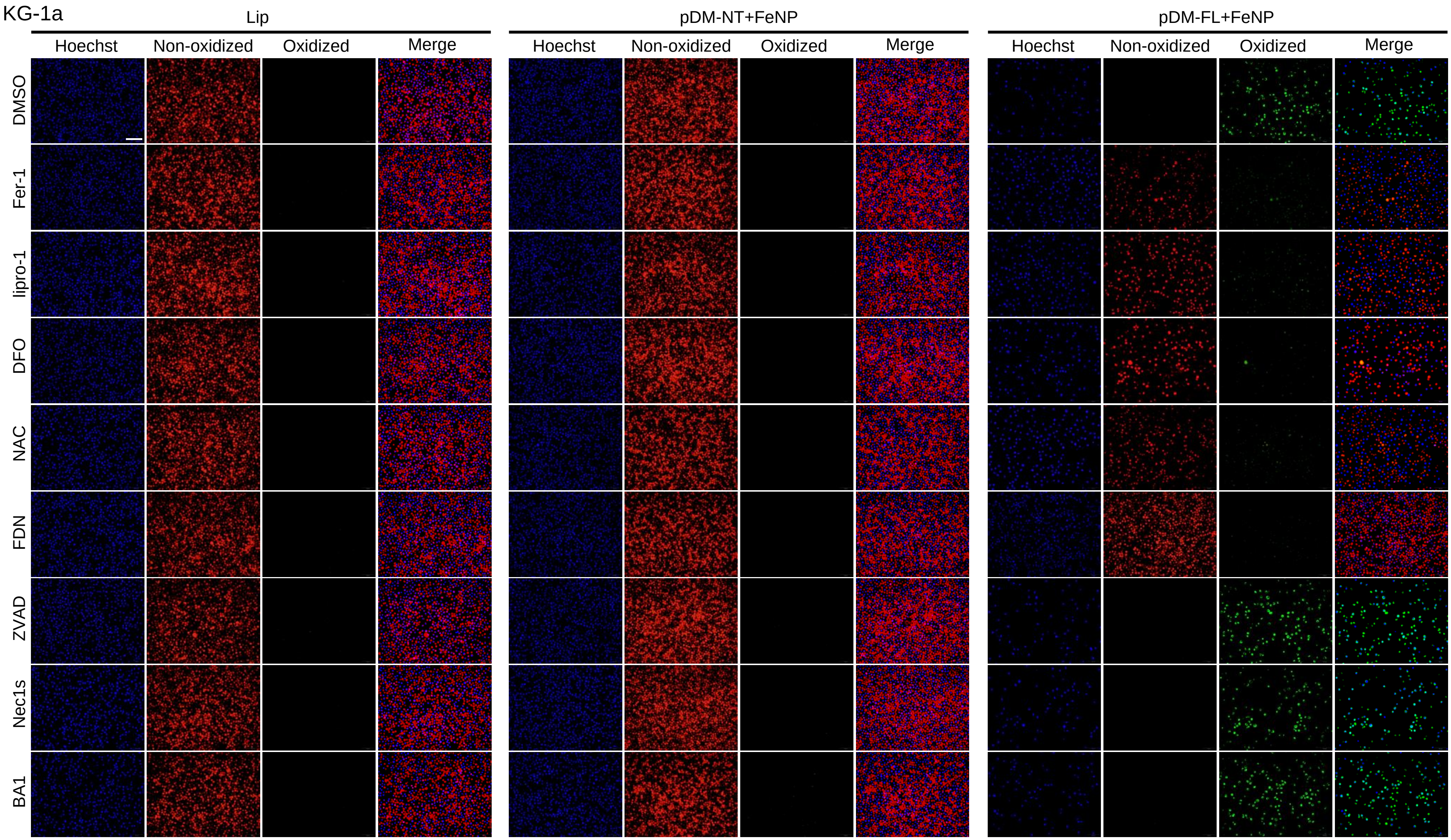

Supplementary Figure 38. Detection of lipid ROS. Representative images of KG-1a cells stained with C11-BODIPY. Cells were transfected with various plasmids and co-incubated with FeNP and indicated inhibitors for 48 h. Cells were then stained with C11-BODIPY and imaged by fluorescence microscope. Scale bars, 50 μm. Representative data of two independent experiments. Blue, nucleus; red, reduced dye; green, oxidized dye. Lip, Lipofectamine; Fer1, Ferrostatin-1 (1 μM); lipro1, liproxstatin-1 (1 μM); DFO, deferoxamine (100 μM); NAC, N-acetylcysteine (1 mM); ZVAD, ZVAD-FMK (50 μM); Nec1s, Necrostatin-1s (10 μM); BA1, Bafilomycin A1 (1 nM).

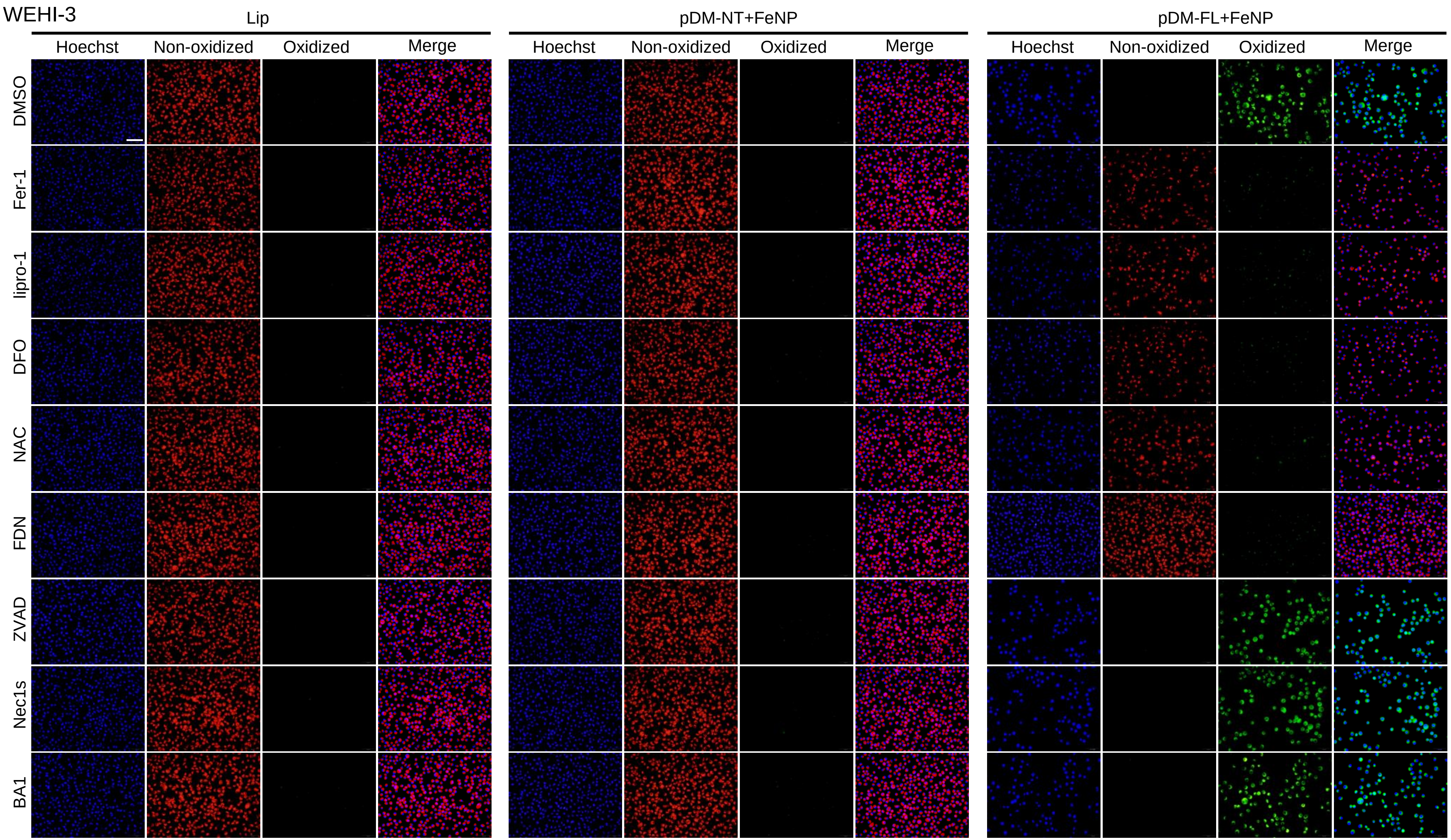

Supplementary Figure 39. Detection of lipid ROS. Representative images of WEHI-3 cells stained with C11-BODIPY. Cells were transfected with various plasmids and co-incubated with FeNP and indicated inhibitors for 48 h. Cells were then stained with C11-BODIPY and imaged by fluorescence microscope. Scale bars, 50 µm. Representative data of two independent experiments. Blue, nucleus; red, reduced dye; green, oxidized dye. Lip, Lipofectamine; Fer1, Ferrostatin-1 (1 µM); lipro1, liproxstatin-1 (1 µM); DFO, deferoxamine (100 µM); NAC, N-acetylcysteine (1 mM); ZVAD, ZVAD-FMK (50 µM); Nec1s, Necrostatin-1s (10 µM); BA1, Bafilomycin A1 (1 nM).

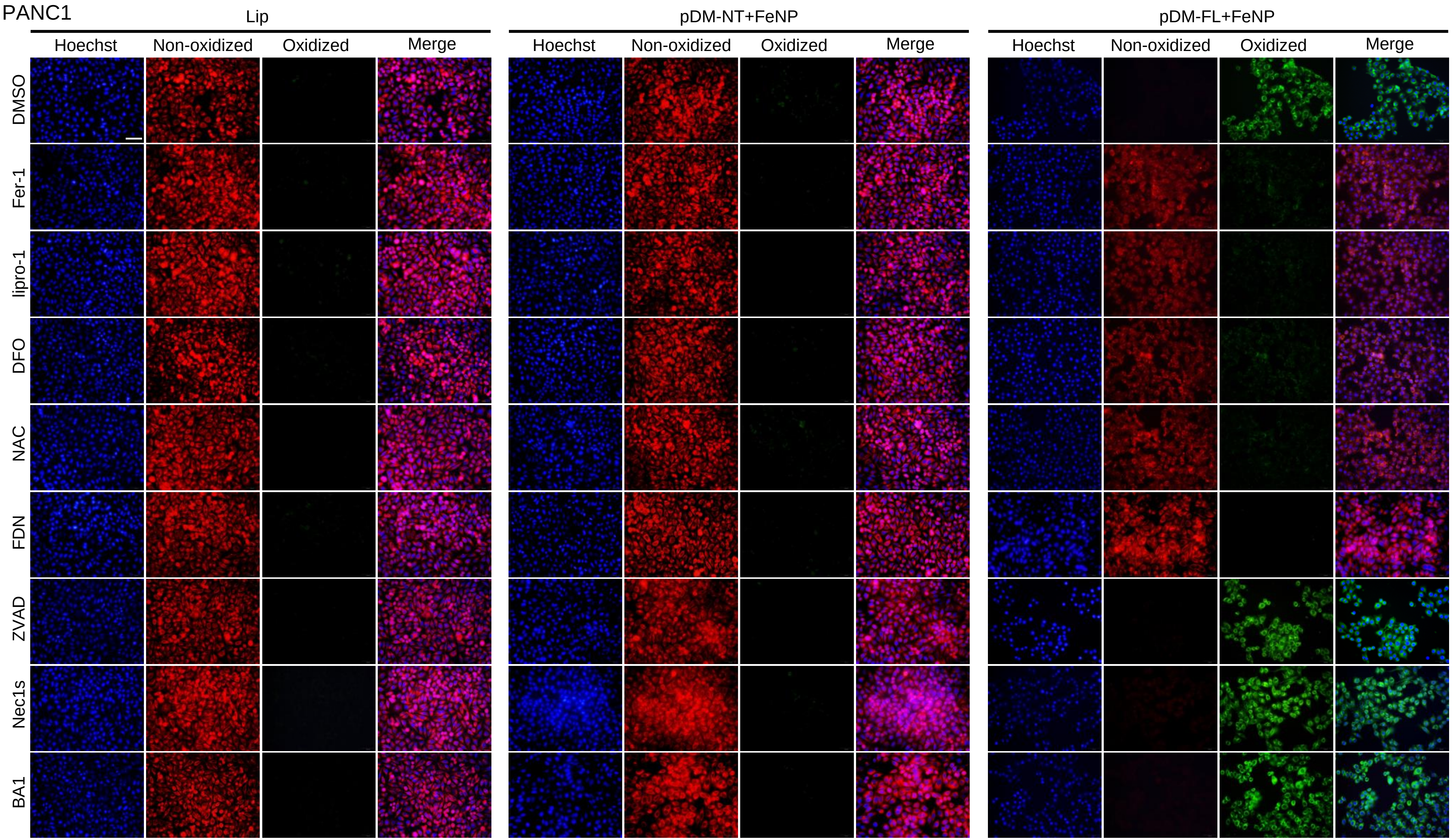

Supplementary Figure 40. Detection of lipid ROS. Representative images of PANC1 cells stained with C11-BODIPY. Cells were transfected with various plasmids and co-incubated with FeNP and indicated inhibitors for 48 h. Cells were then stained with C11-BODIPY and imaged by fluorescence microscope. Scale bars, 50 μm. Representative data of two independent experiments. Blue, nucleus; red, reduced dye; green, oxidized dye. Lip, Lipofectamine; Fer1, Ferrostatin-1 (1 μM); lipro1, liproxstatin-1 (1 μM); DFO, deferoxamine (100 μM); NAC, N-acetylcysteine (1 mM); ZVAD, ZVAD-FMK (50 μM); Nec1s, Necrostatin-1s (10 μM); BA1, Bafilomycin A1 (1 nM).

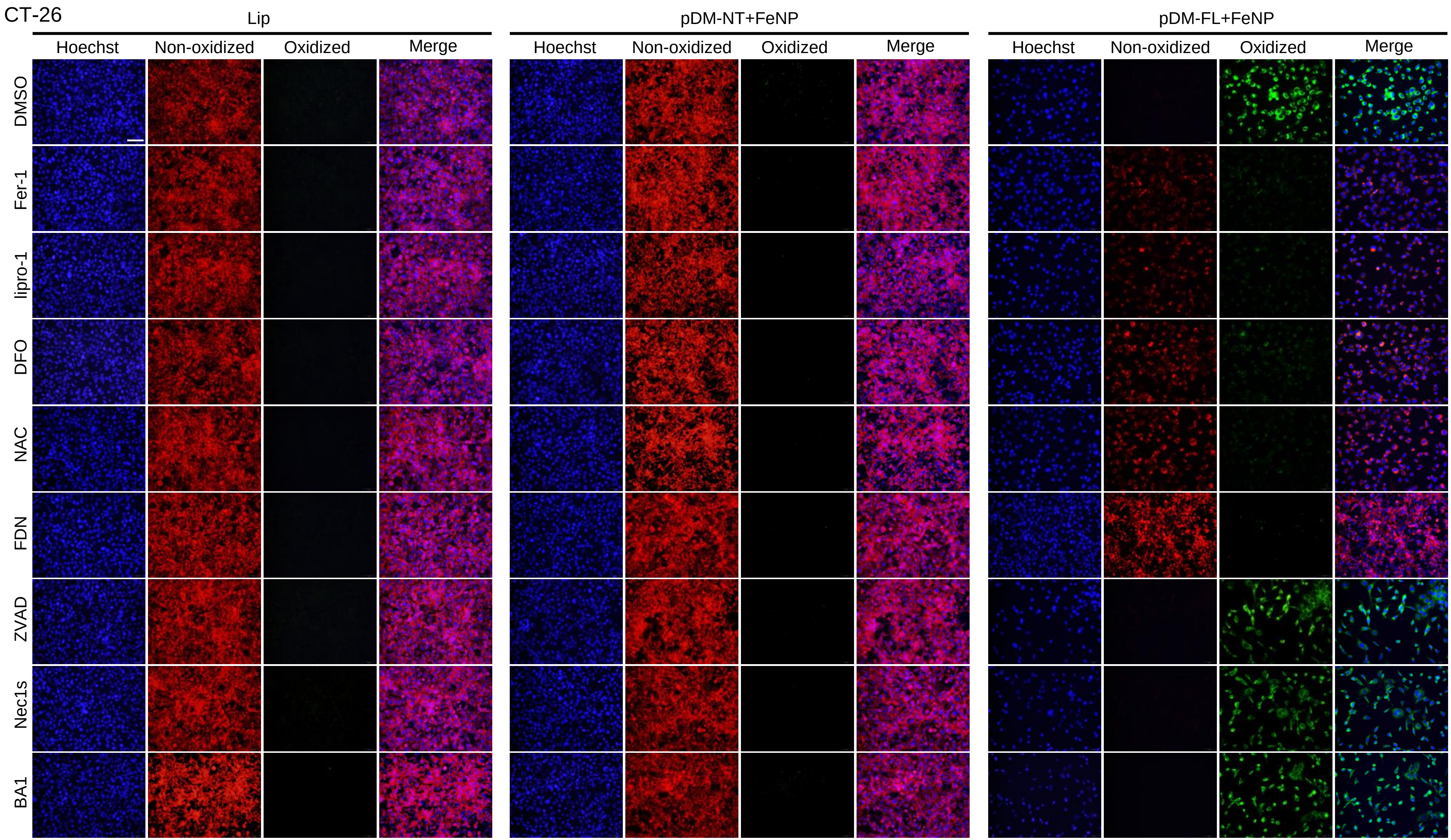

Supplementary Figure 41. Detection of lipid ROS. Representative images of CT-26 cells stained with C11-BODIPY. Cells were transfected with various plasmids and co-incubated with FeNP and indicated inhibitors for 48 h. Cells were then stained with C11-BODIPY and imaged by fluorescence microscope. Scale bars, 50 µm. Representative data of two independent experiments. Blue, nucleus; red, reduced dye; green, oxidized dye. Lip, Lipofectamine; Fer1, Ferrostatin-1 (1 µM); lipro1, liproxstatin-1 (1 µM); DFO, deferoxamine (100 µM); NAC, N-acetylcysteine (1 mM); ZVAD, ZVAD-FMK (50 µM); Nec1s, Necrostatin-1s (10 µM); BA1, Bafilomycin A1 (1 nM).

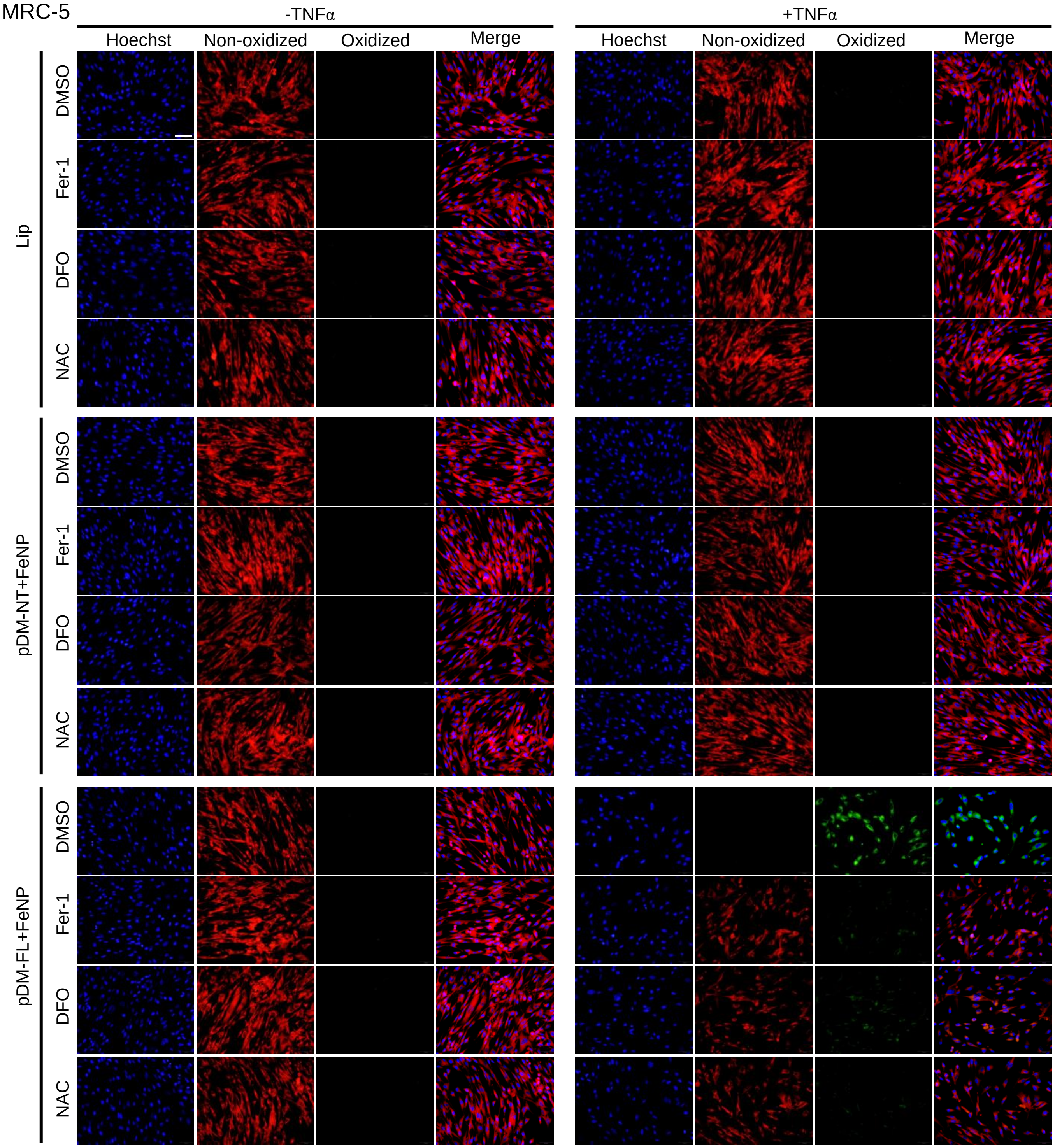

Supplementary Figure 42. Detection of lipid ROS. Representative images of MRC-5 cells stained with C11-BODIPY. Cells were transfected with various plasmids, cultured for 24 h and then induced with or without TNFα (10 ng/mL) for 1 h before incubating FeNP and indicated inhibitors for 48 h. Cells were then stained with C11-BODIPY and imaged by fluorescence microscope. Scale bars, 50 μm. Representative data of two independent experiments. Blue, nucleus; red, reduced dye; green, oxidized dye. Lip, Lipofectamine; Fer1, Ferrostatin-1 (1 μM); DFO, deferoxamine (100 μM); NAC, N-acetylcysteine (1 mM).

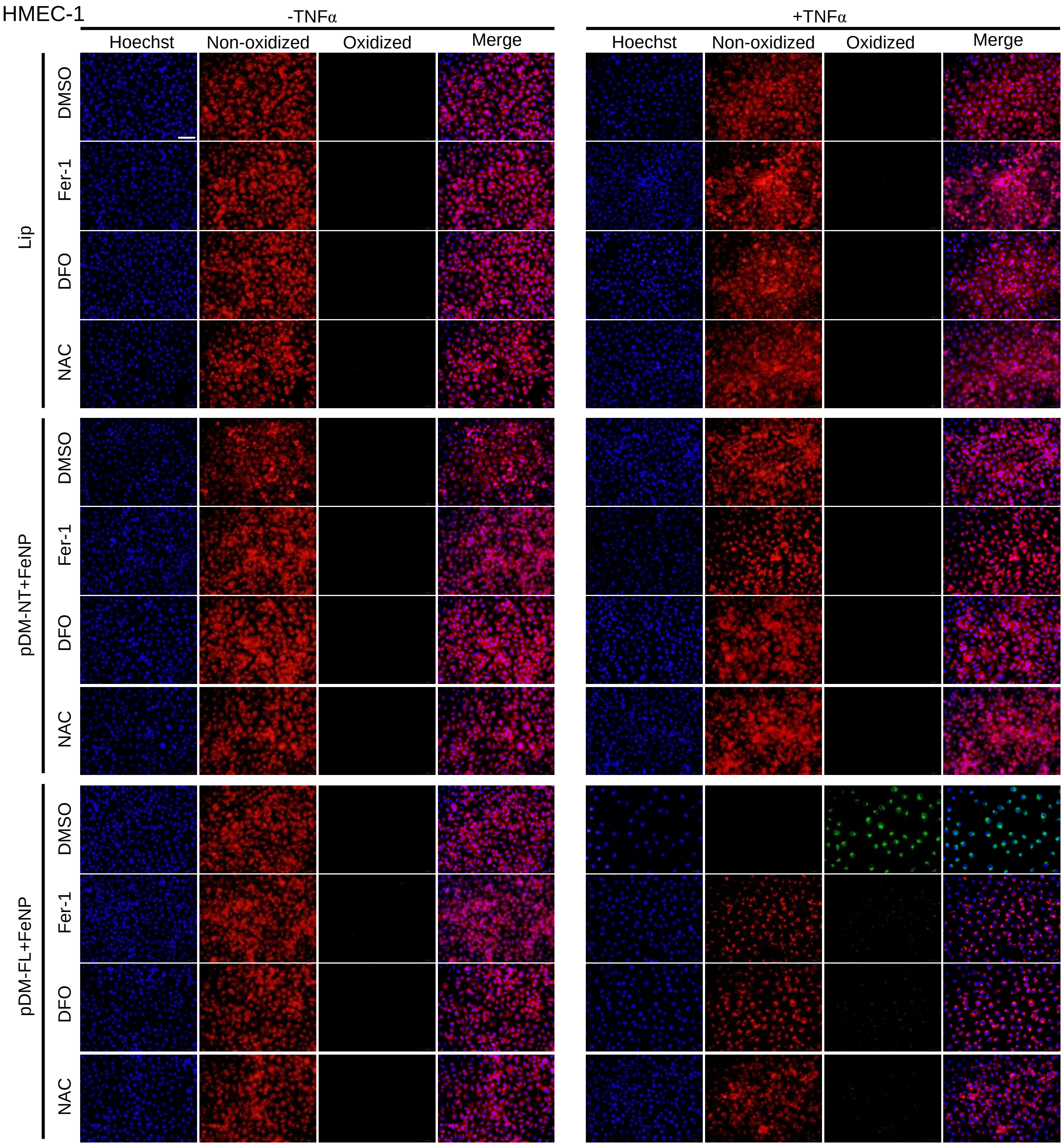

Supplementary Figure 43. Detection of lipid ROS. Representative images of HMEC-1 cells stained with C11-BODIPY. Cells were transfected with various plasmids, cultured for 24 h and then induced with or without TNFα (10 ng/mL) for 1 h before incubating FeNP and indicated inhibitors for 48 h. Cells were then stained with C11-BODIPY and imaged by fluorescence microscope. Scale bars, 50 μm. Representative data of two independent experiments. Blue, nucleus; red, reduced dye; green, oxidized dye. Lip, Lipofectamine; Fer1, Ferrostatin-1 (1 μM); DFO, deferoxamine (100 μM); NAC, N-acetylcysteine (1 mM).

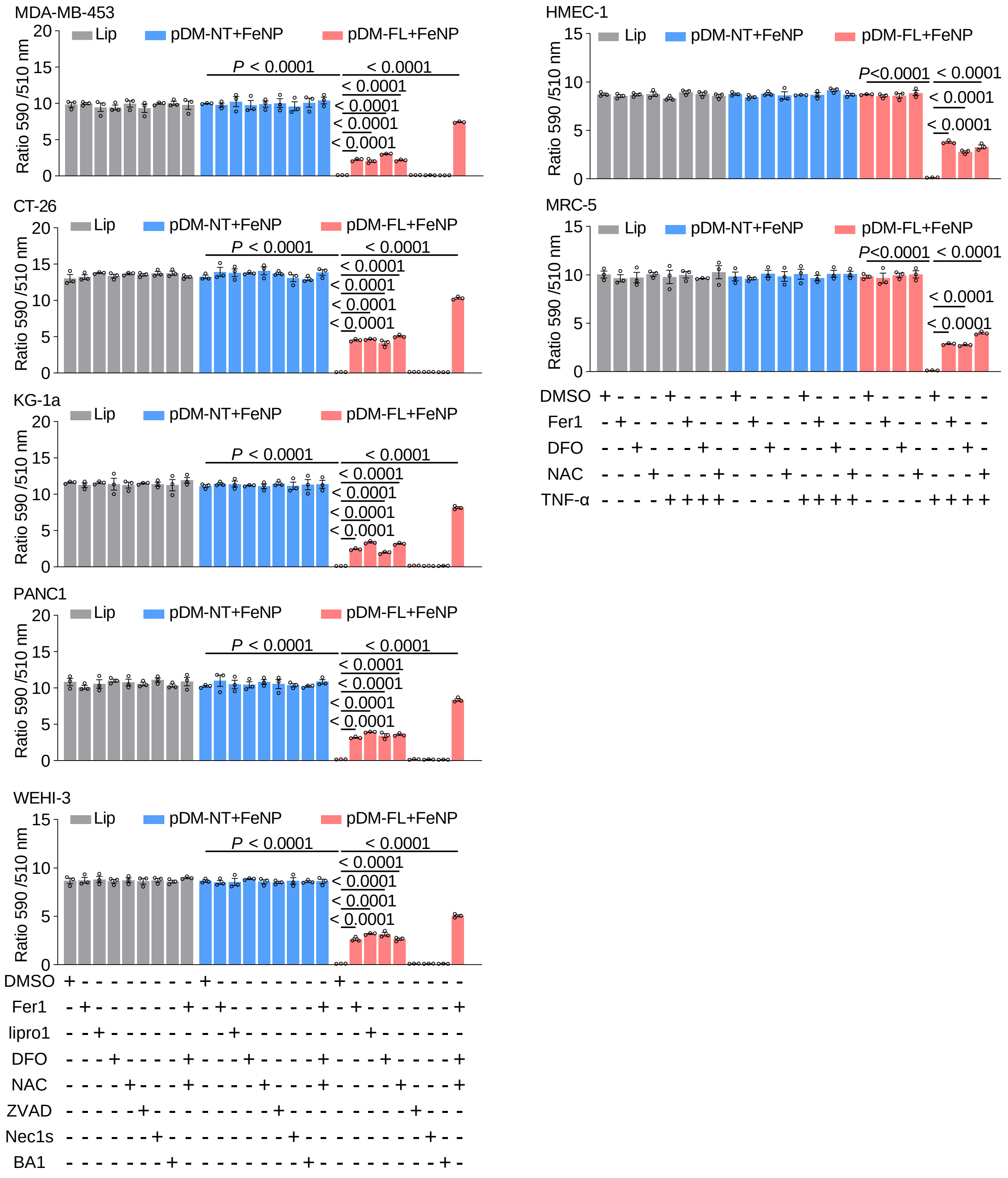

Supplementary Figure 44. Quantified results of lipid peroxidation in Supplementary figure 37-43. The lipid peroxidation in cells were determined by quantitating the fluorescence intensities analyzed by ImageJ software and calculating the ratio of intensity in 590 to 510 channels. Data are presented as mean ± SD ($n = 3$ independent micrographs) and analyzed by one-way ANOVA with Tukey's test. Lip, Lipofectamine; Fer1, Ferrostatin-1 (1 μM); lipro1, liproxstatin-1 (1 μM); DFO, deferoxamine (100 μM); NAC, N-acetylcysteine (1 mM); ZVAD, ZVAD-FMK (50 μM); Nec1s, Necrostatin-1s (10 μM); BA1, Bafilomycin A1 (1 nM).

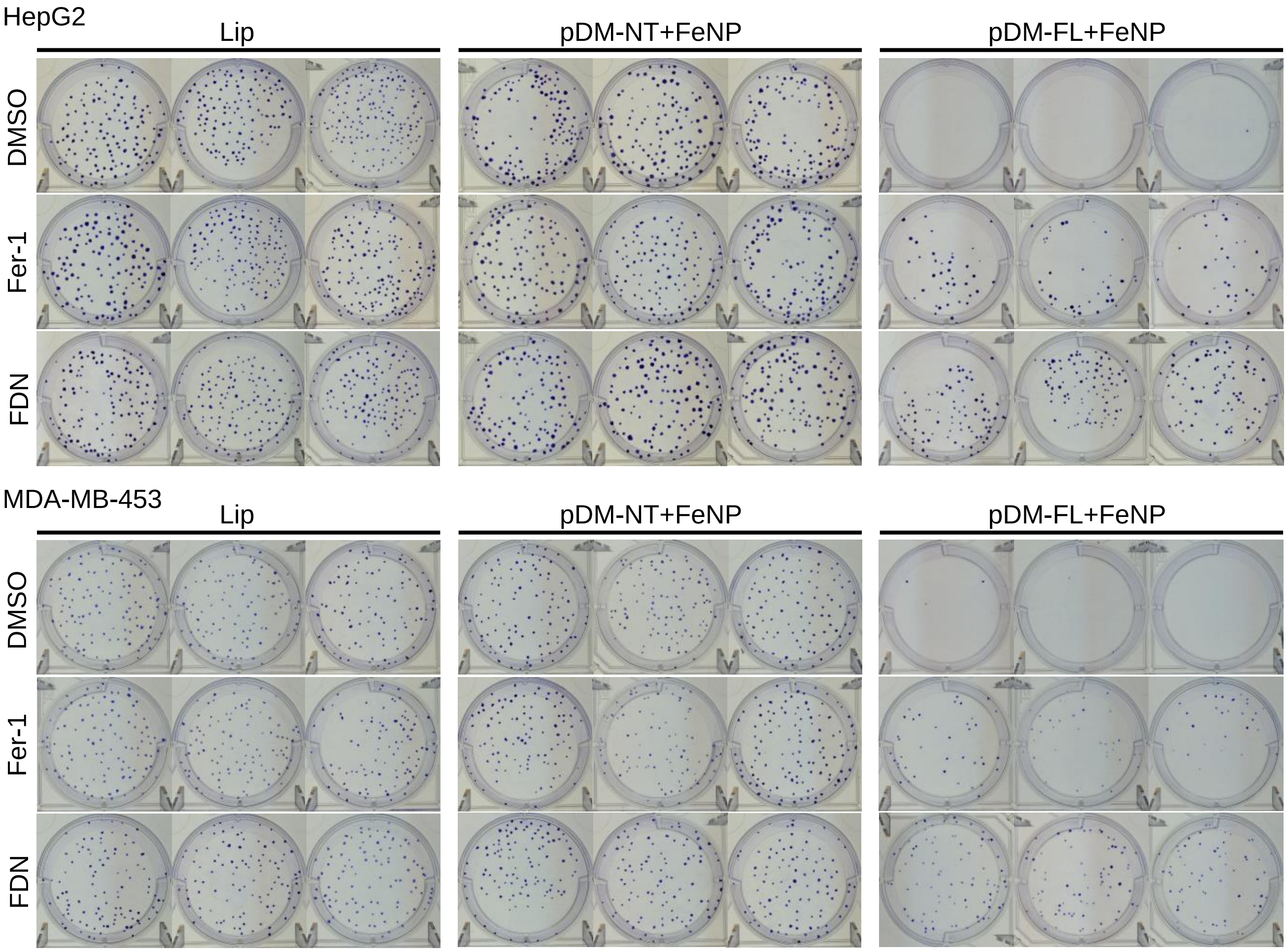

Supplementary Figure 45. Representative clone growth pictures of HepG2 and MDA-MB-453 cells. Cells were transfected with various plasmids including. The transfected cells were cultured for 24 h and then incubated with 50 μg/mL of FeNP and various inhibitors. 48h later, the treated cells were washed, trypsinized, counted, replated into 6-well plates at a density of 200 cells/well. When colonies of >50 cells are clearly visible, colonies were fixed with 4% paraformaldehyde, stained with 0.1% crystal violet and counted. Lip, Lipofectamine; Fer1, Ferrostatin-1 (1 µM); DFO, deferoxamine (100 µM); NAC, N-acetylcysteine (1 mM); FDN, co-incubation of Fer1, DFO and NAC.

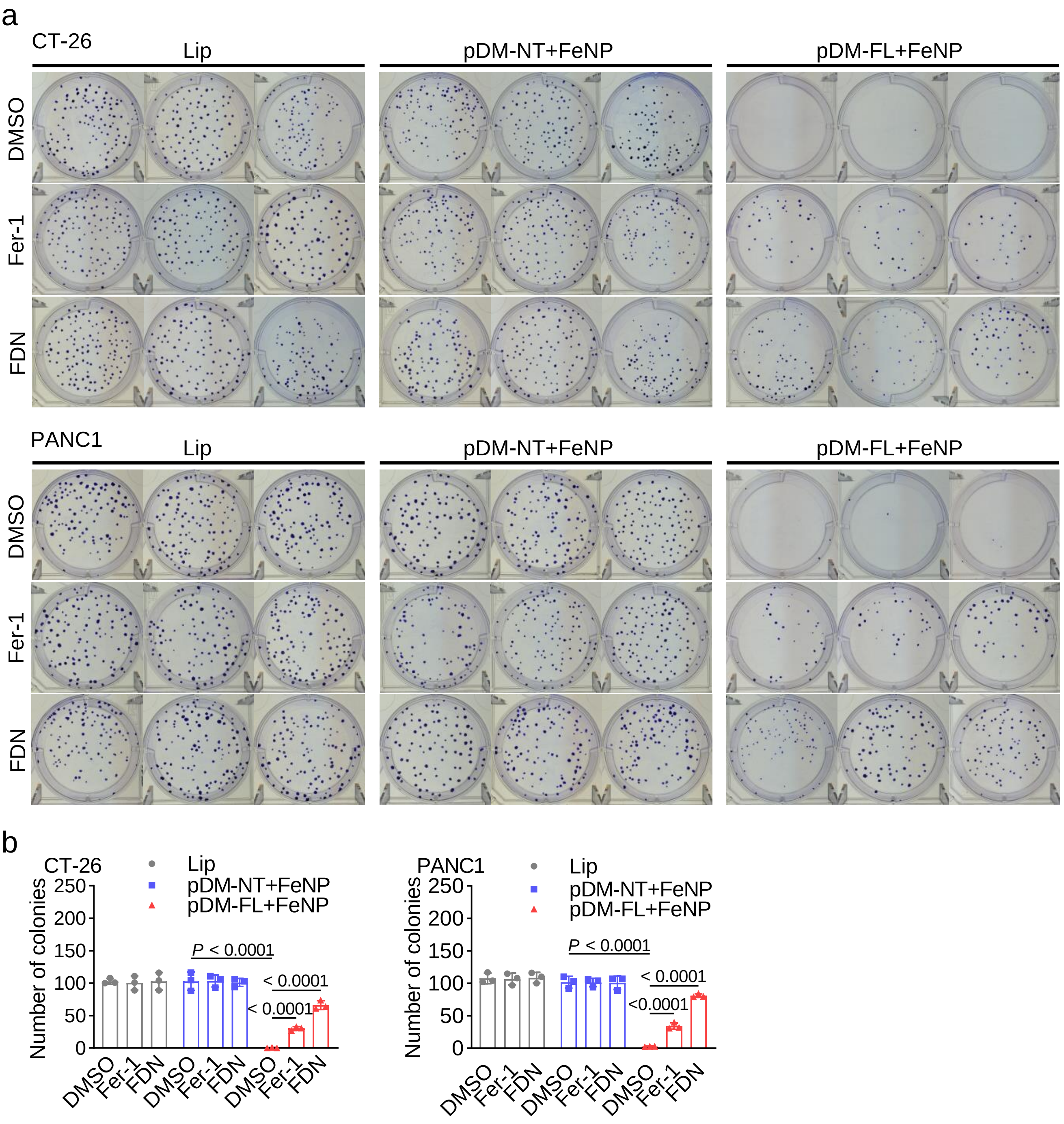

Supplementary Figure 46. Clone formation assay of CT-26 and PANC1 cells. Cells were transfected with various plasmids. The transfected cells were cultured for 24 h and then incubated with 50 µg/mL of FeNP and various inhibitors. 48h later, the treated cells were washed, trypsinized, counted, replated into 6-well plates at a density of 200 cells/ well. When colonies of >50 cells are clearly visible, colonies were fixed with 4% paraformaldehyde, stained with 0.1% crystal violet and counted. **a,** Representative clone growth pictures of HepG2 and MDA-MB-453 cells. **b,** Quantification of colony numbers in a. Data are presented as mean ± SD ($n = 3$ biologically independent samples) and analyzed by one-way ANOVA with Tukey's test. Lip, Lipofectamine; Fer1, Ferrostatin-1 (1 µM); DFO, deferoxamine (100 µM); NAC, N-acetylcysteine (1 mM); FDN, co-incubation of Fer1, DFO and NAC.

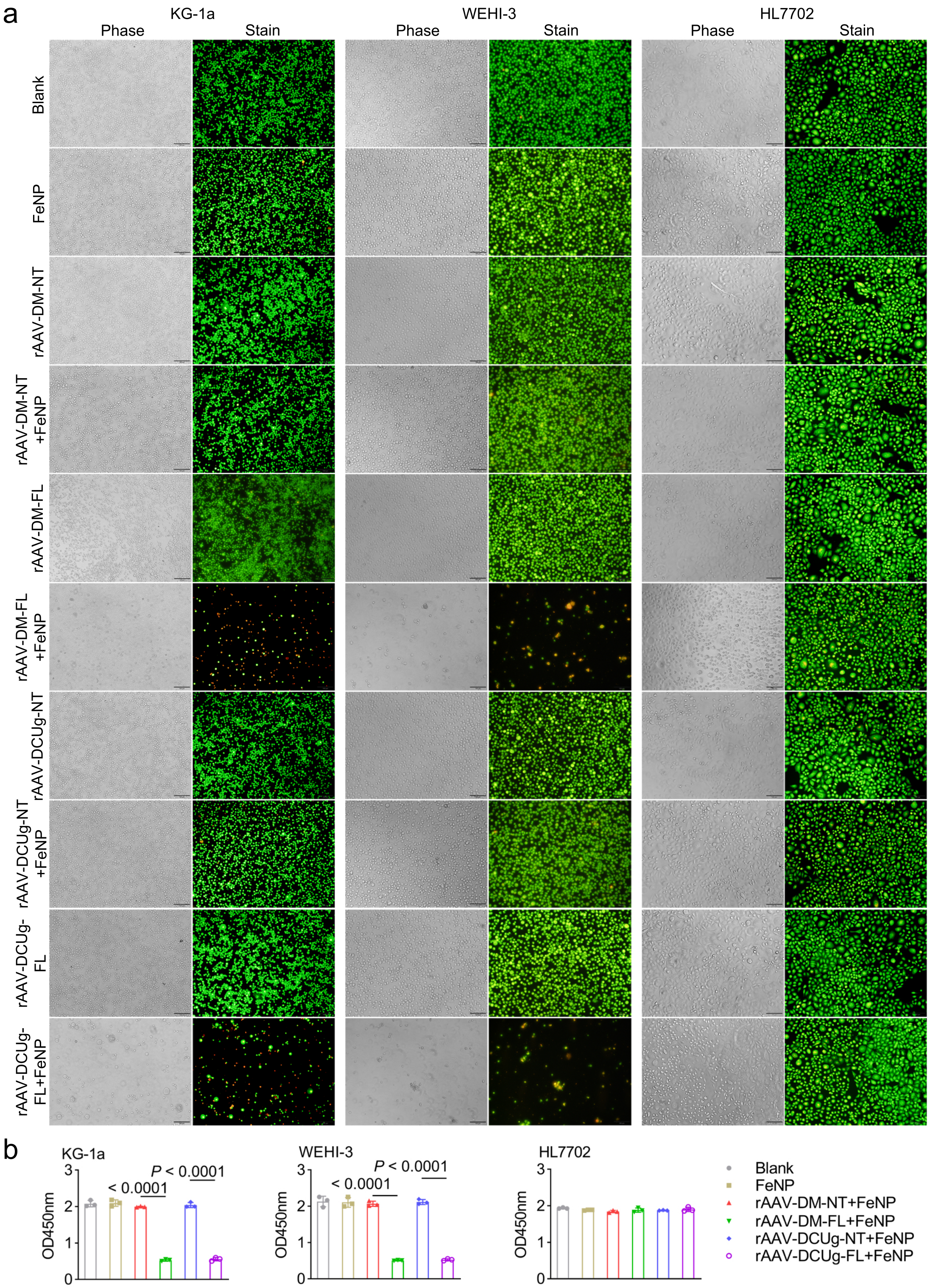

Supplementary Figure 47. Evaluation of rAAVs in vitro. KG-1a, WEHI-3 and HL7702 cells were seeded into 24-well plates (1×10^5 cells/well) and cultivated for 12 h. Cells were then transfected with the various viruses at the dose of 1×10^5 vg per cell. The transfected cells were cultured for 24 h and then incubated with or without 50 μg/mL FeNP. Cells were cultured for another 72 h. All cells were stained with acridine orange and ethidium bromide and imaged by optical microscope, and cell viability was evaluated using a CCK-8 assay. Scale bars, 100 μm. **a**, Representative images of cells at 72 h post FeNP administration. **b**, The cell viability at 72 h post transfection. Data are presented as mean ± SD (n = 3 biologically independent samples) and analyzed by one-way ANOVA with Tukey's test. rAAV-DM-NT/rAAV-DM-FL, viruses expressing miRNAs under the control of DMP targeting no or *FPN&LCN2* transcripts; rAAV-DCUg-NT/rAAV-DCUg-FL, viruses expressing Cas13a under the control of DMP and gRNAs under the control of U6 promoter targeting no or *FPN&LCN2* transcripts.

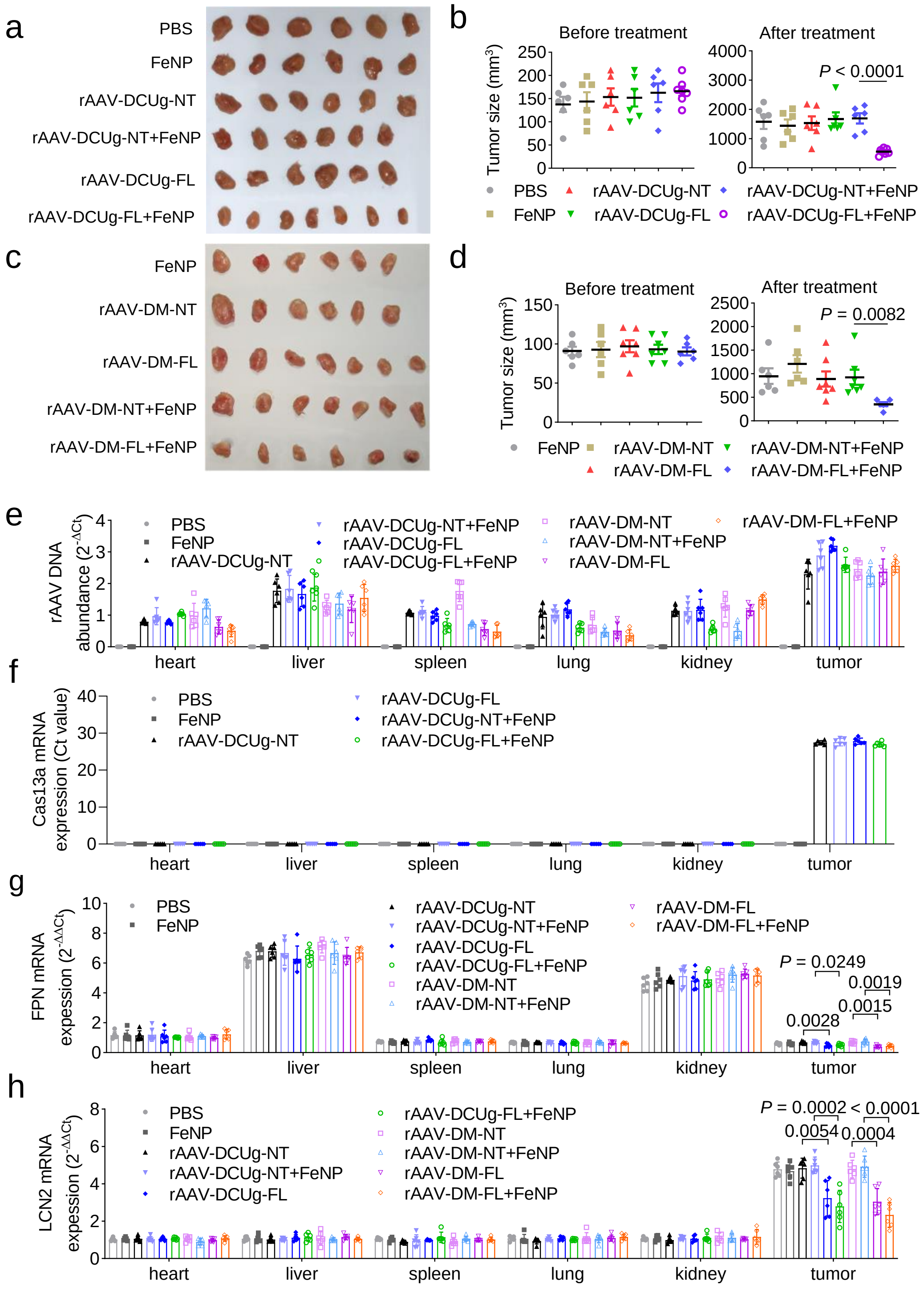

Supplementary Figure 48. The in vivo antitumor effects of virus-based GIFT in WEHI-3 xenografted mice. Mice were treated by single time intravenous injection of various reagents. rAAV, 1×10^{10} vg/mouse; FeNP, 3 mg/kg. **a** and **b,** Mice treated by rAAV-DCUg and FeNP (for rAAV-DCUg-FL+FeNP group, n = 7 mice; for other groups, n = 6 mice). **c** and **d,** Mice treated by rAAV-DM and FeNP (for rAAV-DM-FL and rAAV-DM-NT+FeNP groups, n = 7 mice; for other groups, n = 6 mice). **a** and **c,** Tumor images. **b** and **d,** Tumor volumes before and after treatment. **e,** Abundance of virus DNA in tissues. **f,** Cas13a expression in tissues. **g,** *FPN* expression in tissues. **h,** *LCN2* expression in tissues. RQ, relative quantification. Data are presented as mean ± SD. For e, f, g, h, n = 6 mice. The statistical significance are analyzed by a two-tailed Student's t-test in b and d and by one-way ANOVA with Tukey's test in e, f, g, h.

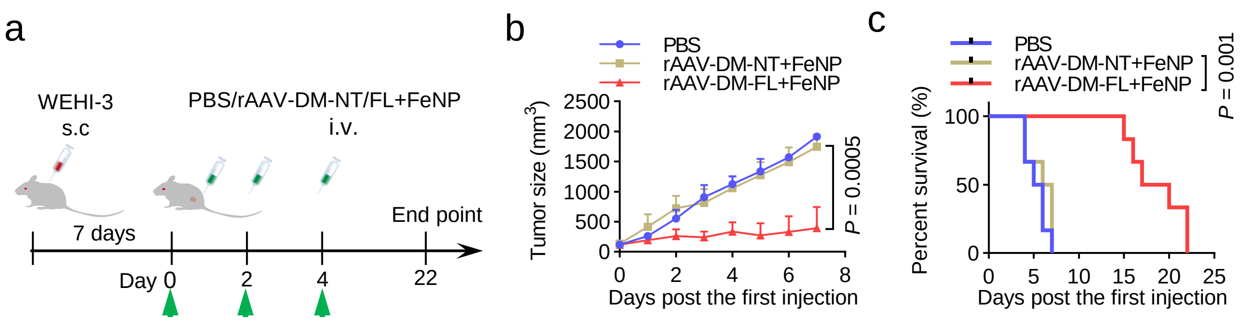

Supplementary Figure 49. The in vivo antitumor effects of virus-based GIFT in WEHI-3 xenografted mice. Mice were treated by three times intravenous injection of PBS, rAAV-DM-NT+FeNP (1×10^{10} vg/mouse plus 3 mg/kg FeNP), and rAAV-DM-FL+FeNP (1×10^{10} vg/mouse plus 3 mg/kg FeNP). **a,** Schematics of animal treatment. s.c., subcutaneous injection; i.v., intravenous injection. **b,** Tumor growth curve. Data are presented as mean ±SD ($n = 6$ mice) analyzed by one-way ANOVA with Tukey's test. **c,** Kaplan–Meier survival curves (statistical significance is analyzed by the log-rank test, n = 6 mice).

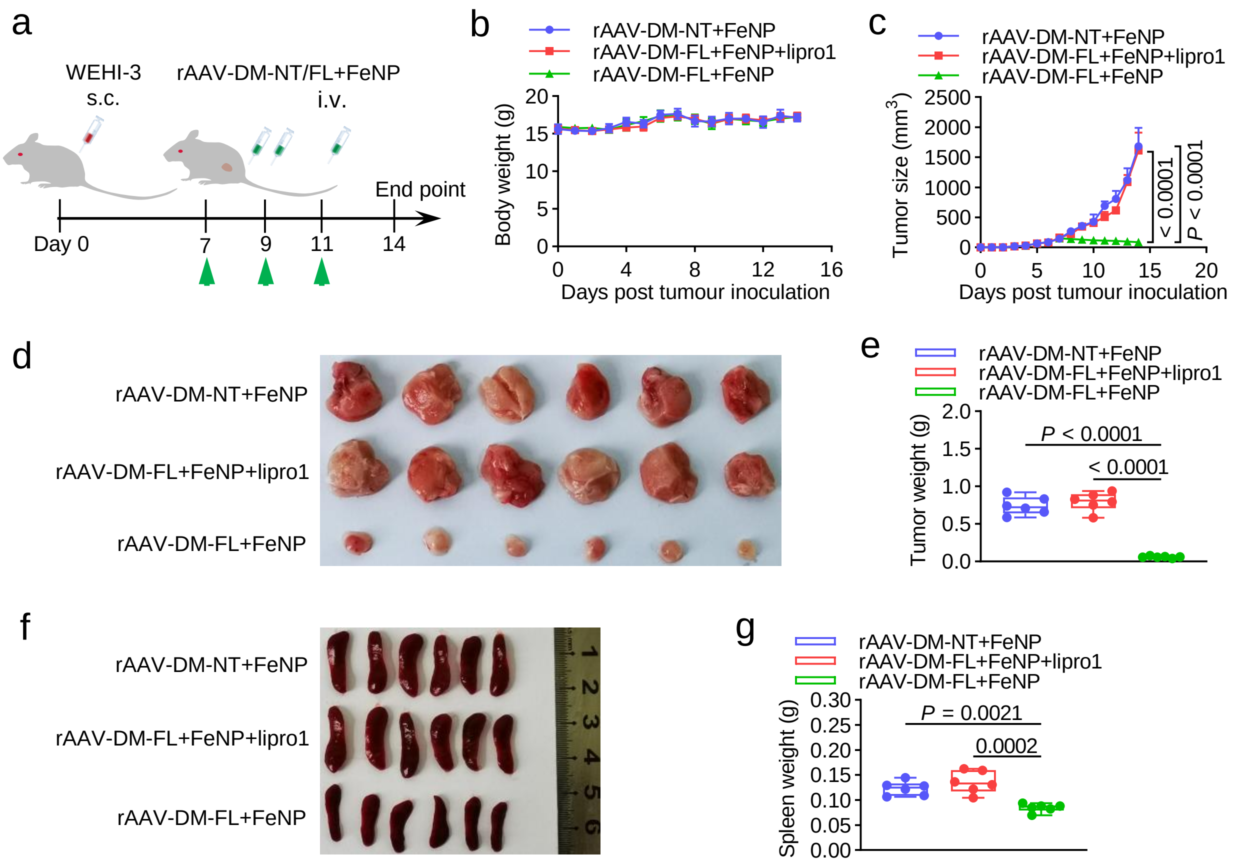

Supplementary Fig. 50. The in vivo antitumor effects of GIFT can be reverted by Liproxstatin-1 in WEHI-3 xenograft mice. The WEHI-3-tumor-bearing mice was constructed by subcutaneously injecting the WEHI-3 cells. The tumour-bearing mice administered with GIFT reagents were also daily intraperitoneal (i.p.) injected with liproxstatin-1 (10 mg/kg) for 7 days. **a,** Schematics of animal treatment. s.c., subcutaneous injection; i.v., intravenous injection. **b**, Average body weight. **c,** Tumor growth curve. **d**, Tumor imaging. **e**, Tumor weight. **f**, Spleen imaging. **g**, Spleen weight. The box-whisker plots in e and g show median (middle line), the 25th and 75th percentile (box) and min to max (whiskers). Other data are presented as mean ± SD ($n = 6$ mice). The statistical significance is analyzed by one-way ANOVA with Tukey's test in c, e, and g.

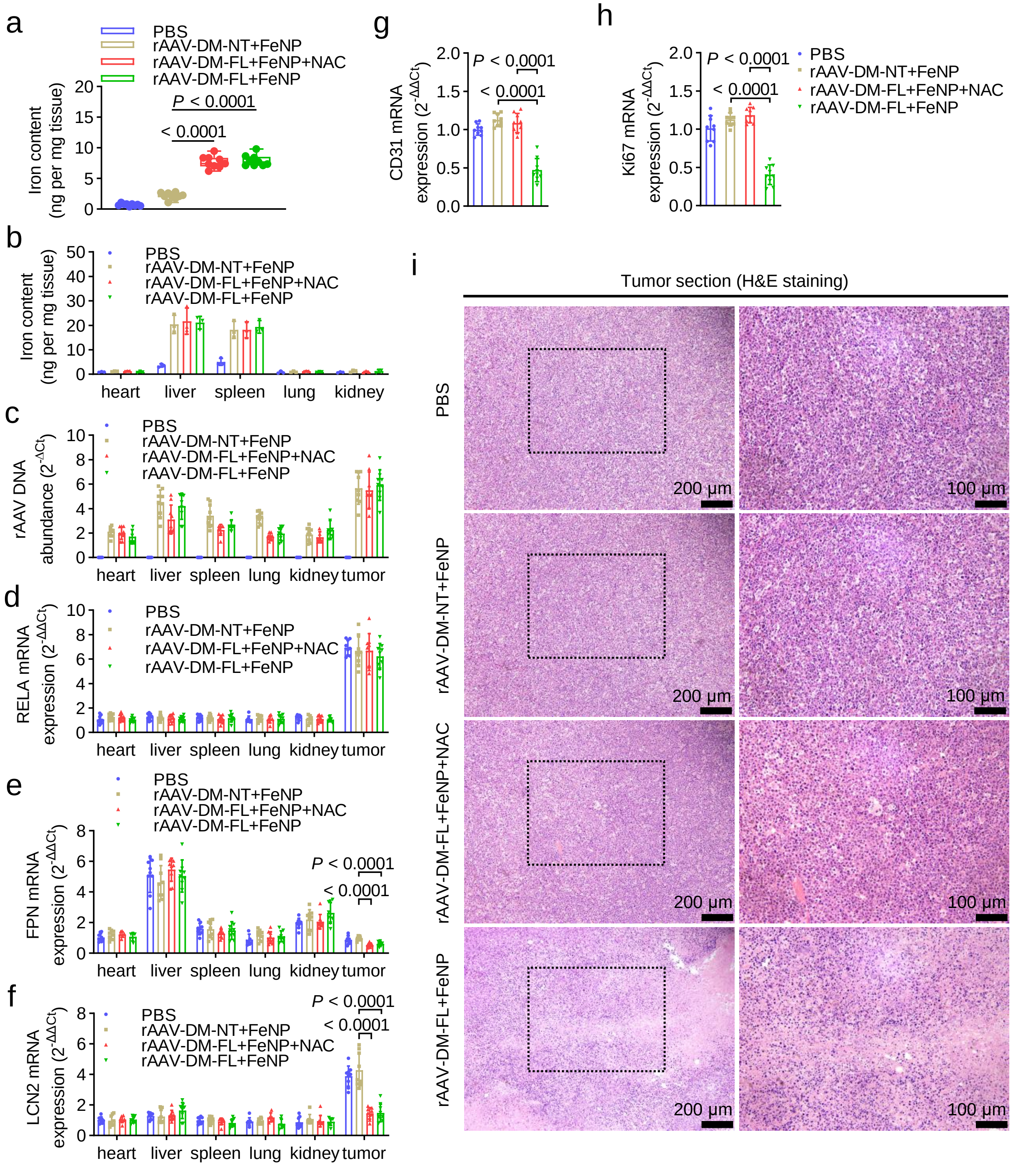

Supplementary Figure 51. **The in vivo antitumor effects of GIFT in the WEHI-3 xenograft mice (Figure 5). a,** Iron content in tumors of mice in different treatment groups. In box-whisker plots, median (middle line), 25th, 75th percentile (box) and min to max (whiskers).**b,** Iron content in major organs of mice in different treatment groups. **c**, Abundance of virus DNA in tissues. **d,** *RELA* expression in tissues. **e,** *FPN* expression in tissues. **f,** *LCN2* expression in tissues. **g,** *CD31* expression in tumors. h, *Ki67* expression in tumors. **i,** Representative H&E-stained sections of tumors. Data are presented as mean ± SD. For a, c, d, e, f, g and h, *n* = 9 mice; for b, *n* = 3 mice. The statistical significance is analyzed by one-way ANOVA with Tukey's test.

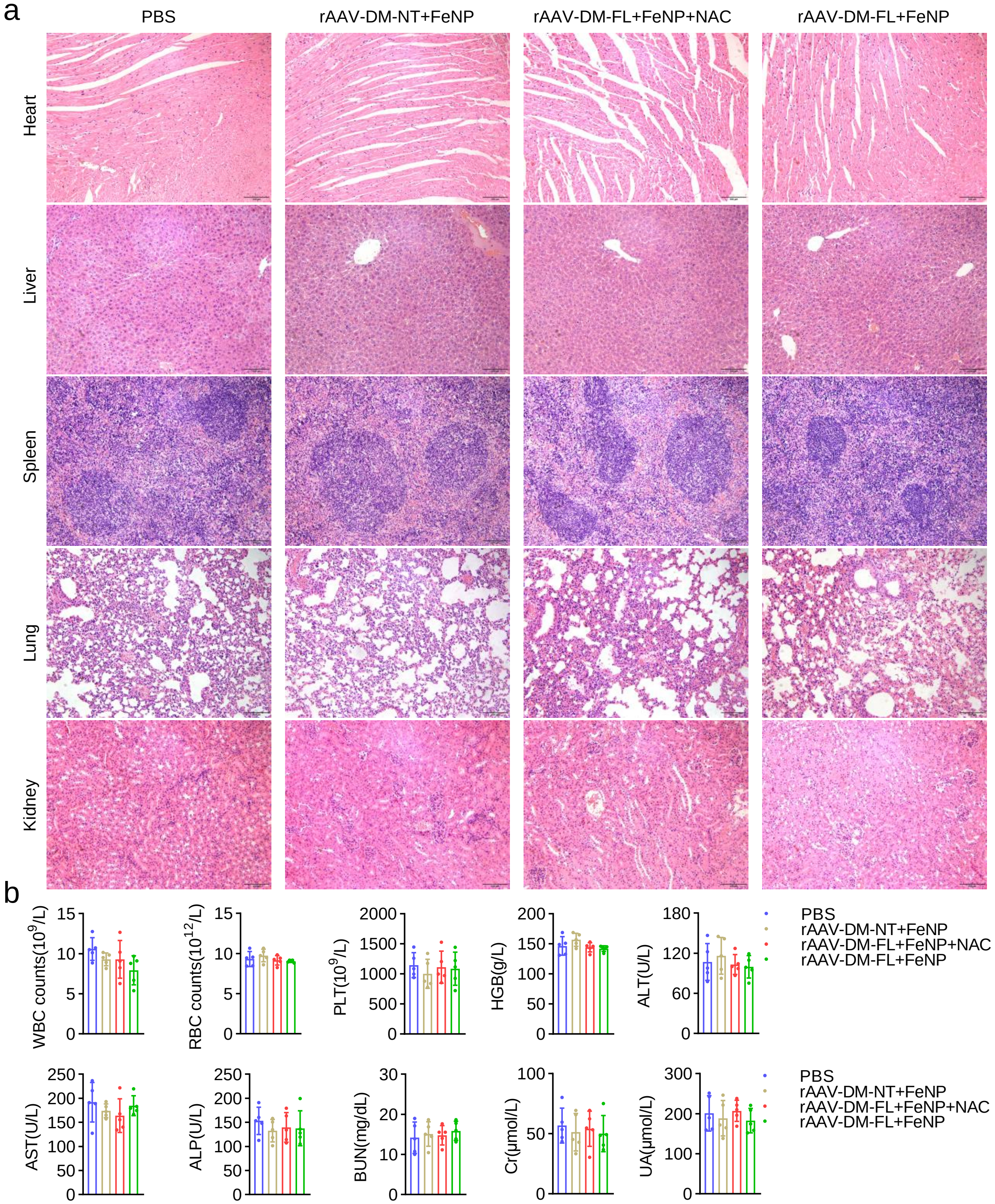

Supplementary Figure 52. **The in vivo antitumor effects of GIFT in the WEHI-3 xenografted mice (Figure 5).** Safety evaluations. **a,** H&E-stained sections of major organs. Scale bars, 100 μm. **b,** Routine blood test and serum biochemical parameter detection. Data are presented as mean ± SD (*n* = 5 mice).

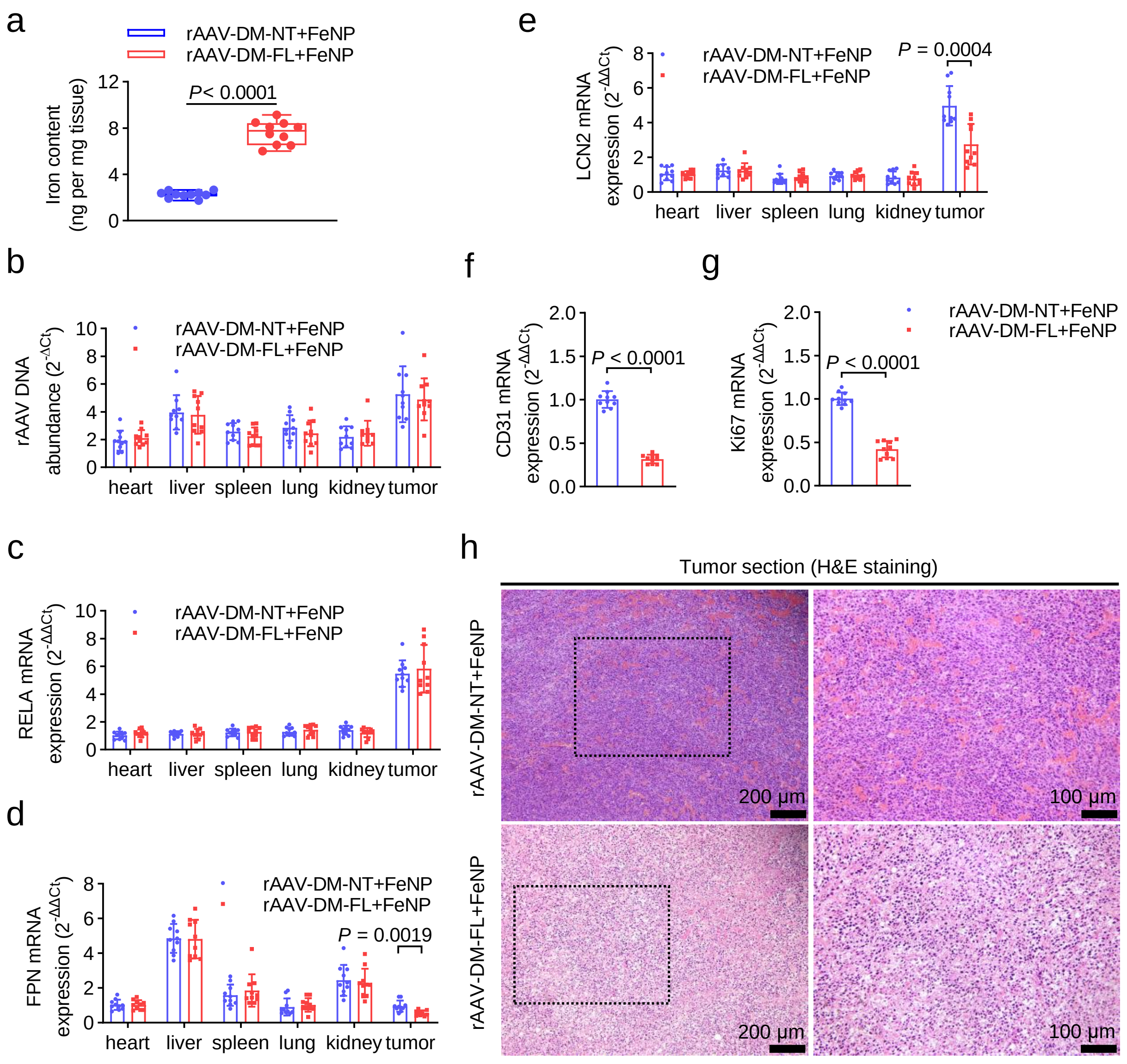

Supplementary Figure 53. **The in vivo antitumor effects of GIFT in the CT-26 xenografted mice (Figure 6). a,** Iron content in tumors of mice in different treatment groups (n = 10 mice). The box-whisker plot shows median (middle line), the 25th and 75th percentile (box) and min to max (whiskers). **b,** Abundance of virus DNA in tissues. **c,** *RELA* expression in tissues. **d,** *FPN* expression in tissues. **e,** *LCN2* expression in tissues. **f,** *CD31* expression in tumors. **g,** *Ki67* expression in tumors. **h,** Representative H&E-stained sections of tumors. Data are presented as mean ± SD (n = 10 mice) and analyzed by a two-tailed Student's t-test.

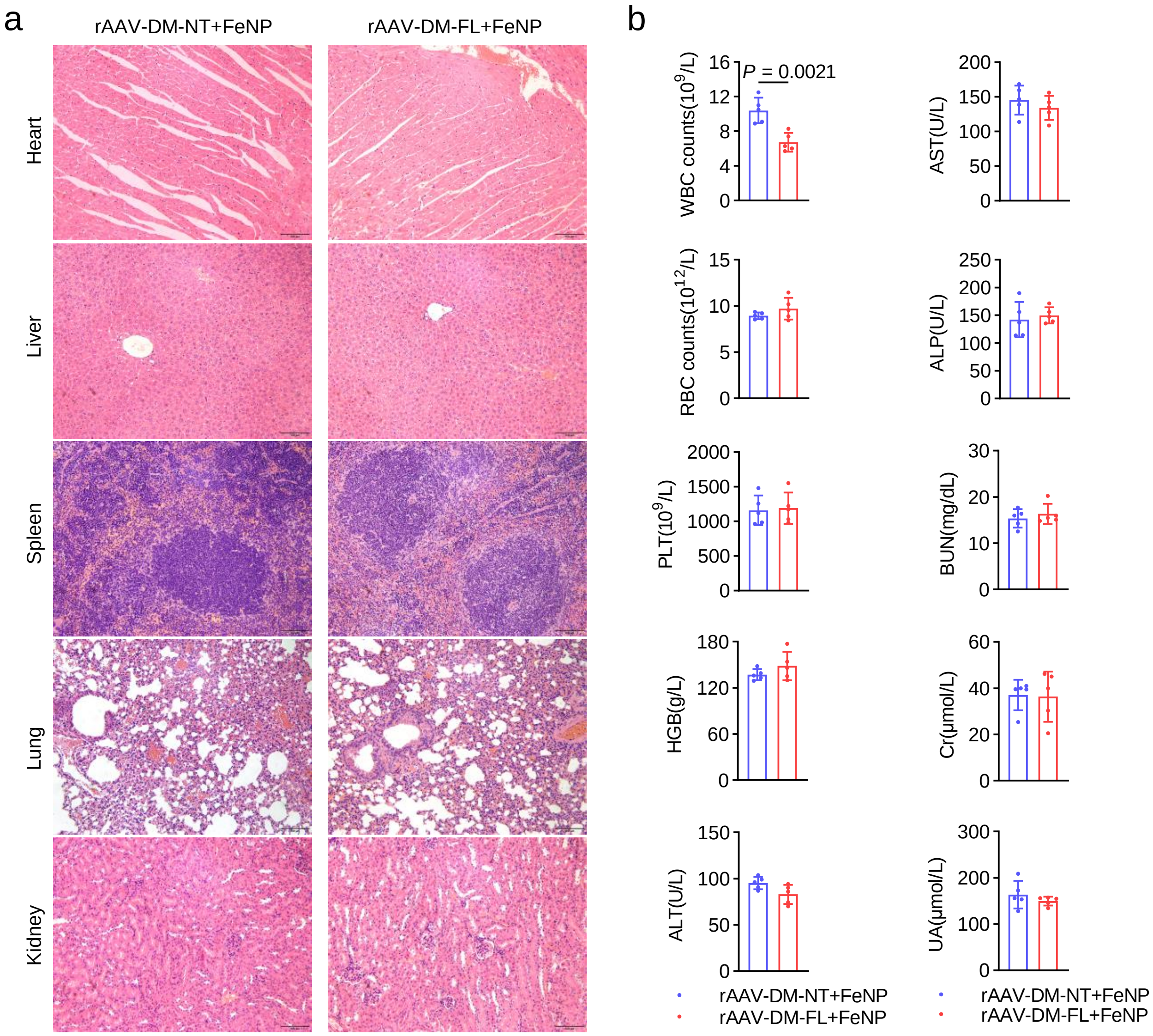

Supplementary Figure 54. **The in vivo antitumor effects of GIFT in the CT-26 xenografted mice (Figure 6).** Safety evaluations. **a,** Photographs of H&E-stained sections of major organs. Scale bars, 100 μm. **b,** Routine blood test and serum biochemical parameter detection. Data are presented as mean ± SD (*n* = 5 mice) and analyzed by a two-tailed Student's t-test.

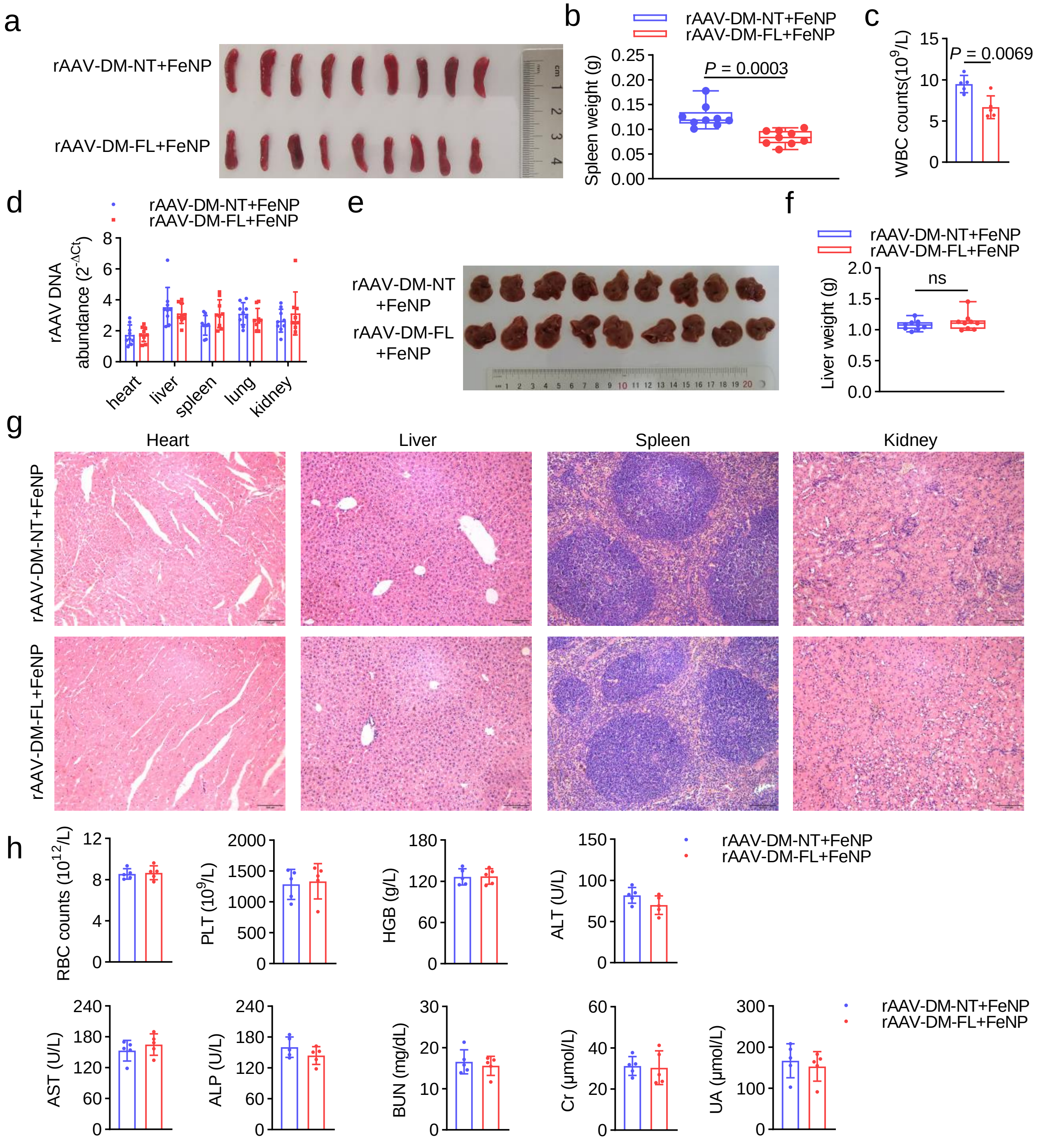

Supplementary Figure 55. The in vivo antitumor effects of GIFT in the pulmonary metastatic melanoma model (Figure 7). a, Photographic images of spleens. **b,** Spleen weight. **c,** WBC count. **d,** Abundance of virus DNA in tissues. **e,** Photographic images of livers. **f,** Liver weight. **g,** Photographs of H&E-stained sections of major organs. Scale bars, 100 μm. **h,** Routine blood test and serum biochemical parameter detection. The box-whisker plots in b and f show median (middle line), the 25th and 75th percentile (box) and min to max (whiskers) (n = 9 mice). For c and d, data are presented as mean ± SD (n = 9 mice); for h, data are presented as mean ± SD (n = 5 mice). ns, no significant difference. The statistical significance is analyzed by a two-tailed Student's t-test.

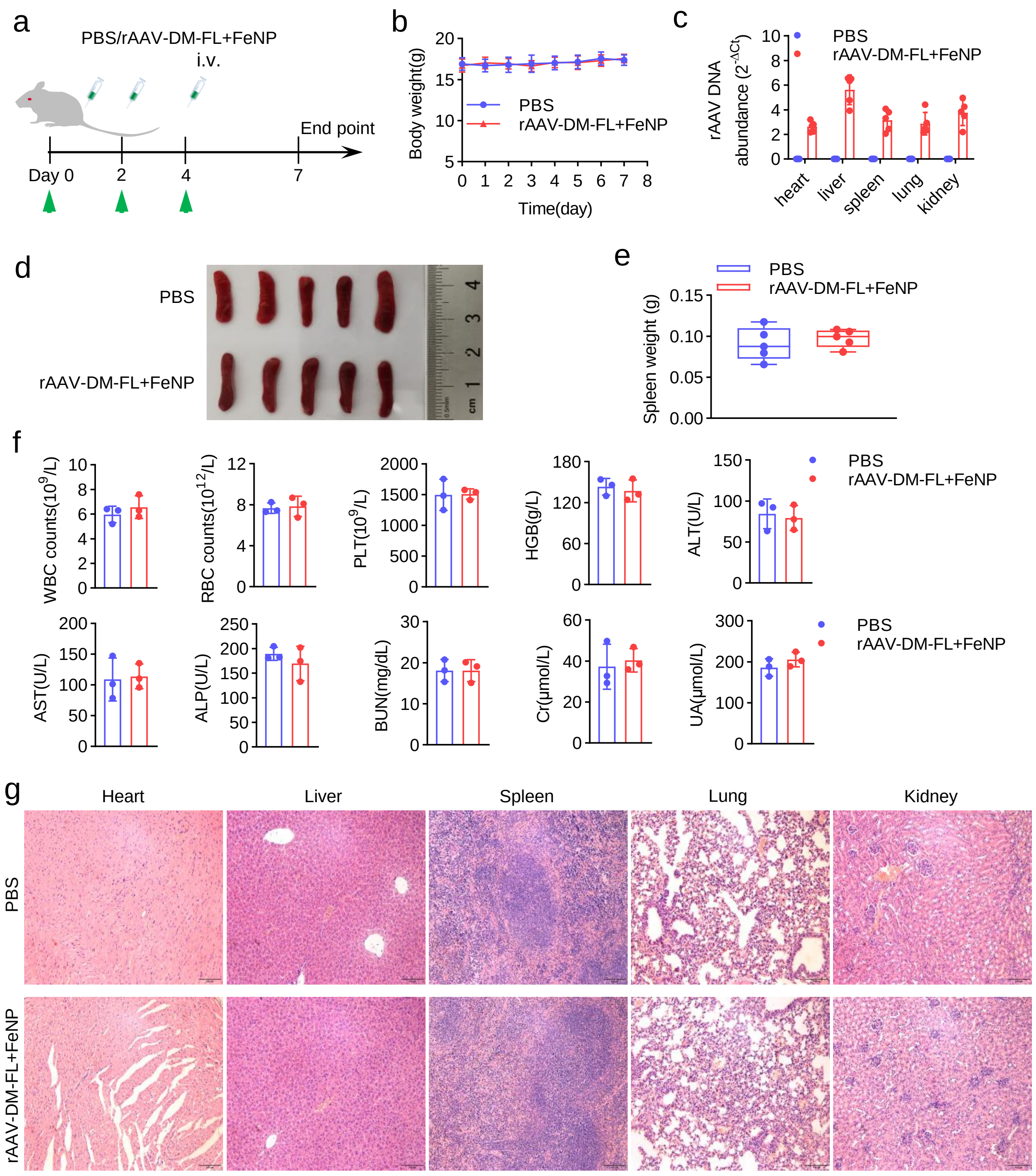

Supplementary Figure 56. Safety evaluations of the GIFT. a, Schematic illustration for the GIFT treatment. **b,** Average body weight of all mice in different treatment groups. **c,** Abundance of virus DNA in tissues. **d,** Photographic images of spleens. **e,** Spleen weight. The box-whisker plot shows median (middle line), the 25th and 75th percentile (box) and min to max (whiskers). **f,** Routine blood test and serum biochemical parameter detection. **g,** Photographs of H&E-stained sections of major organs. Scale bars, 100 μm. Data are presented as mean ± SD. For b, c, and e, *n* = 5 mice; for f, *n* = 3 mice.

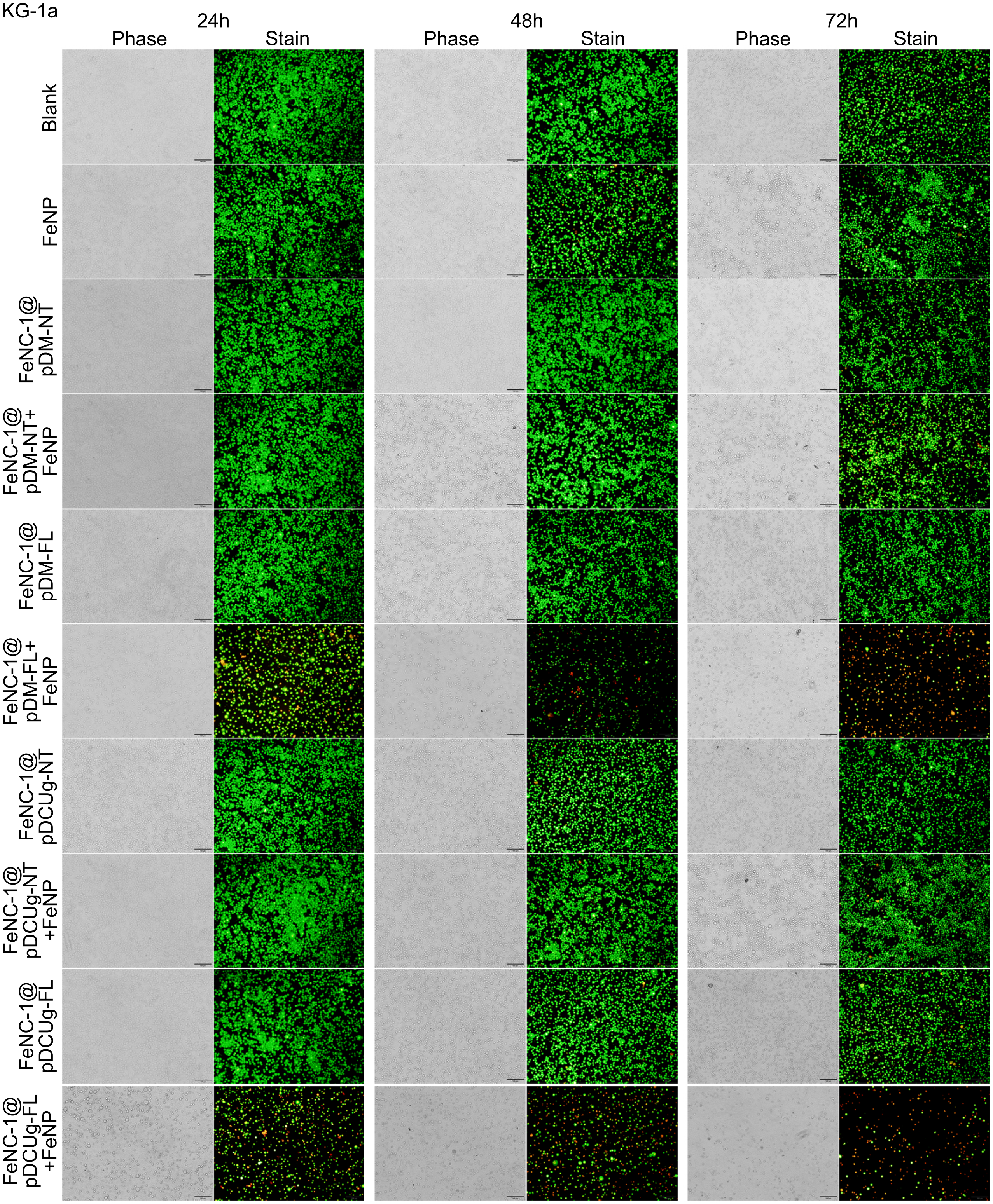

Supplementary Figure 57. Transfection of KG-1a cells with various plasmids by Fe nanocarrier (FeNC). Cells (1×10^5) were seeded into 24-well plates and incubated overnight. Cells were treated with FeNC (0.5 µg) and 500 ng of various plasmids according to the manufacturer's instructions. The transfected cells were cultured for 24 h and then incubated with or without 50 µg/mL of FeNP. Cells were cultured for another 72 h. At 24 h, 48 h and 72 h post FeNP administration, cells were stained with acridine orange and ethidium bromide and imaged under a fluorescence microscope. Scale bars, 100 µm. Representative data of two independent experiments.

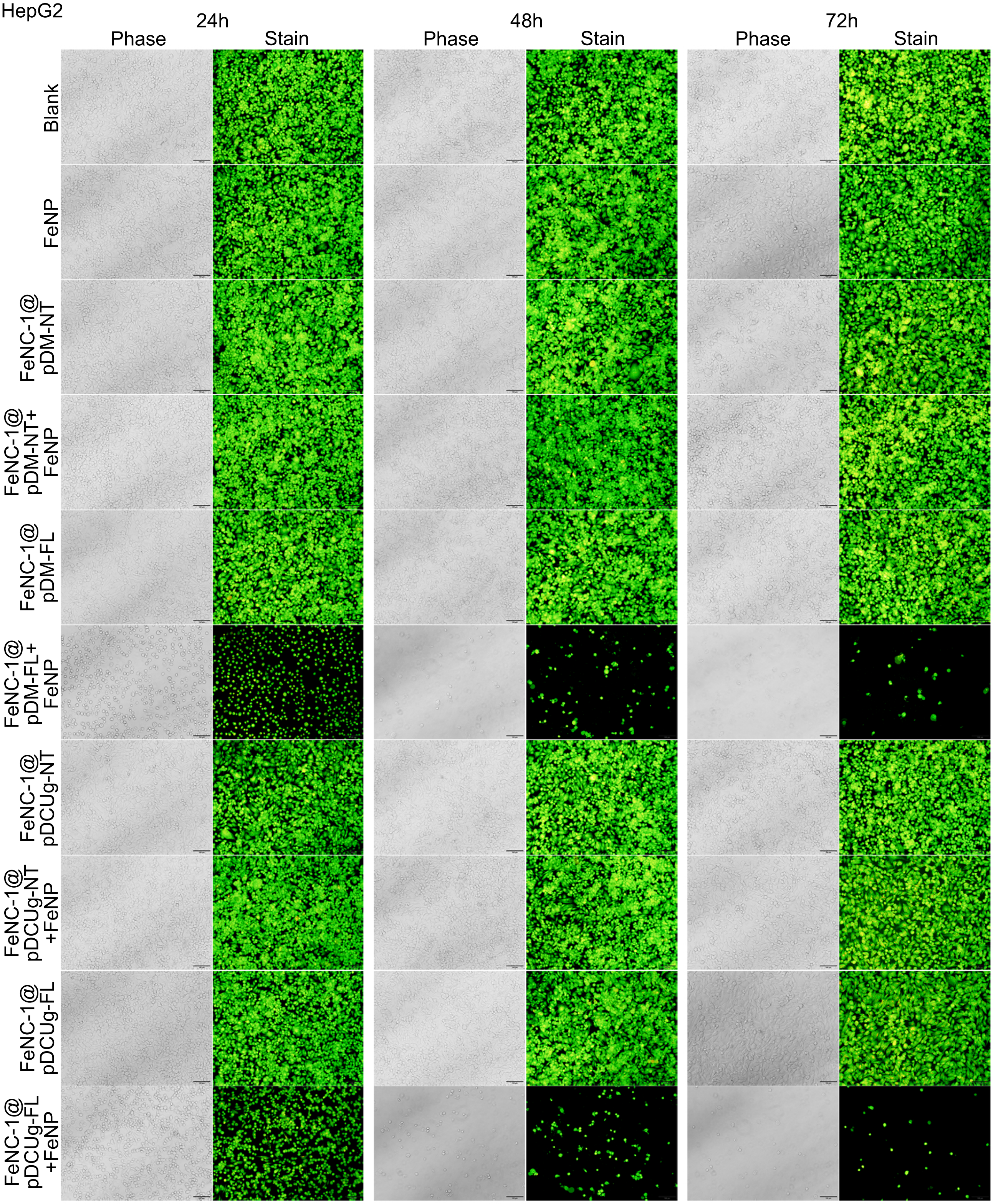

Supplementary Figure 58. Transfection of HepG2 cells with various plasmids by Fe nanocarrier (FeNC). Cells (1×10^5) were seeded into 24-well plates overnight before transfection. Cells were treated with FeNC (0.5 μg) and 500 ng of various plasmids according to the manufacturer's instructions. The transfected cells were cultured for 24 h and then incubated with or without 50 μ g/mL FeNP, and cells were cultured for another 72 h. At 24 h, 48 h and 72 h post FeNP administra-tion, cells were stained with acridine orange and ethidium bromide and imaged under a fluores-cence microscope. Scale bars, 100 μm. Representative data of two independent experiments.

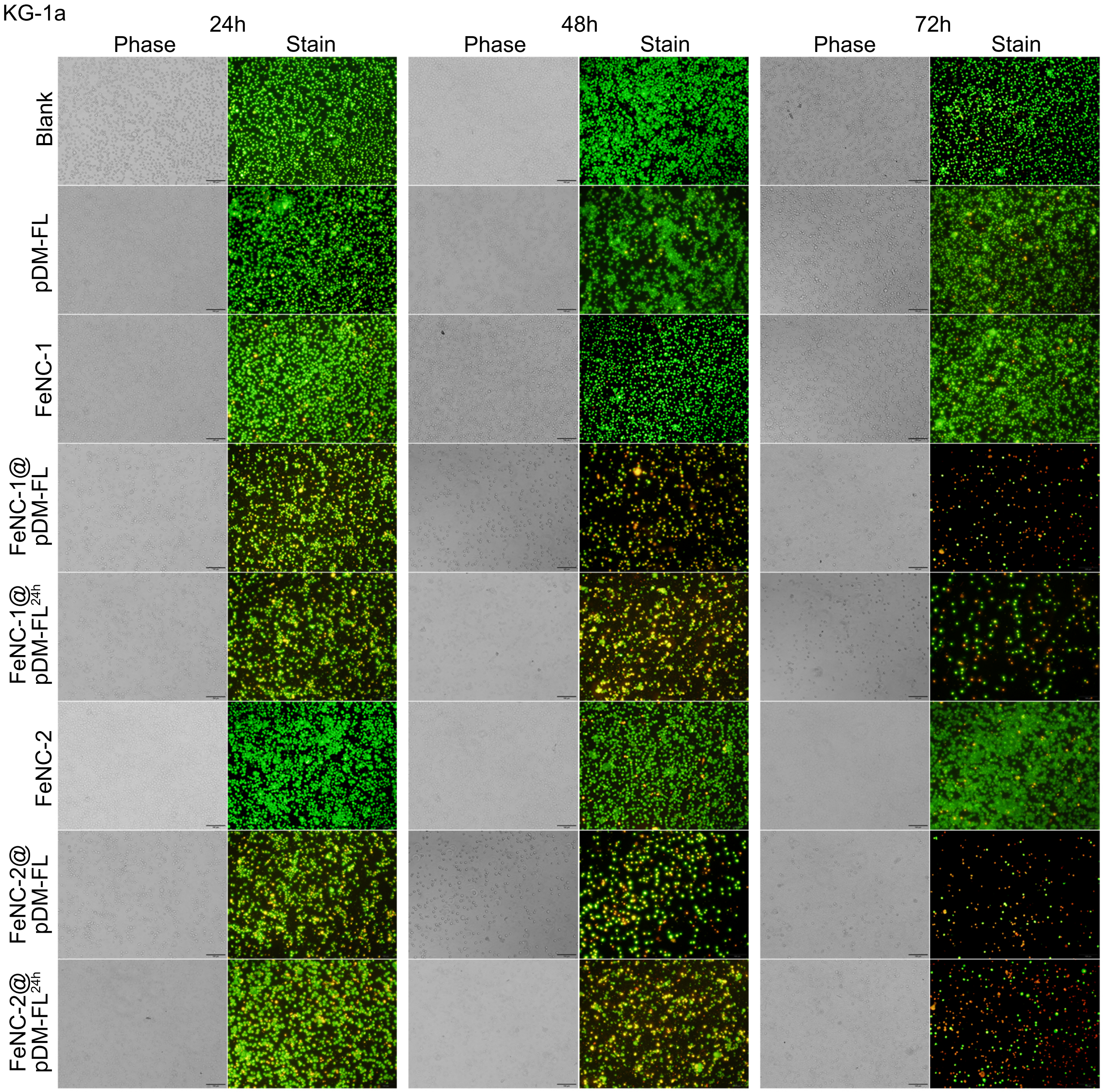

Supplementary Figure 59. Transfection of KG-1a cells with various plasmids by two Fe nanocarrier (FeNC). Cells (1×10^5) were seeded into 24-well plates overnight before transfec-tion. Cells were treated with various plasmids and 50 μg/mL FeNC (FeNC-1 and FeNC-2), respectively. All cells were cultured for another 72 h. At 24 h, 48 h and 72 h post FeNC admin-istration, cells were stained with acridine orange and ethidium bromide and imaged under a fluorescence microscope. Scale bars, 100 μm. FeNC-1/FeNC-2@pDM-FL, plasmid and FeNC were mixed and directly added to cells; FeNC-1/FeNC-2@pDM-FL24h, plasmid and FeNC were mixed and kept for 24 h, and then added to cells. Representative data of two independent experiments.

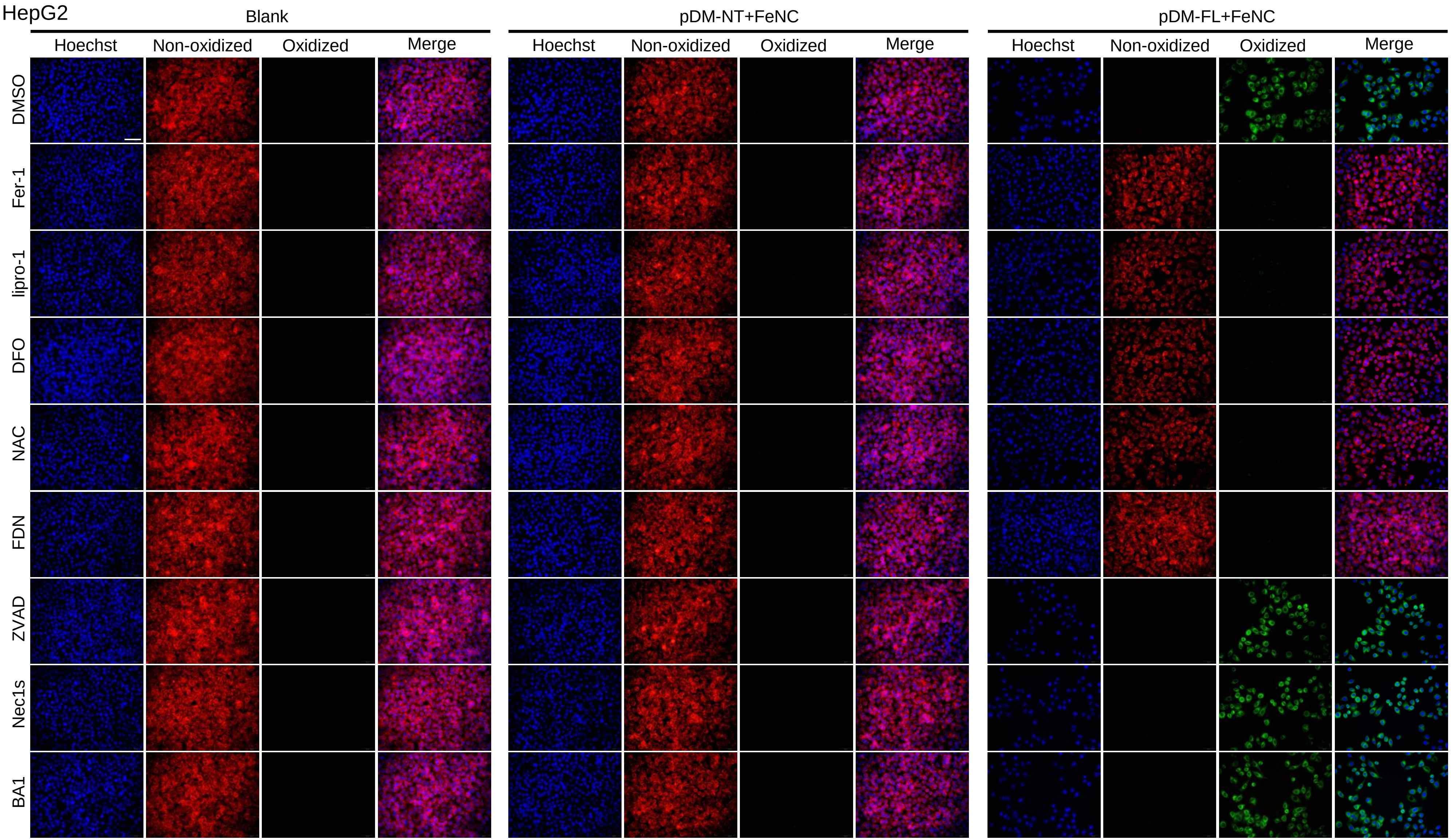

Supplementary Figure 60. **Detection of lipid ROS. Representative images of HepG2 cells stained with C11-BODIPY.** Scale bars, 50 µm. Representative data of two independent experiments. Transfection of HepG2 cells with various plasmids by Fe nanocarrier (FeNC). Cells (1×10^5) were seeded into 24-well plates overnight before transfection. Cells were treated with 50 µg/mL of FeNC and various plasmids and cultured for 24 h. The treated cells were then co-incubated with the indicated inhibitors for 48 h. Fer1, Ferrostatin-1 (1 µM); lipro1, liproxstatin-1 (1 µM); DFO, deferoxamine (100 µM); NAC, N-acetylcysteine (1 mM); ZVAD, ZVAD-FMK (50 µM); Nec1s, Necrostatin-1s (10 µM); BA1, Bafilomycin A1 (1 nM).

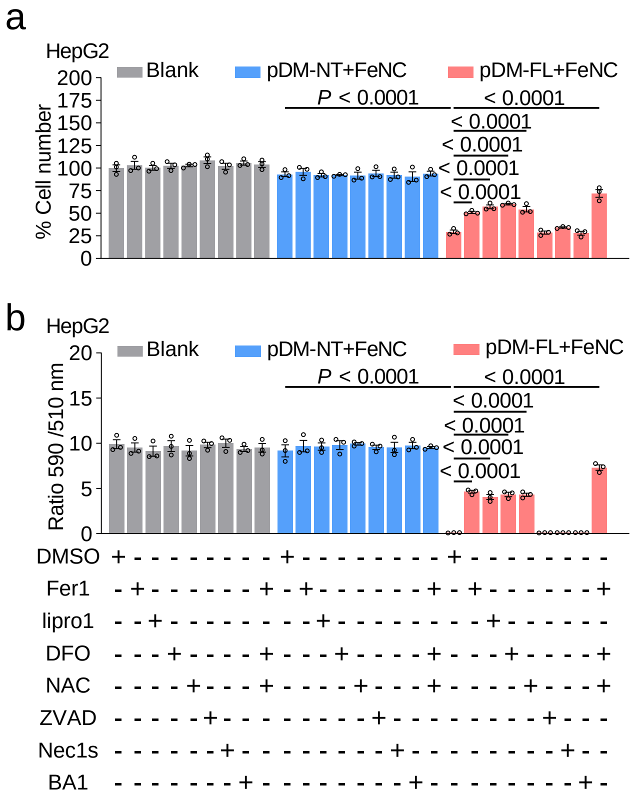

Supplementary Figure 61. **Transfection of HepG2 cells with various plasmids by Fe nanocarrier (FeNC).** Cells (1×10^5) were seeded into 24-well plates overnight before transfection. Cells were treated with 50 µg/mL of FeNC and various plasmids and cultured for 24 h. The treated cells were then co-incubated indicated inhibitors for 48h. **a**, Cell viability detected by the Cell-Titer-Glo 2.0 reagent (*n* = 3 biologically independent samples). **b**, Quantified results of lipid peroxidation in Supplementary Figure 61 (*n* = 3 independent micrographs). The lipid peroxidation in cells were determined by quantitating the fluorescence intensities analyzed by ImageJ software and calculating the ratio of intensity in 590 to 510 channels. Data are presented as mean ± SD and analyzed by one-way ANOVA with Tukey's test. Fer1, Ferrostatin-1(1 µM); lipro1, liproxstatin-1(1 µM); DFO, deferoxamine (100 µM); NAC, N-acetylcysteine (1 mM); ZVAD, ZVAD-FMK (50 µM); Nec1s, Necrostatin-1s (10 µM); BA1, Bafilomycin A1 (1 nM).

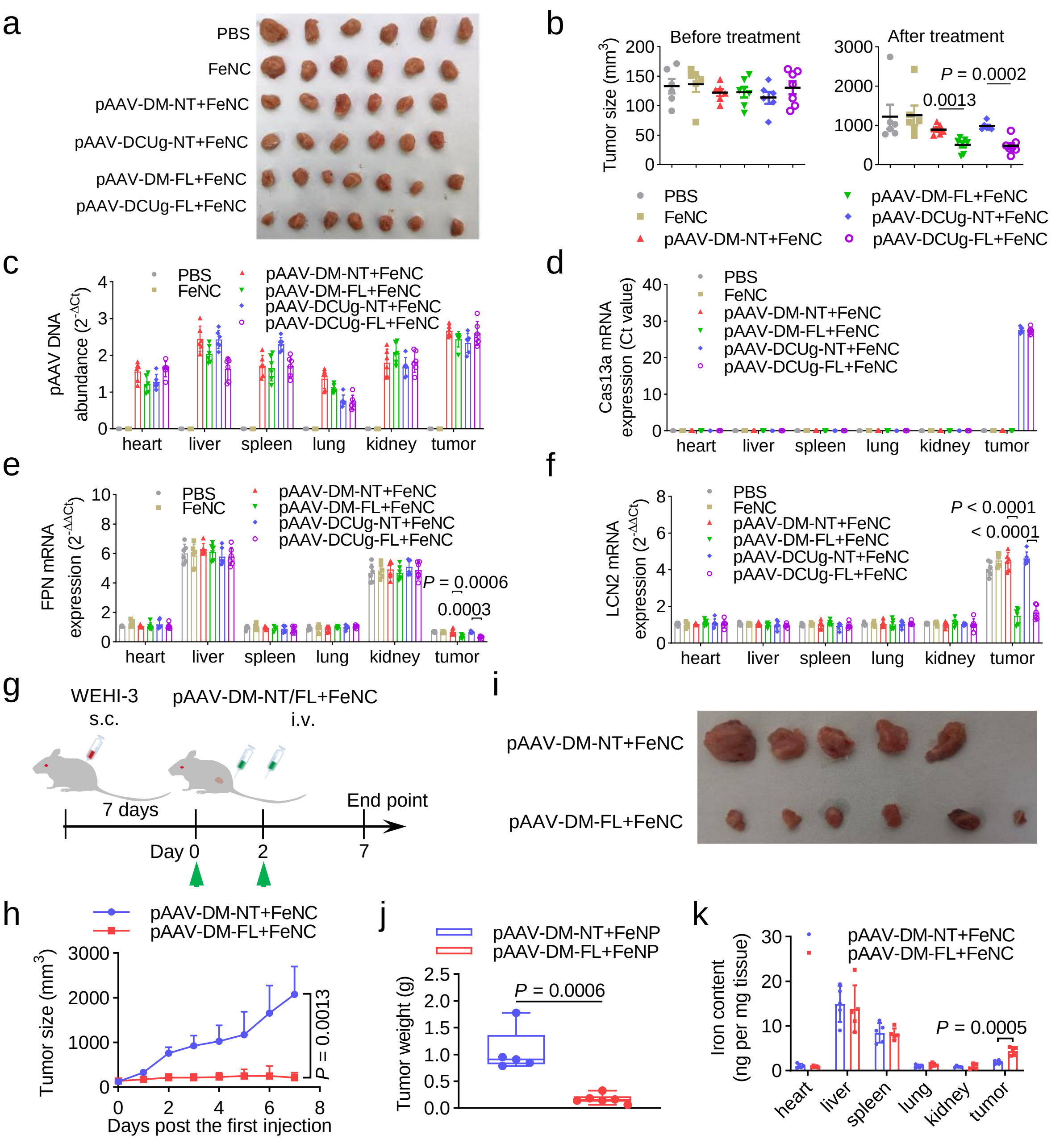

Supplementary Figure 62. The in vivo antitumor effects of FeNC-based GIFT. a–f, tumor inhibition assay with single time drug administration. **a,** Tumors of mice treated by FeNC@DNA. s.c., subcutaneous injection; i.v., intravenous injection. **b,** Tumor volumes before and after treatment (for pAAV-DM-FL+FeNC and pAAV-DCUg-FL+FeNC groups, *n* = 7 mice; for other groups, *n* = 6 mice). **c,** Abundance of plasmid DNA in tissues. **d,** Cas13a expression in tissues. **e,** *FPN* expression in tissues. **f,** *LCN2* expression in tissues. RQ, relative quantification. For c, d, e, f, *n* = 6 mice. Data are presented as mean ± SD and analyzed by one-way ANOVA with Tukey's test. **g–j,** tumor inhibition assay with two times drug administration. **g,** Schematics of animal treatment. **h,** Tumor growth curve. **i,** Tumor imaging. **j,** Tumor weight. The box-whisker plot shows median (middle line), the 25th and 75th percentile (box) and min to max (whiskers). **k**, Iron content in tissues of mice in different treatment groups. For i, h, j, *n* = 5 mice in pAAV-DM-NT+FeNC group and *n* = 6 in pAAV-DM-FL+FeNC group; for k, *n*=5 mice. Data are mean± SD. The statistical significance are analyzed by one-way ANOVA with Tukey's test in b, e, f and by a two-tailed Student's t-test in h, j, k.

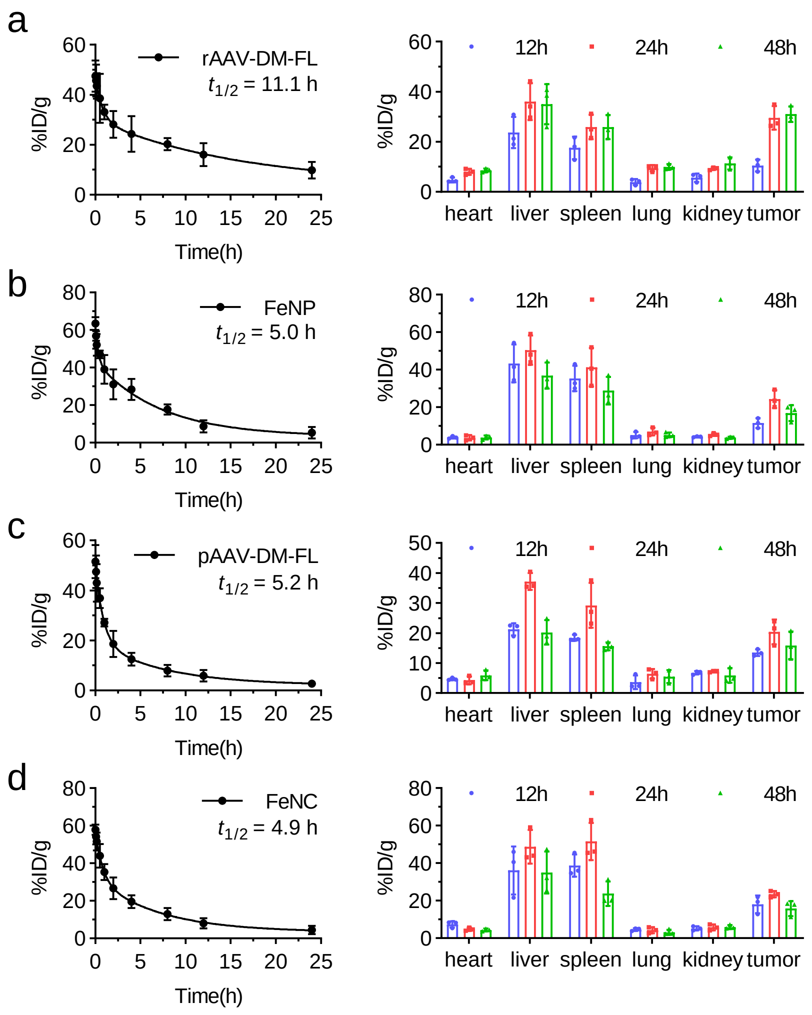

Supplementary Fig. 63. Pharmacokinetic and biodistribution of rAAV, FeNP, pAAV and FeNC. For pharmacokinetics study, healthy mice were intravenously injected with rAAV-DM-FL+FeNP and pAAV-DM-FL+FeNC, respectively. The blood was collected at various time (1 min, 5 min, 10 min, 0.5 h, 1 h, 2 h, 4 h, 8 h, 12 h, and 24 h) after injection. For biodistribution study, WEHI-3-tumor-bearing mice were intravenously administered with rAAV-DM-FL+FeNP and pAAV-DM-FL+FeNC, respectively. Mice were sacrificed, and the tumors and major organs were harvested at predesignated time points. The content of iron and rAAV/pAAV in tissues (blood, tumors and organs) were measured by ICP-MS and qPCR, respectively. **a,** Pharmacokinetic and biodistribution of rAAV-DM-FL. **b**, Pharmacokinetic and biodistribution of FeNP. **c,** Pharmacokinetic and biodistribution of pAAV-DM-FL. **d**, Pharmacokinetic and biodistribution of FeNC. %ID/g, percentage injected dose per gram. Data are presented as mean ± SD (*n* = 3 biologically independent samples).

Plasmids and the functional sequences

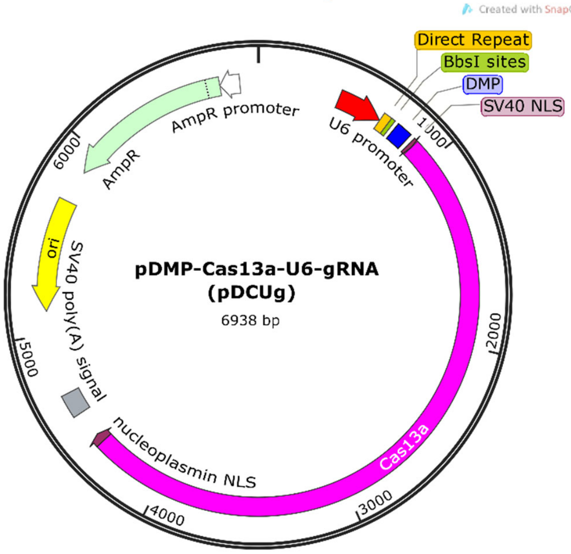

pDMP-Cas13a-U6-gRNA
U6+Direct repeat+Spacer+DMP+Cas13a+SV40 poly(A) signal
GAGGGCCTATTTCCCATGATTCCTTCATATTTGCATATACGATACAAGGCTGTTAGAGAGATAATT
GGAATTAATTTGACTGTAAACACAAAGATATTAGTACAAAATACGTGACGTAGAAAGTAATAAT
TTCTTGGGTAGTTTGCAGTTTTAAAATTATGTTTTAAAATGGACTATCATATGCTTACCGTAACTT
GAAAGTATTTCGATTTCTTGGCTTTATATATCTTGTGGAAAGGACGAAACACCGATTTAGACTAC
CCCAAAAACGAAGGGGACTAAAACGGGTCTTCGAGAAGACCTGGATCCGGCCTAACTGGCCG
GTACCGGGAATTTCCGGGGACTTTCCGGGAATTTCCGGGGACTTTCCGGGAATTTCCTAGAGG
GTATATAATGGAAGCTCGACTTCCAGGTCGACGCGTAAGCTTGCCACCATGCCAAAGAAGAAG
CGGAAGGTCGGTATCCACGGAGTCCCAGCAGCCATGAAAGTGACCAAGGTCGACGGCATCAG
CCACAAGAAGTACATCGAAGAGGGCAAGCTCGTGAAGTCCACCAGCGAGGAAAACCGGACC
AGCGAGAGACTGAGCGAGCTGCTGAGCATCCGGCTGGACATCTACATCAAGAACCCCGACAA
CGCCTCCGAGGAAGAGAACCGGATCAGAAGAGAGAACCTGAAGAAGTTCTTTAGCAACAAGG
TGCTGCACCTGAAGGACAGCGTGCTGTATCTGAAGAACCGGAAAGAAAAGAACGCCGTGCAG
GACAAGAACTATAGCGAAGAGGACATCAGCGAGTACGACCTGAAAAACAAGAACAGCTTCTC
CGTGCTGAAGAAGATCCTGCTGAACGAGGACGTGAACTCTGAGGAACTGGAAATCTTTCGGA
AGGACGTGGAAGCCAAGCTGAACAAGATCAACAGCCTGAAGTACAGCTTCGAAGAGAACAA
GGCCAACTACCAGAAGATCAACGAGAACAACGTGGAAAAAGTGGGCGGCAAGAGCAAGCGG
AACATCATCTACGACTACTACAGAGAGAGCGCCAAGCGCAACGACTACATCAACAACGTGCAG
GAAGCCTTCGACAAGCTGTATAAGAAAGAGGATATCGAGAAACTGTTTTTCCTGATCGAGAAC
AGCAAGAAGCACGAGAAGTACAAGATCCGCGAGTACTATCACAAGATCATCGGCCGGAAGAA
CGACAAAGAGAACTTCGCCAAGATTATCTACGAAGAGATCCAGAACGTGAACAACATCAAAG
AGCTGATTGAGAAGATCCCCGACATGTCTGAGCTGAAGAAAAGCCAGGTGTTCTACAAGTACT
ACCTGGACAAAGAGGAACTGAACGACAAGAATATTAAGTACGCCTTCTGCCACTTCGTGGAAA
TCGAGATGTCCCAGCTGCTGAAAAACTACGTGTACAAGCGGCTGAGCAACATCAGCAACGATA
AGATCAAGCGGATCTTCGAGTACCAGAATCTGAAAAAGCTGATCGAAAACAAACTGCTGAAC
AAGCTGGACACCTACGTGCGGAACTGCGGCAAGTACAACTACTATCTGCAAGTGGGCGAGATC
GCCACCTCCGACTTTATCGCCCGGAACCGGCAGAACGAGGCCTTCCTGAGAAACATCATCGGC
GTGTCCAGCGTGGCCTACTTCAGCCTGAGGAACATCCTGGAAACCGAGAACGAGAACGGTATC
ACCGGCCGGATGCGGGGCAAGACCGTGAAGAACAACAAGGGCGAAGAGAAATACGTGTCCG
GCGAGGTGGACAAGATCTACAATGAGAACAAGCAGAACGAAGTGAAAGAAAATCTGAAGATG
TTCTACAGCTACGACTTCAACATGGACAACAAGAACGAGATCGAGGACTTCTTCGCCAACATC
GACGAGGCCATCAGCAGCATCAGACACGGCATCGTGCACTTCAACCTGGAACTGGAAGGCAA
GGACATCTTCGCCTTCAAGAATATCGCCCCCAGCGAGATCTCCAAGAAGATGTTTCAGAACGA
AATCAACGAAAAGAAGCTGAAGCTGAAAATCTTCAAGCAGCTGAACAGCGCCAACGTGTTCA
ACTACTACGAGAAGGATGTGATCATCAAGTACCTGAAGAATACCAAGTTCAACTTCGTGAACA

AAAACATCCCCTTCGTGCCCAGCTTCACCAAGCTGTACAACAAGATTGAGGACCTGCGGAATA
CCCTGAAGTTTTTTGGAGCGTGCCCAAGGACAAAGAAGAGAAGGACGCCCAGATCTACCTG
CTGAAGAATATCTACTACGGCGAGTTCCTGAACAAGTTCGTGAAAAACTCCAAGGTGTTCTTTA
AGATCACCAATGAAGTGATCAAGATTAACAAGCAGCGGAACCAGAAAACCGGCCACTACAAG
TATCAGAAGTTCGAGAACATCGAGAAAACCGTGCCCGTGGAATACCTGGCCATCATCCAGAGC
AGAGAGATGATCAACAACCAGGACAAAGAGGAAAAGAATACCTACATCGACTTTATTCAGCAG
ATTTTCCTGAAGGGCTTCATCGACTACCTGAACAAGAACAATCTGAAGTATATCGAGAGCAAC
AACAACAATGACAACAACGACATCTTCTCCAAGATCAAGATCAAAAAGGATAACAAAGAGAA
GTACGACAAGATCCTGAAGAACTATGAGAAGCACAATCGGAACAAAGAAATCCCTCACGAGA
TCAATGAGTTCGTGCGCGAGATCAAGCTGGGGAAGATTCTGAAGTACACCGAGAATCTGAACA
TGTTTTACCTGATCCTGAAGCTGCTGAACCACAAAGAGCTGACCAACCTGAAGGGCAGCCTGG
AAAAGTACCAGTCCGCCAACAAAGAAGAAACCTTCAGCGACGAGTTGGAACTGATCAACCTG
CTGAACCTGGACAACAACAGAGTGACCGAGGACTTCGAGCTGGAAGCCAACGAGATCGGCA
AGTTCCTGGACTTCAACGAAAACAAAATCAAGGACCGGAAAGAGCTGAAAAAGTTCGACACC
AACAAGATCTATTTCGACGGCGAGAACATCATCAAGCACCGGGCCTTCTACAATATCAAGAAAT
ACGGCATGCTGAATCTGCTGGAAAAGATCGCCGATAAGGCCAAGTATAAGATCAGCCTGAAAG
AACTGAAAGAGTACAGCAACAAGAAGAATGAGATTGAAAAGAACTACACCATGCAGCAGAAC
CTGCACCGGAAGTACGCCAGACCCAAGAAGGACGAAAAGTTCAACGACGAGGACTACAAAG
AGTATGAGAAGGCCATCGGCAACATCCAGAAGTACACCCACCTGAAGAACAAGGTGGAATTC
AATGAGCTGAACCTGCTGCAGGGCCTGCTGCTGAAGATCCTGCACCGGCTCGTGGGCTACACC
AGCATCTGGGAGCGGGACCTGAGATTCCGGCTGAAGGGCGAGTTTCCCGAGAACCACTACATC
GAGGAAATTTTCAATTTCGACAACTCCAAGAATGTGAAGTACAAAAGCGGCCAGATCGTGGAA
AAGTATATCAACTTCTACAAAGAACTGTACAAGGACAATGTGGAAAAGCGGAGCATCTACTCC
GACAAGAAAGTGAAGAAACTGAAGCAGGAAAAAAAGGACCTGTACATCCGGAACTACATTGC
CCACTTCAACTACATCCCCCACGCCGAGATTAGCCTGCTGGAAGTGCTGGAAAACCTGCGGAA
GCTGCTGTCCTACGACCGGAAGCTGAAGAACGCCATCATGAAGTCCATCGTGGACATTCTGAA
AGAATACGGCTTCGTGGCCACCTTCAAGATCGGCGCTGACAAGAAGATCGAAATCCAGACCCT
GGAATCAGAGAAGATCGTGCACCTGAAGAATCTGAAGAAAAAGAAACTGATGACCGACCGGA
ACAGCGAGGAACTGTGCGAACTCGTGAAAGTCATGTTCGAGTACAAGGCCCTGGAAAAAAGG
CCGGCGGCCACGAAAAAGGCCGGCCAGGCAAAAAAGAAAAAGTGACTCGAGCACGTGCTAC
GAGATTTCGATTCCACCGCCGCCTTCTATGAAAGGTTGGGCTTCGGAATCGTTTTCCGGGACGC
CGGCTGGATGATCCTCCAGCGCGGGGATCTCATGCTGGAGTTCTTCGCCCACCCCAACTTGTTT
ATTGCAGCTTATAATGGTTACAAATAAAGCAATAGCATCACAAATTTCACAAATAAAGCATTTTT
TTCACTGCATTCTAGTTGTGGTTTGTCCAAACTCATCAATGTATCTTA

The spacer sequence in above skeleton vector can be replaced by the following sequences for constructing pDMP-Cas13a-U6-gRNAs targeting particular genes:
No transcript (NT): TAGATTGCTGTTCTACCAAGTAATCCAT
Human FPN guide: CACCGCAAAGTGCCACATCCGATCTCCC
Human LCN2 guide: TAACTCTTAATGTTGCCCAGCGTGAACT
Mouse FPN guide: TTATTCCAGTTATTGCTGATGCTCCCAT
Mouse LCN2 guide: TTGGTCGGTGGGGACAGAGAAGATGATG
Human FL:
CACCGCAAAGTGCCACATCCGATCTCCCGAAACACCGATTTAGACTACCCCAAAAACGAAGGG
GACTAAAACTAACTCTTAATGTTGCCCAGCGTGAACT
Mouse FL:
TTATTCCAGTTATTGCTGATGCTCCCATGAAACACCGATTTAGACTACCCCAAAAACGAAGGGG
ACTAAAACTTGGTCGGTGGGGACAGAGAAGATGATG

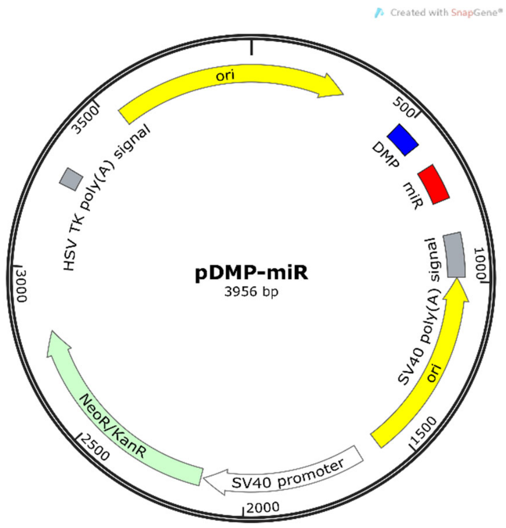

pDMP-miR
DMP+ miR +SV40 poly(A) signal

GGGAATTTCCGGGGACTTTCCGGGAATTTCCGGGGACTTTCCGGGAATTTCCTAGAGGGTATAT
AATGGAAGCTCGACTTCCAGGCTAGCGAATTCGCTAAGCACTTCGTGGCCGTCGATCGTTTAA
AGGGAGGTAGTGAGTCGACCAGTGGATCCTGGAGGCTTGCTGAAGGCTGTATGCTGGAGACG
CAGTGAGCCGAGATCGCGCCACCGCGTCTCGCAGGACACAAGGCCTGTTACTAGCACTCACAT
GGAACAAATGGCCCAGATCTGGCCGCACTCGAGATATCTAGACCCAGCTTTCTTGTACAAAGT
GGTTGATCTAGAGGGCCCGCGGTTCGCTGATGGGGGAGGCTAACTGAAACACGGAAGGAGAC
AATACCGGAAGGAACCCGCGCTATGACGGCAATAAAAAGACAGAATAAAACGCACGGGTGTT
GGGTCGTTTGTTCATAAACGCGGGGTTCGGTCCCAGGGCTGGCACTCTGTCGATACCCCACCG
TGACCCCATTGGGGCCAATACGCCCGCGTTTCTTCCTTTTCCCCACCCCACCCCCCAAGTTCGG
GTGAAGGCCCAGGGCTCGCAGCCAACGTCGGGGCGGCAGGCCCTGCCATAGGCGGCCGCGAC
TCTAGATCATAATCAGCCATACCACATTTGTAGAGGTTTTACTTGCTTTAAAAAACCTCCCACAC
CTCCCCCTGAACCTGAAACATAAAATGAATGCAATTGTTGTTGTTAACTTGTTTATTGCAGCTTA
TAATGGTTACAAATAAAGCAATAGCATCACAAATTTCACAAATAAAGCATTTTTTTCACTGCATT
CTAGTTGTGGTTTGTCCAAACTCATCAATGTATCTTA

The sequence in the box in above skeleton vector can be replaced by the following sequences for constructing pDMP-miRNAs targeting particular genes:

miR-NT:
GAAATGTACTGCGCGTGGAGACGTTTTGGCCACTGACTGACGTCTCCACGCAGTACATTT
Human miR-FPN:
GTCTACCTGCAGCTTACATGATGTTTTGGCCACTGACTGACATCATGTACTGCAGGTAGA
Human miR-LCN2:
GTAATGTTGCCCAGCGTGAACTGTTTTGGCCACTGACTGACAGTTCACGGGGCAACATTA
Mouse miR-FPN:
GTATACAGACTCACTGATTTGCGTTTTGGCCACTGACTGACGCAAATCAGAGTCTGTATA
Mouse miR-LCN2:
GTCAAGTTCTGAGTTGAGTCCTGTTTTGGCCACTGACTGACAGGACTCATCAGAACTTGA

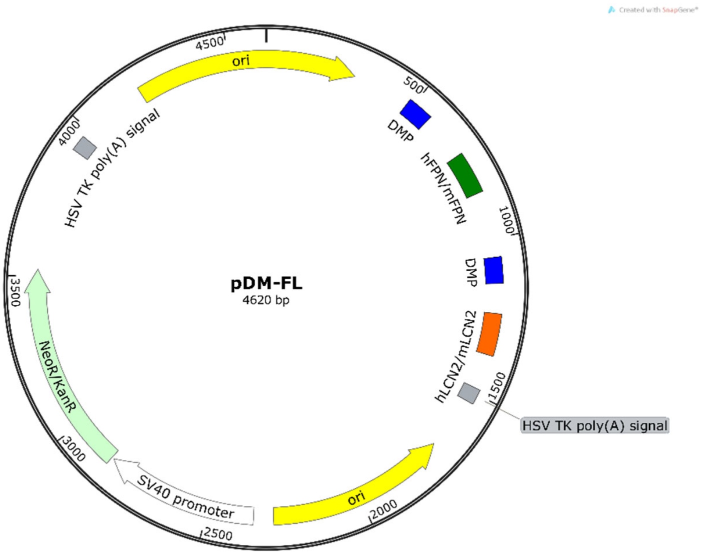

pDMP-miR-FPN-DMP-LCN2 (pDM-FL) (human)
DMP+ miR-hFPN +HSV TK poly(A) signal+DMP+ miR-hLCN2+ HSV TK poly(A) signal
GGGAATTTCCGGGGACTTTCCGGGAATTTCCGGGGACTTTCCGGGAATTTCCTAGAGGGTATAT
AATGGAAGCTCGACTTCCAGGCTAGCGAATTCGCTAAGCACTTCGTGGCCGTCGATCGTTTAA
AGGGAGGTAGTGAGTCGACCAGTGGATCCTGGAGGCTTGCTGAAGGCTGTATGCTGTCTACCT
GCAGCTTACATGATGTTTTGGCCACTGACTGACATCATGTACTGCAGGTAGACAGGACACAAG
GCCTGTTACTAGCACTCACATGGAACAAATGGCCCAGATCTGGCCGCACTCGAGATATCTAGAC
CCAGCTTTCTTGTACAAAGTGGTTGATCTAGAGGGCCCGCGGTTCGCTGATGGGGGAGGCTAA
CTGAAACACGGAAGGAGACAATACCGGAAGGAACCCGCGCTATGACGGCAATAAAAAGACAG
AATAAAACGCACGGGTGTTGGGTCGTTTGTTCATAAACGCGGGGTTCGGTCCCAGGGCTGGCA
CTCTGTCGATACCCCACCGTGACCCCATTGGGGCCAATACGCCCGCGTTTCTTCCTTTTCCCCA
CCCCACCCCCCAAGTTCGGGTGAAGGCCCAGGGCTCGCAGCCAACGTCGGGGCGGCAGGCCC
TGCCATAGGCGGCCGCAGGGAATTTCCGGGGACTTTCCGGGAATTTCCGGGGACTTTCCGGGA
ATTTCCTAGAGGGTATATAATGGAAGCTCGACTTCCAGGCTAGCGAATTCGCTAAGCACTTCGT
GGCCGTCGATCGTTTAAAGGGAGGTAGTGAGTCGACCAGTGGATCCTGGAGGCTTGCTGAAGG
CTGTATGCTGTAATGTTGCCCAGCGTGAACTGTTTTGGCCACTGACTGACAGTTCACGGGGCA
ACATTACAGGACACAAGGCCTGTTACTAGCACTCACATGGAACAAATGGCCCAGATCTGGCCG
CACTCGAGATATCTAGACCCAGCTTTCTTGTACAAAGTGGTTGATCTAGAGGGCCCGCGGTTCG
CTGATGGGGGAGGCTAACTGAAACACGGAAGGAGACAATACCGGAAGGAACCCGCGCTATGA
CGGCAATAAAAAGACAGAATAAAACGCACGGGTGTTGGGTCGTTTGTTC

pDMP-miR-FPN-DMP-LCN2 (pDM-FL) (mouse)
DMP+ miR-mFPN +HSV TK poly(A) signal+DMP+ miR-mLCN2+ HSV TK poly(A) signal
GGGAATTTCCGGGGACTTTCCGGGAATTTCCGGGGACTTTCCGGGAATTTCCTAGAGGGTATAT
AATGGAAGCTCGACTTCCAGGCTAGCGAATTCGCTAAGCACTTCGTGGCCGTCGATCGTTTAA
AGGGAGGTAGTGAGTCGACCAGTGGATCCTGGAGGCTTGCTGAAGGCTGTATGCTGTATACAG
ACTCACTGATTTGCGTTTTGGCCACTGACTGACGCAAATCAGAGTCTGTATACAGGACACAAG
GCCTGTTACTAGCACTCACATGGAACAAATGGCCCAGATCTGGCCGCACTCGAGATATCTAGAC
CCAGCTTTCTTGTACAAAGTGGTTGATCTAGAGGGCCCGCGGTTCGCTGATGGGGGAGGCTAA
CTGAAACACGGAAGGAGACAATACCGGAAGGAACCCGCGCTATGACGGCAATAAAAAGACAG
AATAAAACGCACGGGTGTTGGGTCGTTTGTTCATAAACGCGGGGTTCGGTCCCAGGGCTGGCA
CTCTGTCGATACCCCACCGTGACCCCATTGGGGCCAATACGCCCGCGTTTCTTCCTTTTCCCCA
CCCCACCCCCCAAGTTCGGGTGAAGGCCCAGGGCTCGCAGCCAACGTCGGGGCGGCAGGCCC
TGCCATAGGCGGCCGCAGGGAATTTCCGGGGACTTTCCGGGAATTTCCGGGGACTTTCCGGGA
ATTTCCTAGAGGGTATATAATGGAAGCTCGACTTCCAGGCTAGCGAATTCGCTAAGCACTTCGT
GGCCGTCGATCGTTTAAAGGGAGGTAGTGAGTCGACCAGTGGATCCTGGAGGCTTGCTGAAGG
CTGTATGCTGTCAAGTTCTGAGTTGAGTCCTGTTTTGGCCACTGACTGACAGGACTCATCAGAA
CTTGACAGGACACAAGGCCTGTTACTAGCACTCACATGGAACAAATGGCCCAGATCTGGCCGC

ACTCGAGATATCTAGACCCAGCTTTCTTGTACAAAGTGGTTGATCTAGAGGGCCCGCGGTTCGC
TGATGGGGGAGGCTAACTGAAACACGGAAGGAGACAATACCGGAAGGAACCCGCGCTATGAC
GGCAATAAAAAGACAGAATAAAACGCACGGGTGTTGGGTCGTTTGTTC
