# Supplementary material for: Gene interfered-ferroptosis therapy for cancers
Source: Nat Commun. 2021 Sep 7;12:5311. doi: 10.1038/s41467-021-25632-1 (PMC8423858; doi:10.1038/s41467-021-25632-1)
Supplement: Supplementary file 3 — Reporting Summary [file 41467_2021_25632_MOESM3_ESM.pdf]

nature portfolio

Corresponding author(s): Jinke Wang
Last updated by author(s): Jul 28, 2021

Reporting Summary

Nature Portfolio wishes to improve the reproducibility of the work that we publish. This form provides structure for consistency and transparency in reporting. For further information on Nature Portfolio policies, see our Editorial Policies and the Editorial Policy Checklist.

Statistics

For all statistical analyses, confirm that the following items are present in the figure legend, table legend, main text, or Methods section.

n/a	Confirmed	
☐	☒	The exact sample size (*n*) for each experimental group/condition, given as a discrete number and unit of measurement
☐	☒	A statement on whether measurements were taken from distinct samples or whether the same sample was measured repeatedly
☐	☒	The statistical test(s) used AND whether they are one- or two-sided<br>*Only common tests should be described solely by name; describe more complex techniques in the Methods section.*
☒	☐	A description of all covariates tested
☐	☒	A description of any assumptions or corrections, such as tests of normality and adjustment for multiple comparisons
☐	☒	A full description of the statistical parameters including central tendency (e.g. means) or other basic estimates (e.g. regression coefficient) AND variation (e.g. standard deviation) or associated estimates of uncertainty (e.g. confidence intervals)
☐	☒	For null hypothesis testing, the test statistic (e.g. *F*, *t*, *r*) with confidence intervals, effect sizes, degrees of freedom and *P* value noted<br>*Give P values as exact values whenever suitable.*
☒	☐	For Bayesian analysis, information on the choice of priors and Markov chain Monte Carlo settings
☒	☐	For hierarchical and complex designs, identification of the appropriate level for tests and full reporting of outcomes
☒	☐	Estimates of effect sizes (e.g. Cohen's *d*, Pearson's *r*), indicating how they were calculated

Our web collection on statistics for biologists contains articles on many of the points above.

Software and code

Policy information about availability of computer code

Data collection	CytExpert 2.3 software,Gen5 software ,Odyssey software, StepOne 2.3 software.
Data analysis	Data was analyzed in Graphpad Prism 8, imaging data was analyzed in Image-Pro Plus 6 and ImageJ 1.51j8 ,flow cytometry data was analyzed in CytExpert 2.3 software.

For manuscripts utilizing custom algorithms or software that are central to the research but not yet described in published literature, software must be made available to editors and reviewers. We strongly encourage code deposition in a community repository (e.g. GitHub). See the Nature Portfolio guidelines for submitting code & software for further information.

Data

Policy information about availability of data

All manuscripts must include a data availability statement. This statement should provide the following information, where applicable:

- Accession codes, unique identifiers, or web links for publicly available datasets
- A description of any restrictions on data availability
- For clinical datasets or third party data, please ensure that the statement adheres to our policy

All the data supporting the findings of this study are available within the article and its supplementary information files and from the corresponding author upon reasonable request. Source data are provided with this paper.

Field-specific reporting

Please select the one below that is the best fit for your research. If you are not sure, read the appropriate sections before making your selection.

[x] Life sciences   [ ] Behavioural & social sciences   [ ] Ecological, evolutionary & environmental sciences

For a reference copy of the document with all sections, see nature.com/documents/nr-reporting-summary-flat.pdf

Life sciences study design

All studies must disclose on these points even when the disclosure is negative.

Sample size	No statistical methods were used to predetermine the sample sizes. Details regarding sample size of all experiments were provided in the figure captions.
Data exclusions	No data was excluded from the analysis.
Replication	Experiments were repeated a minimum of one time to confirm the repeatability of the findings. All repeats of experiments were successful.
Randomization	All mice were randomly assigned to treatment or control groups for in vivo experiments.
Blinding	No blinding was done in this study.

Reporting for specific materials, systems and methods

We require information from authors about some types of materials, experimental systems and methods used in many studies. Here, indicate whether each material, system or method listed is relevant to your study. If you are not sure if a list item applies to your research, read the appropriate section before selecting a response.

Materials & experimental systems

n/a	Involved in the study	
[ ]	[x]	Antibodies
[ ]	[x]	Eukaryotic cell lines
[x]	[ ]	Palaeontology and archaeology
[ ]	[x]	Animals and other organisms
[x]	[ ]	Human research participants
[x]	[ ]	Clinical data
[x]	[ ]	Dual use research of concern

Methods

n/a	Involved in the study	
[x]	[ ]	ChIP-seq
[ ]	[x]	Flow cytometry
[x]	[ ]	MRI-based neuroimaging

Antibodies

Antibodies used	GAPDH Rabbit monoclonal antibody (ab181602, Abcam, UK,clone EPR16891), 1:10000 dilution<br>SLC40A1 Rabbit polyclonal antibody (ab58695, Abcam, UK), 1:1000 dilution<br>Lipocalin-2 Rabbit polyclonal antibody (ab63929, Abcam, UK),1:1000 dilution<br>IRDye® 800CW Goat anti-Rabbit IgG (C80118-05,Licor),1:10000 dilution
Validation	Validation for each antibody was performed in lab or referenced to the manufacturer's website. Validation statements are provided on the manufacturer's website.

Eukaryotic cell lines

Policy information about cell lines

Cell line source(s)	KG-1a, SKOV3, HMEC-1 and MCF-12A cell lines were acquired from American Type Culture Collection(ATCC). HL60, WEHI-3, HEK-293T, HepG2, A549, HT-29, C-33A, PANC-1, MDA-MB-453, Hepa1-6, B16F10, BGC-823/MGC-803/SGC-7901, KYSE450/KYSE510, CT-26, HL7702, L929, NIH-3T3, MRC-5 and GES-1 cell lines were obtained from the cell resource center of Shanghai Institutes for Biological Sciences, Chinese Academy of Sciences.
Authentication	All the cells were authenticated by by microscope for morphology, and for proper growth curve indicative of the strain.
Mycoplasma contamination	All cell lines tested negative for mycoplasma contamination.
Commonly misidentified lines (See ICLAC register)	No misidentified cell lines were used in this work.

Animals and other organisms

Policy information about studies involving animals; ARRIVE guidelines recommended for reporting animal research

Laboratory animals	BALB/C mice (four-week-old, female) and C57BL/6J mice (ten-week-old,female) were used in this study. The housing conditions for the mice is 12 light/12 dark cycle with temperatures of 22-25 °C and 50-70% humidity.
Wild animals	No wild animals were used in this study.
Field-collected samples	No field-collected samples were used in this study.
Ethics oversight	All animal experiments in this study followed the guidelines and ethics of the Animal Care and Use Committee of Southeast University (Nanjing, China).

Note that full information on the approval of the study protocol must also be provided in the manuscript.

Flow Cytometry

Plots

Confirm that:

- [x] The axis labels state the marker and fluorochrome used (e.g. CD4-FITC).
- [x] The axis scales are clearly visible. Include numbers along axes only for bottom left plot of group (a 'group' is an analysis of identical markers).
- [x] All plots are contour plots with outliers or pseudocolor plots.
- [x] A numerical value for number of cells or percentage (with statistics) is provided.

Methodology

Sample preparation	For cell death detection,cells were transfected with 500 ng of various plasmids ,the transfected cells were cultured for 24 h and then incubated with or without 50 μg/mL of FeNP, and cells were cultured for another 72 h, and then detected with the Annexin V-FITC Apoptosis Detection Kit (BD, USA).For ROS measurement,after cell were treat with 500 ng of various plasmids and FeNP for 48h,cells were stained with 2′,7′-dichlorodihydrofluorescein diacetate (DCFH-DA) using Reactive Oxygen Species Assay Kit (Beyotime) according to the manufacturer's instructions.
Instrument	CytoFLEX LX Flow Cytometer (Beckman) was used for flow cytometry data collection.
Software	CytExpert was used for data analysis.
Cell population abundance	At least 10,000 cells were analyzed for each condition.
Gating strategy	Cells were gated first by FSC/SSC, and then for FITC/PE.

- [x] Tick this box to confirm that a figure exemplifying the gating strategy is provided in the Supplementary Information.
